# Multi-wavelength activation of coupled ionophores for transmembrane electroneutral ion transport

**DOI:** 10.1039/d6cc06039k

**Published:** 2026-11-05

**Authors:** Kelly R. Britton, Srija Ghosh, Matthew J. Langton

**Affiliations:** a Chemistry Research Laboratory Mansfield Road Oxford OX1 3TA UK matthew.langton@chem.ox.ac.uk

## Abstract

Photo-activated synthetic ionophores are valuable tools for spatial and temporal regulation of transmembrane ion transport, with potential applications in therapeutics and the construction of stimuli-responsive artificial cells. Here we report the multi-wavelength activation of electroneutral transmembrane cation and anion co-transport facilitated by the coupled activity of a photo-caged synthetic anionophore and cationophore.

## Abstract 2

The multi-wavelength activation of electroneutral transmembrane cation and anion co-transport is reported, facilitated by the coupled activity of a photo-caged synthetic anionophore and cationophore.

## Graphical abstract

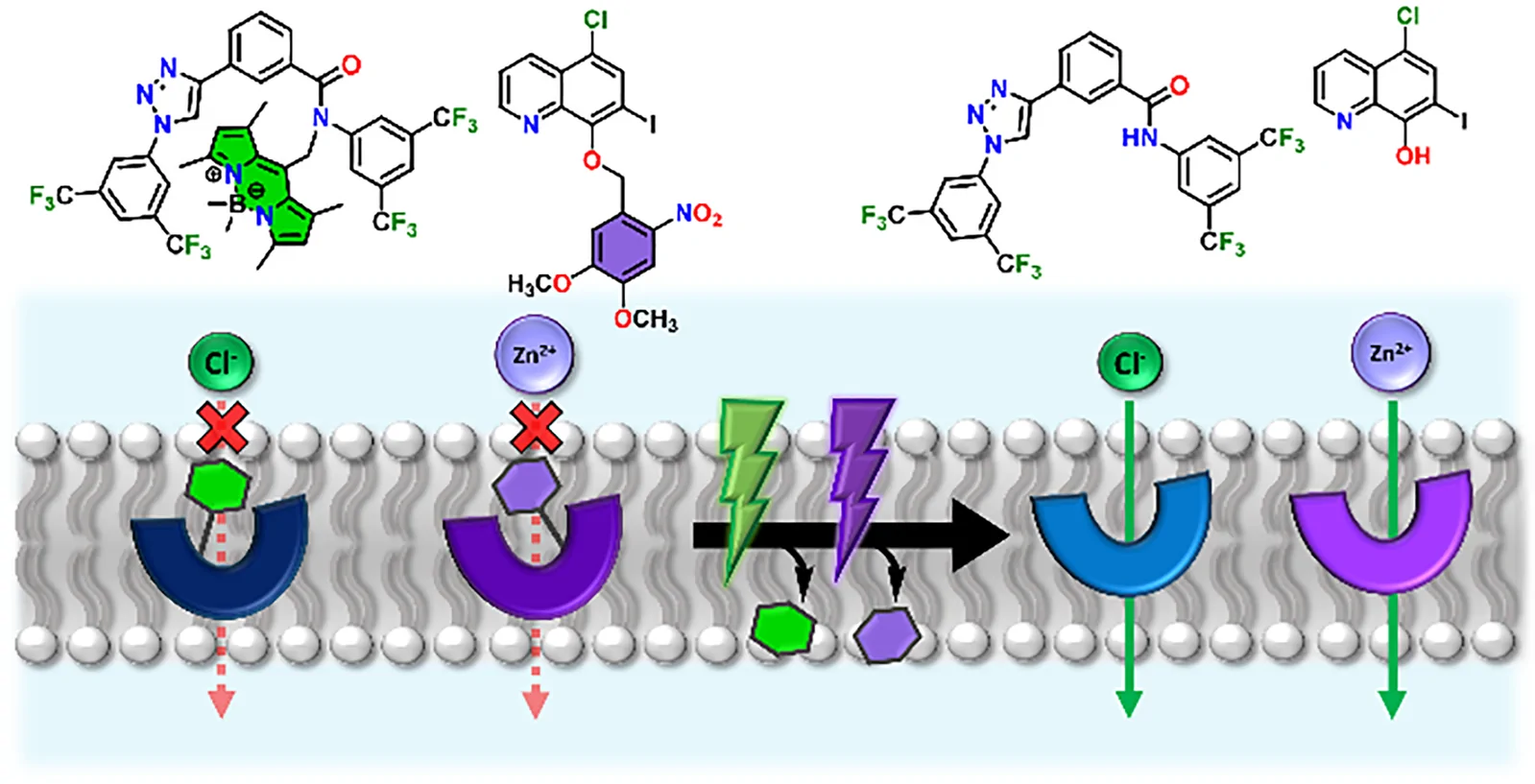

The selective movement of ions across lipid bilayer membranes is orchestrated by a range of membrane-embedded proteins, including ion channels, carriers, and pumps, which regulate essential biological functions.^1^ These systems operate through stimuli-dependent mechanisms responding to chemical signalling, membrane potential, concentration gradients, or light. Inspired by the sophistication of these natural processes, significant effort has been directed toward the development of synthetic ion transport systems capable of regulated transmembrane ion flux.^2,3^ In particular, the use of photo-responsive ionophores offers a promising strategy for achieving externally controllable ion transport.^4–7^ There are many biological systems where separate cation- and anion-transporting membrane proteins are coordinated to maintain electroneutrality and minimise large membrane potential changes, such as the regulation of transepithelial NaCl and fluid transport in epithelial tissues coordinated by epithelial sodium channel (ENaC) and cystic fibrosis transmembrane conductance regulator (CFTR) proteins,^8^ mediating charge balanced co-transport of Na^+^ and Cl^−^.^9^ In addition, CFTR functionally regulates ENaC activity, and disruption of this interaction contributes to altered ion and fluid homeostasis in diseases such as cystic fibrosis.^10^

Electroneutral ion transport studies using synthetic anionophores in conjunction with cationophores are relatively under-developed, and, to the best of our knowledge, are limited to those utilising naturally occurring cationophores. These include the K^+^ carrier valinomycin and the channel-forming peptide gramicidin. These are typically used to uncouple the antiport (exchange) or symport (co-transport) mechanisms of the anionophore under study,^11,12^ for example using valinomycin in the presence of K^+^ to investigate electrogenic chloride anionophores capable of chloride transport without coupled H^+^/OH^−^ transport.^13^

Herein, we demonstrate the first example of coupled cation and anion transport using two photo-responsive ionophores. We use a novel photocaged chloride anionophore and a photo-responsive variant of the cationophore clioquinol (CQ). Photocaging affords inactive states, with transport activity inhibited until irradiation with light cleaves the photocages, unblocking the binding sites and restoring transport activity. Functionalisation of the anionophore scaffold with a boron dipyrromethane (BODIPY) photocage, and of the cationophore CQ with an *ortho*-nitrobenzyl derivative, provides multi-wavelength control of electroneutral transport of both cations and anions *via* sequential irradiation (Fig. 1i).

**Fig. 1:**
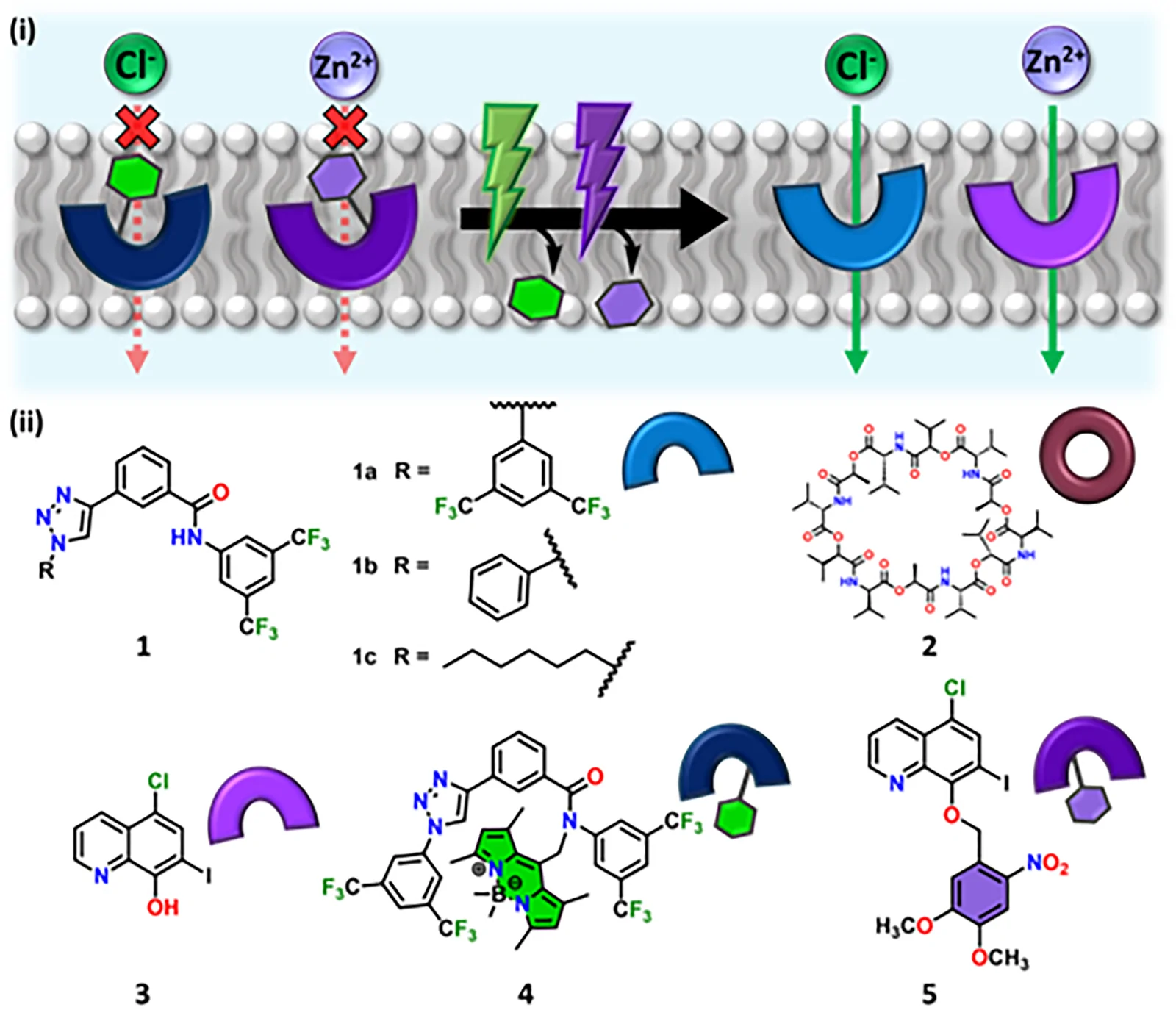
(i) Schematic depiction of photo-activated electroneutral transport facilitated by coupled anion and cation ionophores.(ii) Amido-triazole anionophores (1a–c), valinomycin (2), clioquinol (3), and photocaged anionophore (4) and cationophore (5).

The transporter scaffold (1, Fig. 1ii) was designed to incorporate an amide motif capable of both establishing strong hydrogen-bonding interactions with chloride anions and serving as a single site for protection with a photocleavable group. The design mimics an isophthalamide chloride receptor,^14^ but with one amide replaced by a triazole, a motif widely employed as *Z-trans* amide bioisosteres in medicinal chemistry and readily accessible *via* click chemistry.^15^ Furthermore, triazole-containing motifs have been demonstrated to function effectively as anion carriers,^16^ and in particular 3-(1*H*-1,2,3-triazol-1-yl)­benzamide anionophores have been reported by Talukdar and co-workers to show non-toxic behaviour in cell experiments.^17^ We employed a 3,5-bis-CF_3_-phenyl moiety to increase the acidity of the amide proton for enhanced anion binding,^18–20^ and varied the triazole substituent to tune anion binding, lipophilicity, and transport activity. Accordingly, we prepared a trio of amido-triazole anionophores (1a–c, Fig. 1ii). Full synthetic procedures and characterisation are available in the SI, Schemes S5–S7).

The chloride anion binding capabilities of the amido-triazoles were investigated by ^1^H NMR titration experiments in acetonitrile-*d*_3_ with tetrabutylammonium (TBA) chloride. The downfield chemical shift perturbations of both the amide and triazole proton signals were monitored and fitted to a 1 : 1 host–guest binding model using BindFit.^21^ As predicted on the basis of the expected electronic properties of the triazole substituent, *K*_1:1_(1a) > *K*_1:1_ (1b) > *K*_1:1_(1c), with binding affinities of *K*_1:1_ = 1055 ± 56 M^−1^, *K*_1:1_ = 742 ± 15 M^−1^, and *K*_1:1_ = 403 ± 16 M^−1^ respectively (Fig. S20–S22).

Next, chloride transport by 1a, which exhibited strong chloride binding, was investigated using the well-established HPTS assay in large unilamellar vesicles (LUVs) formed from 1 palmitoyl-2-oleoyl-*sn*-glycero-3-phosphocholine (POPC LUVs, lipid concentration 31 µM), encapsulating 8-hydroxypyrene-1,3,6-trisulfonic acid trisodium salt (HPTS).^22^1a proved to be a highly active chloride transporter (Fig. 2i) with an EC_50_ = 13 nM (Fig. S33), outperforming previous amido-triazole anionophores by around a factor of 60.^17^

**Fig. 2:**
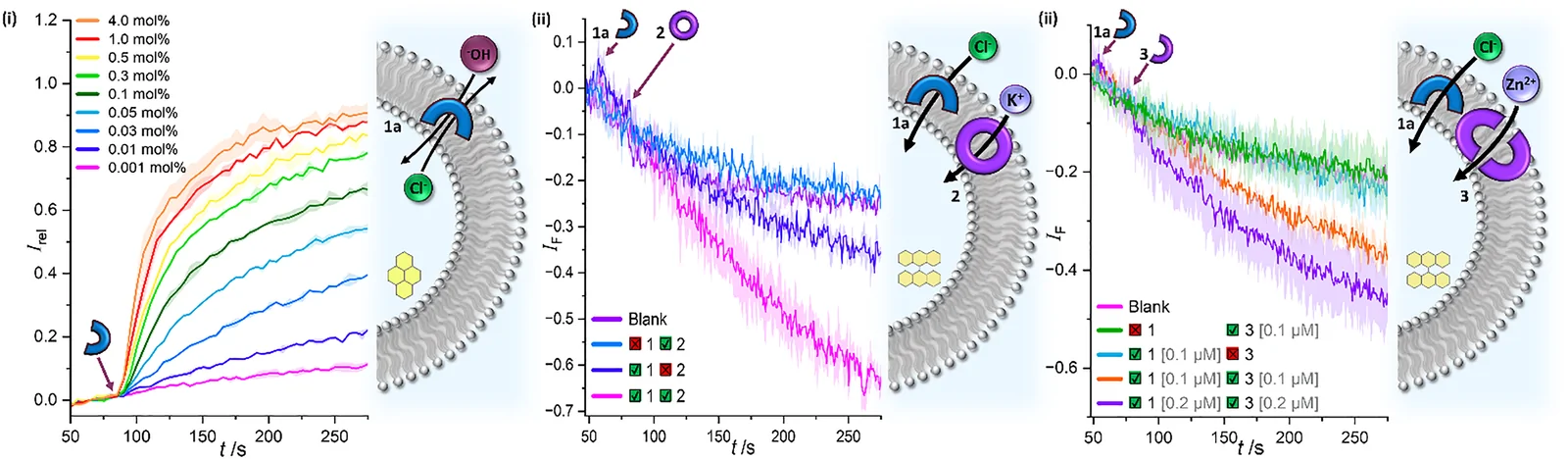
Ion transport assays with 1a. (i) HPTS pH dissipation assay. Change in ratiometric emission, *I*_rel_ (*λ*_em_ = 510 nm; *λ*_ex1_ = 405 nm, *λ*_ex2_ = 460 nm) upon addition of a NaOH base pulse (5 mM) to POPC LUVs (31 µM) containing 1 mM HPTS, in 100 mM NaCl, 10 mM HEPES at pH 7.0. (ii) KCl transport assay with 1a and valinomycin and (iii) ZnCl_2_ transport with 1a and clioquinol 3 using the lucigenin assay. Data shows change in fluorescence intensity, *I*_F_ (*λ*_em_ = 505 nm; *λ*_ex_ = 430 nm) with addition of a KCl or ZnCl_2_ pulse (30 mM) with 1a (0.1 µM, 0.3 mol%) and valinomycin 2 (0.1 µM) or clioquinol 3 (various concentrations) to POPC LUVs containing 1 mM lucigenin, 100 mM Na_2_SO_4_, buffered with 20 mM MES at pH 6.5.

Mechanistic studies were performed to further elucidate the transport mechanism, mode, and selectivity of 1a. Experiments using LUVs formed from dipalmitoylphosphatidylcholine (DPPC) determined that 1a acts as a carrier, indicated by diagnostic activity in the lipid gel phase, and restoration of activity above the phase transition temperature (SI 6.1.2). Anion exchange revealed an apparent selectivity of Cl^−^ > NO_3_^−^ > Br^−^ > I^−^ (SI 6.1.3) and transport was inhibited in the presence of gluconate (a large hydrophilic anion typically unable to be transported), thus ruling out potential Na^+^/H^+^ antiport and/or Na^+^/OH^−^ symport and indicating that transport is independent of metal cations (SI 6.1.4). This was further confirmed by the observation that activity was invariant to exchange of the cation (M = Na, K, Li, Rb, Cs), (SI 6.1.5). Finally, the selectivity of 1a for Cl^−^ over H^+^/OH^−^ transport was assessed using the protonophore carbonyl cyanide 4-(trifluoromethoxy)phenylhydrazone (FCCP), which uncouples H^+^/OH^−^ transport from Cl^−^ transport. The activity of 1a was unaffected by FCCP, indicating no selectivity for Cl^−^ over H^+^/OH^−^ (SI 6.1.6).

A proof-of-concept experiment to evaluate electroneutral transmembrane transport by coupled ionophores was conducted with the K^+^ carrier valinomycin (2) and anionophore 1a. Valinomycin is typically used in mechanistic studies to determine the mechanism of Cl^−^ transport by decoupling any K^+^ dependent antiport or symport processes of the anionophore.^23^ Here we substituted the Cl^−^ in the buffer to SO_4_^2−^, which is not transported, to inhibit the ability of the anionophore to facilitate H^+^/anion symport or anion/^−^OH antiport, but maintain electroneutral transport by coupling Cl^−^ ion flux to K^+^ co-transport mediated by valinomycin (2). Using POPC LUVs encapsulating the chloride-sensitive *N*,*N*′-dimethyl-9,9′-biacridinium dinitrate (lucigenin) fluorophore, coupled K^+^/Cl^−^ transport was monitored following a KCl pulse. Only upon addition of both 1a and 2 was chloride transport facilitated, observed by the quenching of lucigenin's fluorescence (Fig. 2ii). A control experiment using either NaCl or ZnCl_2_ pulses showed no chloride-quenching of the internal lucigenin, attributed to unfavourable transport of Na^+^ or Zn^2+^ by 2 (Fig. S43). Following this successful demonstration of coupled cation–anion transport, 2 was replaced with the quinoline-based cationophore, clioquinol (3).^24,25^ Electroneutral transport of ZnCl_2_, coupled by 1a and 3 was investigated using the same lucigenin assay protocol, substituting the KCl pulse with ZnCl_2_ (Fig. 2iii). Only in the presence of both 1a and 3 (both at 0.1 µM) was transport observed, with activity increasing when both were increased to 0.2 µM loading.

Both 1a and 3 are amenable to further functionalisation by alkylation, and therefore we next explored the possibility of exerting photo-control over the coupled electroneutral MCl transport process by attaching photolabile protecting groups (photocages). Alkylation of a photocage to the functional group of an ionophore involved in ion binding enables deactivation of transport by inhibiting binding,^26–29^ which is then restored upon irradiation to activate transport. We have previously shown that control of activity of an individual anionophore could be achieved by sequential irradiation of two different photocages at two specific wavelengths of light.^27^ Chloride anionophore 1a was photocaged with boron dipyrromethane (BODIPY) to generate caged transporter 4, and cationophore 3 with a dimethoxy *ortho*-nitrobenzyl (DMNB) photocage to generate 5 (Fig. 1ii). Photo-activated ion transport was initially examined separately for 4 using the HPTS assay, and 5 using the zinc-sensitive magnesium green (MgG) assay.^24^ Irradiation of 4 with 530 nm light restored chloride transport, with increasing activity up to ∼60% observed after 360 seconds *in situ* irradiation, and after just 90 seconds of *ex situ* irradiation (Fig. S48). Following irradiation of 5 with 365 nm light, ∼50% zinc transport was reached after 90 seconds *in situ*, and after 10 seconds of *ex situ* irradiation (Fig. S49).

With photo-activated ion transport separately confirmed for both ionophores, photo-control of electroneutral Zn^2+^/Cl^−^ transport was investigated using the photostable chloride-sensitive probe 6-methoxy-*N*-(3-sulfopropyl)-quinolinium (SPQ, Fig. S51), in place of lucigenin which is sensitive to photo-degradation.^30^ Photocaged ionophores 4 and 5 were incubated in POPC LUVs encapsulating SPQ. As expected, no transport activity was observed (Fig. 3i). Similarly, irradiating only at 530 nm did not activate transport, as it is unable to decage compound 5, hence preventing coupled Zn^2+^/Cl^−^ co-transport (Fig. 3ii). Transport activity was restored upon sequential *in situ* irradiation with the higher wavelength of 530 nm first, to cleave the BODIPY cage from 4 to generate 1a, followed by 365 nm, to cleave DMNB from 5 to generate 3 (Fig. 3ii, purple data). Irradiation with solely 365 nm light activated the ion symport process by decaging both ionophores (blue data), due to overlap of the respective absorption spectra below 400 nm of the BODIPY and DMNB photocages of 4 and 5 (Fig. S23).

**Fig. 3:**
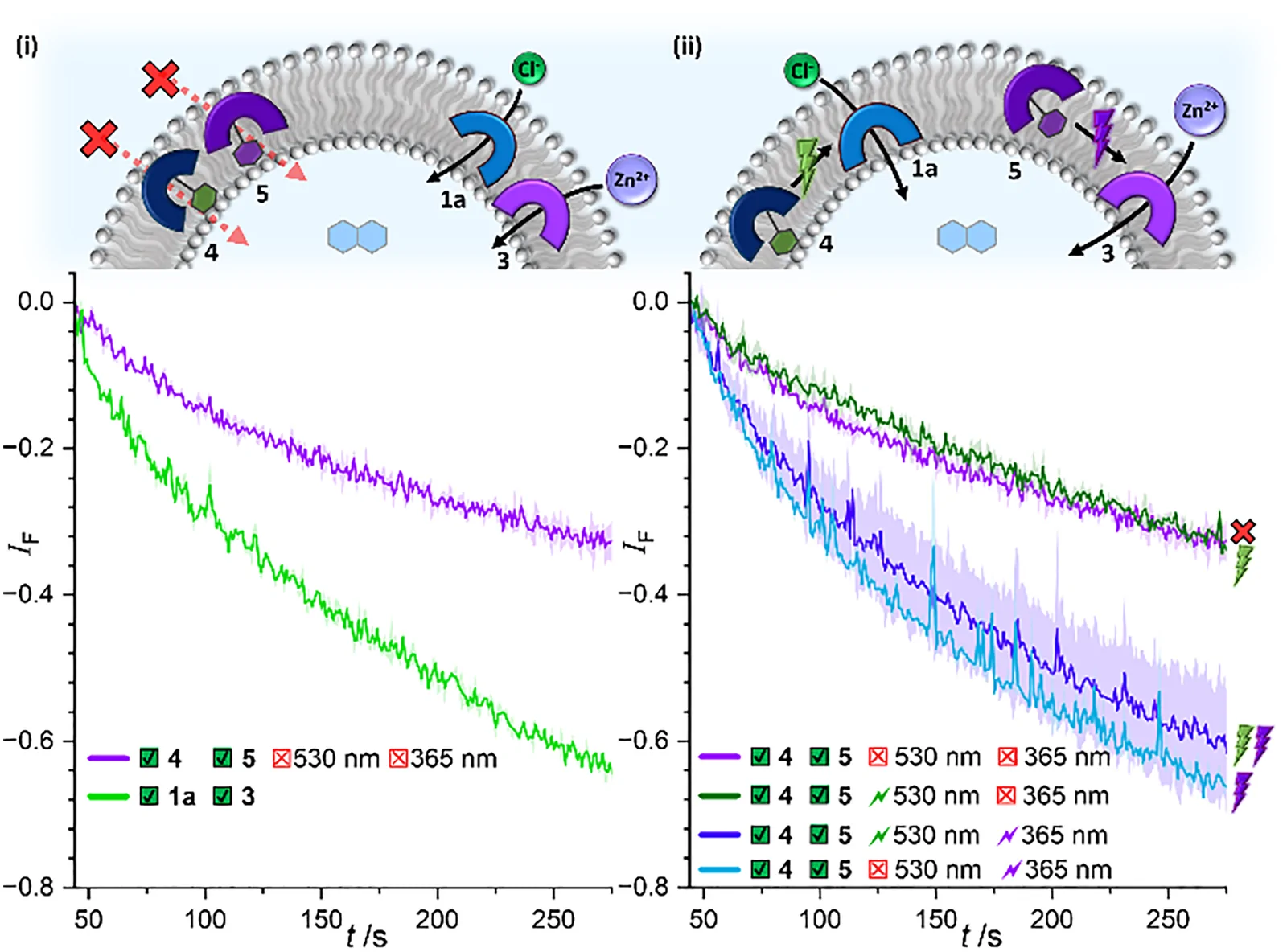
Multiwavelength activation of the coupled-electroneutral ion transport assay with SPQ. (i) Transport of caged ionophores 4 and 5 in comparison to decaged analogues 1a and 3; (ii) sequential photo-decaging experiments with 4 and 5. Data shows change in fluorescence intensity, *I*_F_ (*λ*_em_ = 450 nm; *λ*_ex_ = 320 nm) following *in situ* irradiation with a 530 nm LED (370 mW) and/or a 365 nm LED (880 mW) of BODIPY-photocaged anionophore 4 (400 nM, 1.2 mol% w.r.t. lipid) and DMNB-photocaged cationophore 5 (400 nM, 1.2 mol% w.r.t. lipid), and 4 and 5 prior to any irradiation. At 40 seconds, a ZnCl_2_ pulse (30 mM) was added to the POPC LUVs (33 µM) containing 1 mM SPQ, 100 mM internal and 70 mM external Na_2_SO_4_, buffered with 20 mM MES at pH 6.5.

The sequential activation of 4 and 5 by successive application of 530 nm and 365 nm light was confirmed by photodecaging analysis using ^1^H NMR experiments (Table 1 and SI 5.2). These experiments revealed selective decaging of 4 at 530 nm, due to no overlap of the LED emission spectrum with its absorbance spectrum, in agreement with results from the transport assays. The apparent slower decaging of BODIPY-derivative 4 compared to DMNB-caged 5 at 365 nm irradiation correlates to the reduced absorption of 4 at that wavelength.

**Table 1:** Photo-decaging half-lives (*t*_1/2_) for 4 and 5 determined by ^1^H NMR analysis (1 mM in DMSO-*d*_6_, 298 K) using 365 nm LED (880 mW) and 530 nm LED (370 mW) LEDs

Compound	*t*_1/2_ (365 nm)/s	*t*_1/2_ (530 nm)/s
4	660	240
5	120	No decaging

In conclusion, novel amido-triazole anionophores were synthesised and coupled with a cationophore to facilitate electroneutral ion transport across lipid bilayer membranes. Photocaging of both ionophores enabled wavelength-selective control of transport, with the photocaged systems exhibiting negligible activity. Sequential irradiation, first at 530 nm and then with 365 nm, released the respective ionophores, restoring coupled anion–cation transport. This multi-wavelength strategy provides a platform for spatiotemporal control of electroneutral transmembrane ion transport and may inform the development of photo-targeted systems capable of modulating intracellular ionic homeostasis for therapeutic applications.

## Author contributions

Conceptualisation, K. R. B. and M. J. L.; data curation, S. G. and K. R. B.; writing – original draft, K. R. B., writing – review & editing, K. R. B. and M. J. L.; project administration and supervision, M. J. L.

## Conflicts of interest

There are no conflicts to declare.

## Supplementary Material

CC-OLF-D6CC06039K-s001

## Data Availability

The data supporting this article have been included as part of the supplementary information (SI). Supplementary information: synthesis and characterisation, experimental methods and data. See DOI: https://doi.org/10.1039/d6cc06039k.
