# Supplementary material for: Multi-wavelength activation of coupled ionophores for transmembrane electroneutral ion transport
Source: Chem Commun (Camb). 2026 Nov 5. Online ahead of print. doi: 10.1039/d6cc06039k (PMC13754030; doi:10.1039/d6cc06039k)
Supplement: Supplementary file 1 [file CC-OLF-D6CC06039K-s001.pdf]

Supplementary Information (SI) for Chemical Communications.
This journal is © The Royal Society of Chemistry

Supporting Information for

**Multi-wavelength Activation of Coupled Ionophores for Transmembrane Electroneutral Ion Transport**

Kelly R. Britton [a], Srija Ghosh [a], and Matthew J. Langton*[a]

[a] Department of Chemistry, University of Oxford, 12 Mansfield Road, Oxford, OX1 3TA, UK

## Contents

Contents ..... 1

1. Materials and Methods ..... 2

1.1 Abbreviations ..... 3

2 Synthesis and Characterisation ..... 4

3 $^1$H NMR Titration Experiments ..... 22

4 Photophysical Analysis ..... 25

4.1 UV-Vis Absorbance ..... 25

4.2 Fluorescence Excitation and Emission ..... 27

5 Photocleavage Experiments ..... 28

5.1 Irradiation Set-up ..... 28

5.2 $^1$H NMR Analysis ..... 29

6 Anion Transport Experiments ..... 32

6.1 Vesicle Preparation for HPTS Assays[11] ..... 32

6.1.1 The Standard HPTS Assay ..... 33

6.1.2 HPTS Assay with DPPC Lipids ..... 35

6.1.3 HPTS Assay with NaX Buffers ..... 36

6.1.4 HPTS Assay with Sodium Gluconate ..... 37

6.1.5 HPTS Assay with MCl Buffers ..... 38

6.1.6 HPTS Assay with FCCP ..... 39

6.2 Vesicle Preparation for Magnesium Green (MgG) Assays[7] ..... 40

6.2.1 The Magnesium Green (MgG) Assay ..... 41

6.3 Vesicle Preparation for Lucigenin Assays[12] .......... 41

6.3.1 The Standard Lucigenin Assay .......... 42

6.3.2 The Coupled-Electroneutral Transport Lucigenin Assay .......... 44

6.4 Vesicle Preparation for SPQ Assays[13, 14] .......... 47

6.4.1 The Coupled-Electroneutral Transport SPQ Assay .......... 48

6.5 Photo-triggered Transport Assays .......... 49

7 References .......... 54

# 1. Materials and Methods

All reagents and solvents were purchased from commercial sources and used without further purification. Lipids were purchased from Avanti Polar Lipids and used without further purification. Where necessary, solvents were dried by passing through an MBraun MPSP-800 column and degassed with nitrogen. Triethylamine was distilled from and stored over potassium hydroxide. Column chromatography was carried out on Merck® silica gel 60 under a positive pressure of nitrogen. Where mixtures of solvents were used, ratios are reported by volume. GPC purification of vesicles was carried out using GE Healthcare PD10 desalting columns prepacked with Sephadex G 25 medium. NMR spectra were recorded on a Bruker AVIII 400, Bruker AVII 500 (with cryoprobe) and Bruker AVIII 500 spectrometers. Chemical shifts are reported as δ values in ppm. Mass spectra were carried out on a Waters Micromass LCT and Bruker microTOF spectrometers. Fluorescence spectroscopic data were recorded using a Horiba Duetta or Agilent Cary Eclipse fluorescence spectrophotometer, equipped with Peltier temperature controller and stirrer. UV-Vis absorption spectra were recorded on a V-770 UV-Visible/NIR Spectrophotometer equipped with Peltier temperature controller and stirrer. Vesicles were prepared as described below using Avestin "LiposoFast" extruder apparatus, equipped with polycarbonate membranes with 200 nm pores. When using lipids with a phase transition temperature above RT, the Avanti Mini Extruder (equipped with a heating block) was used instead.

## 1.1 Abbreviations

**BODIPY:** Boron-dipyrromethene

**DCM:** Dichloromethane

**DIAD:** diisopropyl azodicarboxylate

**DIPEA:** *N,N*-Diisopropylethylamine

**DMF:** Dimethylformamide

**DPPC:** 1,2- Dipalmitoyl-*sn*-glycero-3-phosphocholine

**EDTA:** Ethylenediaminetetraacetic acid

**$Et_2O$:** Diethyl ether

**EtOAC:** Ethyl acetate

**EtOH:** Ethanol

**FCCP:** Carbonyl cyanide-p-trifluoromethoxyphenylhydrazone

**HEPES:** 4-(2-Hydroxyethyl)-1- piperazineethanesulfonic acid

**HOAc:** Acetic acid

**HPTS:** 8-Hydroxypyrene-1,3,6-trisulfonic acid trisodium salt

**HRMS:** High resolution mass spectrometry

**$H_2SO_4$:** Sulfuric acid

**Lucigenin:** *N*,*N*′-dimethyl-9,9′-biacridinium dinitrate

**LUV:** Large unilamellar vesicles

**MgG:** Magnesium green pentapotassium salt

**$MgSO_4$:** Magnesium sulfate

**$NaHCO_3$:** Sodium bicarbonate

**$NaN_3$:** Sodium azide

**$NaNO_2$:** Sodium nitrite

**$PBr_3$:** Phosphorous tribromide

**POPC:** 1-Palmitoyl-2- oleoyl-*sn*-3-phosphatidylcholine

**$PPh_3$:** triphenylphosphine

**rt:** Room temperature

**SPQ:** 6-methoxy-*N*-(3-sulfopropyl)quinolinium

**TFA:** Trifluoroacetic acid

**THF:** Tetrahydrofuran

# 2 Synthesis and Characterisation

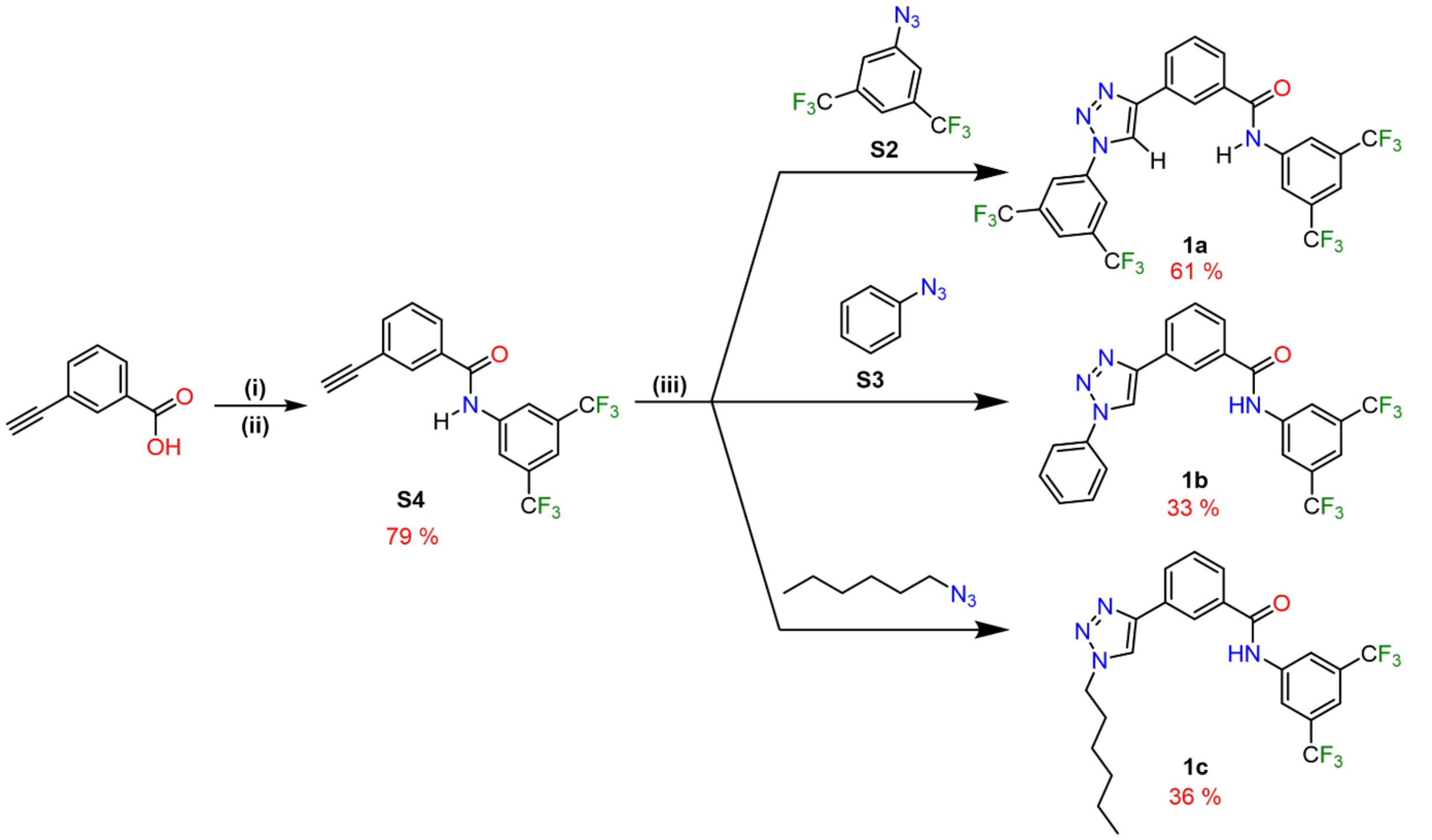


**Scheme 1:** Overall synthetic route to amido-triazole based anionophores, **1a-c**. Synthesis of alkyne scaffold **S4** *via* **(i)** $C_2O_2Cl_2$, DCM, 1% DMF, 0 °C - rt, 2 h, **(ii)** 3,5-bis(trifluoromethyl)aniline, DCM, DIPEA, 0 °C - rt, 24 h. Anionophore products **1a-c** obtained *via* **(iii)** TBTA, $Cu(CH_3CN)_4PF_6$, DCM, corresponding azide, 0 °C - rt, 24 h.

**General click reaction procedure for the synthesis of triazole anionophores:**

$Cu(MeCN)_4PF_6$ and TBTA were stirred in DCM under a nitrogen atmosphere. Alkyne and the azide were added to the reaction and stirred for 24 hours. The reaction was then diluted with DCM and the organic phase was washed with aqueous 1% EDTA/$NH_4OH$ solution and brine, then dried over $MgSO_4$ and concentrated *in vacuo.*

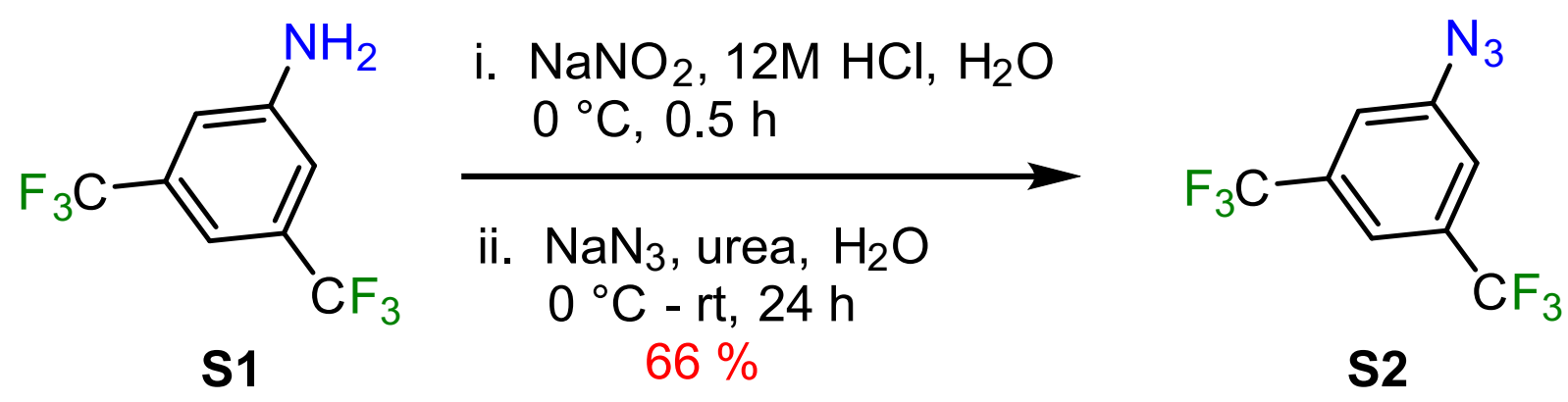


**Scheme 2:** Synthesis of 3,5-bis(trifluoromethyl)phenylazide **S2**

Prepared by a modified literature procedure.[1] 3,5-bis(trifluoromethyl)aniline **S1** (2.00 mL, 12.8 mmol, 1 equiv.) was dissolved in deionised water (13.0 mL) and HCl (13.0 mL) at 0 °C. Sodium nitrite ($NaNO_2$) (1.06 g, 15.4 mmol, 1.2 equiv.) was dissolved in deionised water (13.0 mL) at 0 °C. The solution of $NaNO_2$ was added dropwise to the solution of aniline and stirred at 0 °C for 2 hrs. Sodium azide ($NaN_3$) (1.25 g, 19.2 mmol, 1.5 equiv.) was dissolved in deionised water (6.5 mL) at 0 °C. The solution of $NaN_3$ (1.5 equiv.) in water (0.5 mL per 1 mmol of aniline) was added dropwise at 0 °C, and the reaction was stirred for 2 hrs and allowed to warm to rt. The organic layer was extracted twice with $Et_2O$ (2 x 10 mL) and the combined organic layers were washed with water (2 x 10 mL), $NaHCO_3$ (2 x 10 mL), brine (2 x 10 mL), dried over $MgSO_4$, filtered and concentrated under reduced pressure to yield the corresponding pure aryl azide **S2** as a yellow/orange oil (2.2 g, 8.4 mmol, 66 %). $^1H$ NMR (400 MHz, $CDCl_3$) δ 7.64 (s, 1H), 7.44 (s, 2H). HRMS-ESI (m/z) Calculated for $C_8H_3F_9N_3$ $[M+H]^+$, 256.0 304; found 256.2649. Data consistent with the literature.[1]

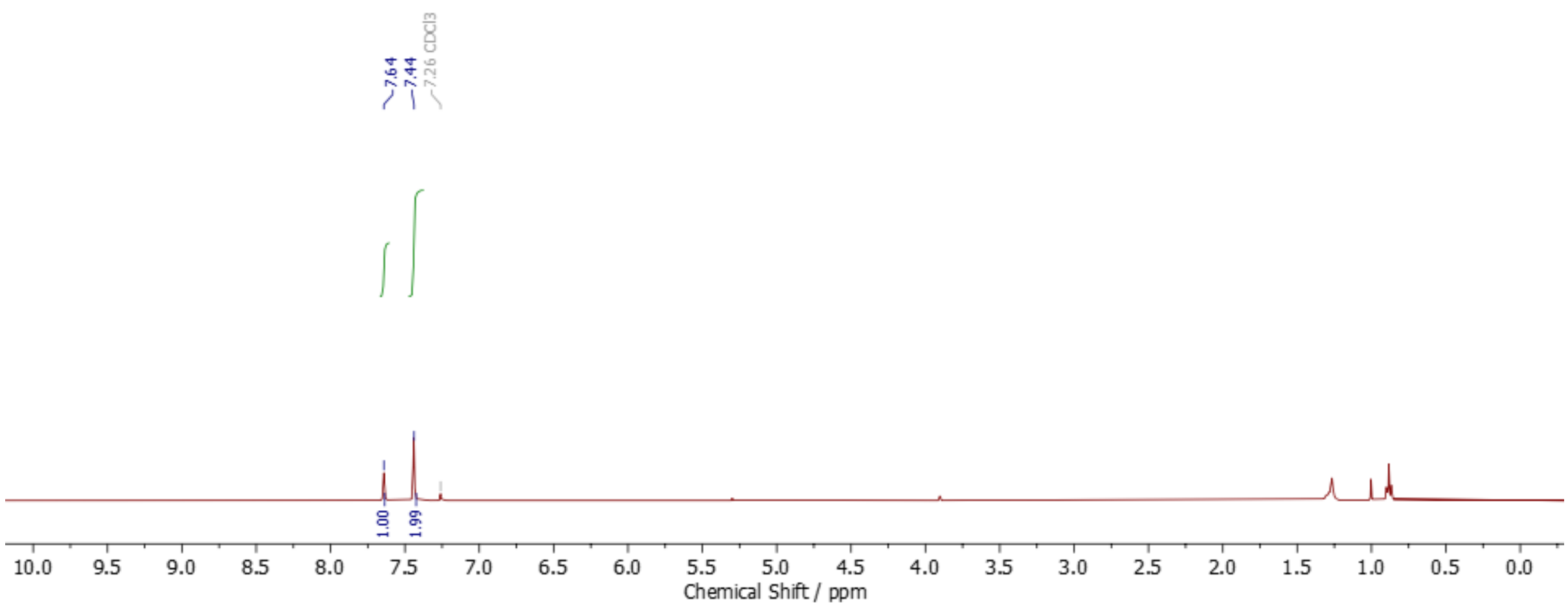


**Figure SI-1**: $^1H$ NMR Spectrum of **S2** ($CDCl_3$, 298 K, 400 MHz)

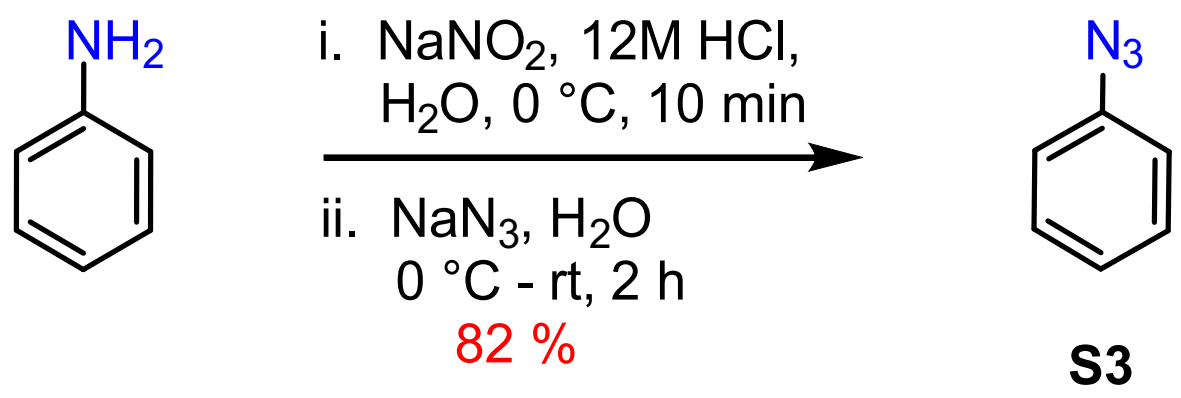

**Scheme 3:** Synthesis of azidobenzene **S3**

Prepared by a modified literature procedure.[1] Aniline (490 µL, 5.37 mmol, 1 equiv.), 12 M HCl (2.4 mL), water (2.4 mL) and ice (5 g) were combined in an ice bath. A solution of $NaNO_2$ (370 mg, 5.37 mmol, 1 equiv.) in water (2 mL) was slowly added to the above stirred mixture, maintaining a temperature below 10˚C. After 10 minutes, $NaN_3$ (393 mg, 6.04 mmol, 1.6 equiv.) in water (1 mL) was added to the reaction and after 10 minutes the ice bath was removed and the reaction mixture stirred at rt for 1 hour. The organic layer was extracted with $Et_2O$ (2 x 25 mL), washed with $NaHCO_3$ (2 x 25 mL), water (2 x2 5 mL) and dried over $MgSO_4$, filtered and concentrated under reduced pressure to yield the corresponding azide product **S3** (525 mg, 4.41 mmol, 82 %), which was protected from light and reacted on without further purification. $^1$H NMR (400 MHz, $CDCl_3$) δ 7.39 (d, J = 7.5 Hz, 2H), 7.17 (dd, J = 7.5 Hz, 1H), 7.06 (d, J = 7.5 Hz, 2H). HRMS ESI (m/z) Calculated for $C_6H_5N_3$ $[M+K]^+$, 158.0115 ; found 158.0112. Data consistent with the literature.[1]

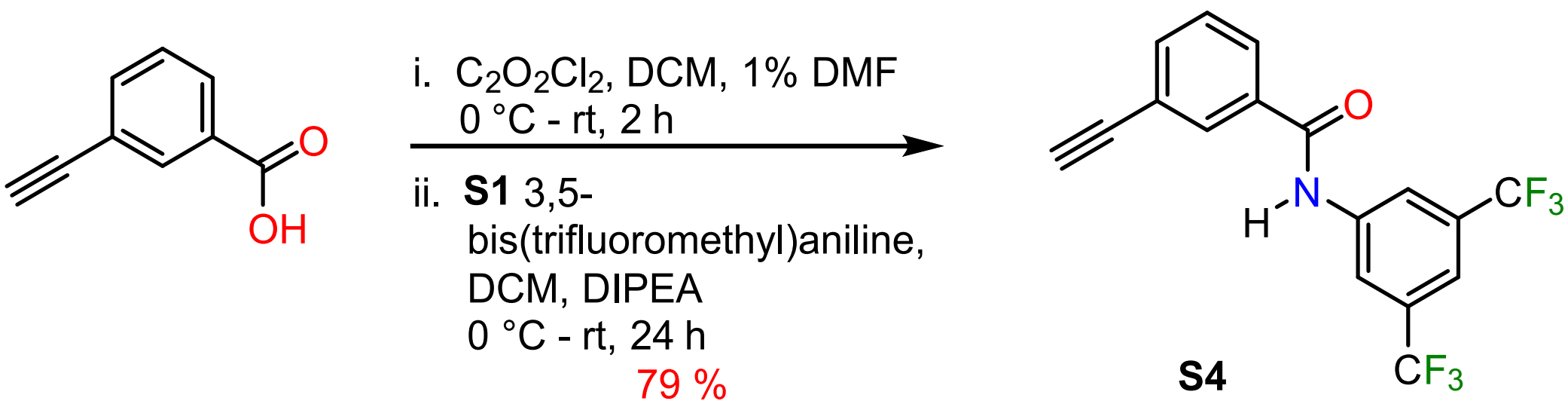


**Scheme 4:** Synthesis of *N*-(3,5-bis(trifluoromethyl)phenyl)-3-ethynylbenzamide **S4**

Prepared by a modified literature procedure.[2] To a stirred solution of 3-ethynylbenzoic acid (126 mg, 0.86 mmol, 1 equiv.) in dry DCM (10 mL) was added a few drops of DMF and oxalyl chloride (0.1 mL, 1.3 mmol, 1.5 equiv.) at 0˚C. After warming to rt, the mixture was allowed to stir for 2 hrs and then the solvent was removed *in vacuo*. The intermediate acid chloride was redissolved in dry DCM (10 mL) and cooled to 0 ˚C. 3,5 bis(trifluoromethyl)aniline **S1** (0.2 mL, 1.3 mmol, 1.5 equiv.) was added followed by DIPEA (0.2 mL, 1.1 mmol, 1.3 equiv.) after 30 mins. The reaction was stirred at rt for 24 hrs, solvent removed *in vacuo* and resuspended in EtOAc (25 mL). The organic layer was extracted with 1 M HCl (25 mL), water (25 mL) and brine (25 mL) and consequently dried over $MgSO_4$. The solvent was removed under reduced pressure. The crude compound was purified by silica flash column chromatography in hexane:DCM 1:1 to afford a white solid **S4** (244 mg, 0.68 mmol, 79 %). $^1$H NMR (400 MHz, $CDCl_3$) δ 8.18 (s, 2H), 7.98 (s, 1H), 7.89 (d, *J* = 7.8 Hz, 1H), 7.72 (d, *J* = 7.8 Hz, 1H), 7.67 (s, 1H), 7.51 (t, *J* = 7.8 Hz, 1H), 3.19 (s, 1H). HRMS-ESI (m/z) Calculated for $C_{17}H_{10}F_6NO$ $[M+H]^+$, 358.0661; found 358.0669. Data consistent with the literature.[2]

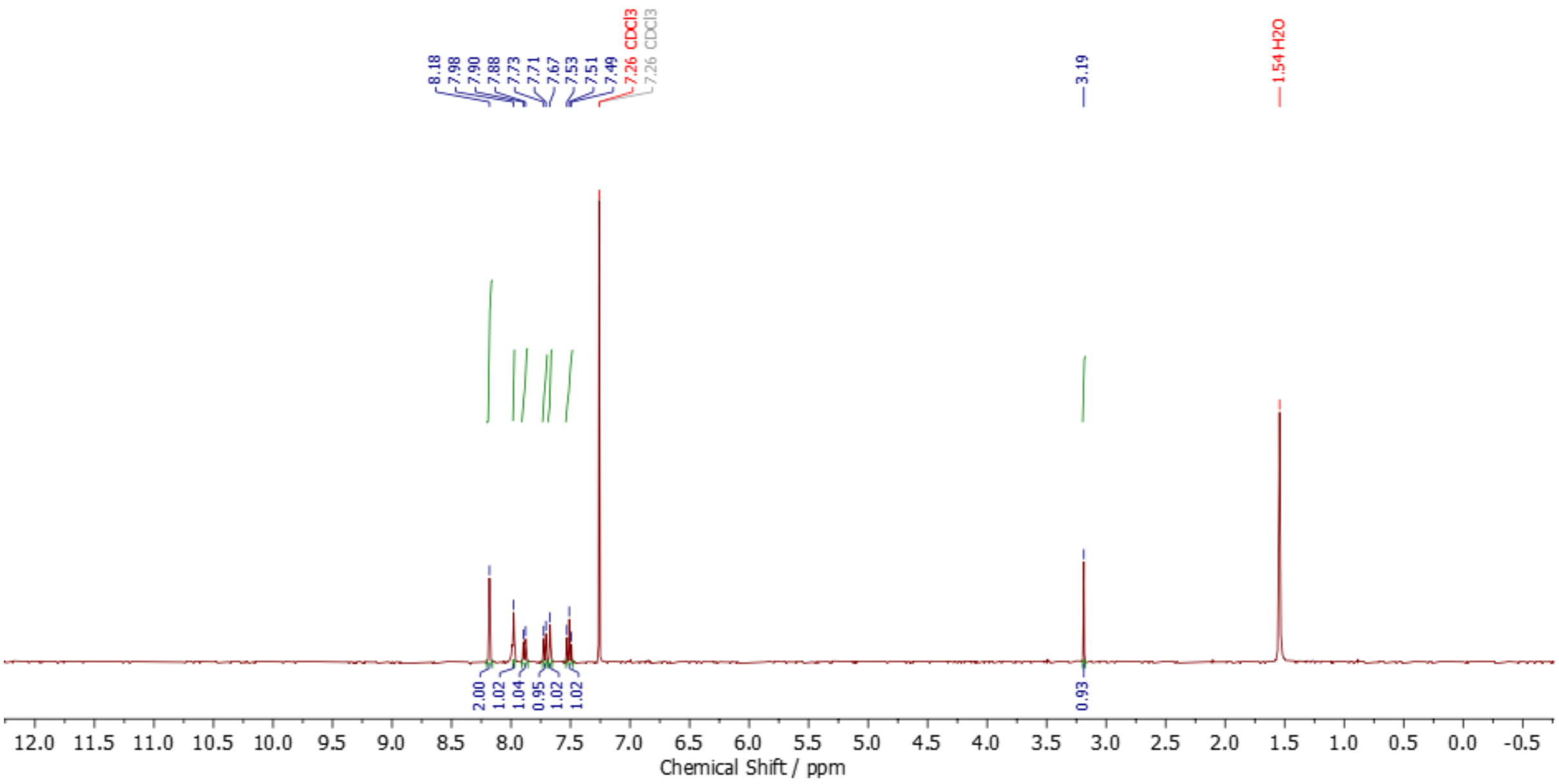


**Figure SI-2**: $^1$H NMR Spectrum of **S6** ($CDCl_3$, 298 K, 400 MHz)

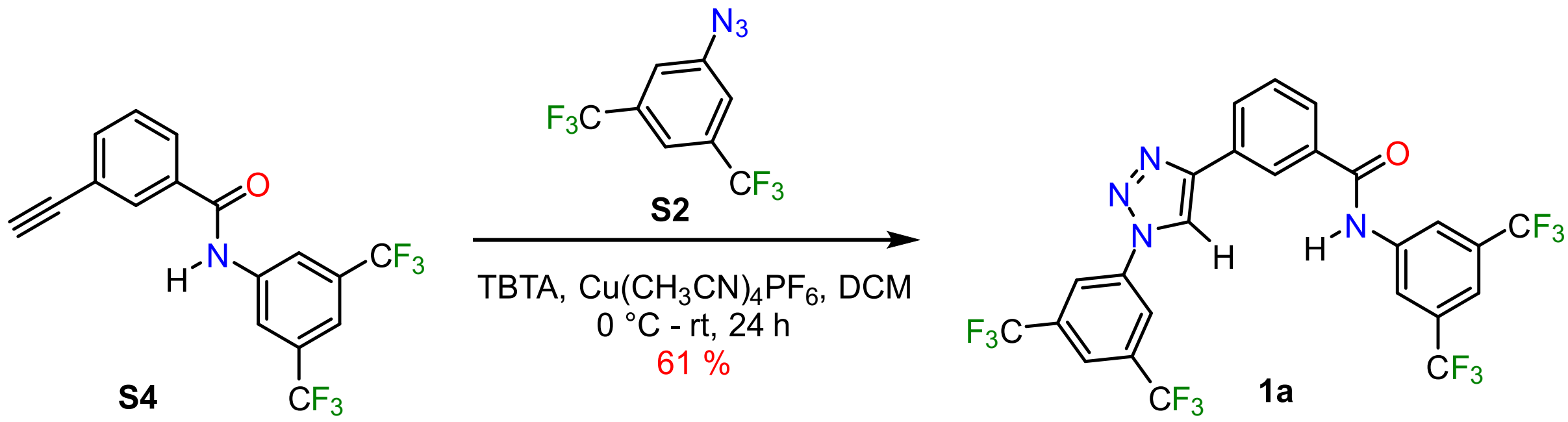


S4 1a

**Scheme 5:** *N*-(3,5-bis(trifluoromethyl)phenyl)-3-(1-(3,5-bis(trifluoromethyl)phenyl)-1*H*-1,2,3-triazol-4-yl) benzamide (Amido-triazole anionophore) **1a**

General click reaction procedure was followed. $Cu(MeCN)_4PF_6$ (101 mg, 0.27 mmol, 0.05 equiv.), TBTA (143 mg, 0.27 mmol, 0.05 equiv.), were stirred for 30 minutes in DCM (20 mL) under a nitrogen atmosphere. Alkyne **S4** (1.93 g, 5.40 mol, 1 equiv.) and 3,5 bis(trifluoromethyl)phenylazide **S2** (1.38 g, 5.40 mol, 1 equiv.) were added to the reaction and stirred for 24 hrs. The reaction was then diluted with DCM (50 mL) and the organic phase was washed with 10 % aqueous EDTA/$NH_4OH$ solution (2 x 20 mL) and brine (20 mL), then dried over $MgSO_4$ and concentrated *in vacuo*. The crude product was purified by silica flash column chromatography in DCM: Hexane 1:1 to yield a white powder **1a** (2.03 g, 3.31 mmol, 61 %). $^1$H NMR (600 MHz, DMSO-$d_6$) δ 11.03 (s, 1H), 9.78 (s, 1H), 8.72 (d, *J* = 1.6 Hz, 2H), 8.60 (t, *J* = 1.8 Hz, 1H), 8.57 (d, *J* = 1.6 Hz, 2H), 8.32 (s, 1H), 8.21 (dt, *J* = 7.7, 1.4 Hz, 1H), 8.06 (dt, *J* = 7.9, 1.4 Hz, 1H), 7.85 (s, 1H), 7.76 (t, *J* = 7.7 Hz, 1H). $^{13}$C NMR (151 MHz, DMSO-$d_6$) δ 165.9, 147.0, 144.0, 141.0, 137.8, 134.9, 132.0, 131.8, 130.8, 130.6, 130.3, 129.5, 128.9, 127.7, 125.5, 124.7, 124.2, 123.7, 122.4, 121.9, 121.0, 120.7 (2C), 120.0 (2C). $^{19}$F NMR (565 MHz, DMSO-$d_6$) δ -61.34, -61.60. HRMS-ESI (m/z) Calculated for $C_{25}H_{13}F_{12}N_4O$ $[M+H]^+$, 613.0892 ; found 613.0905.

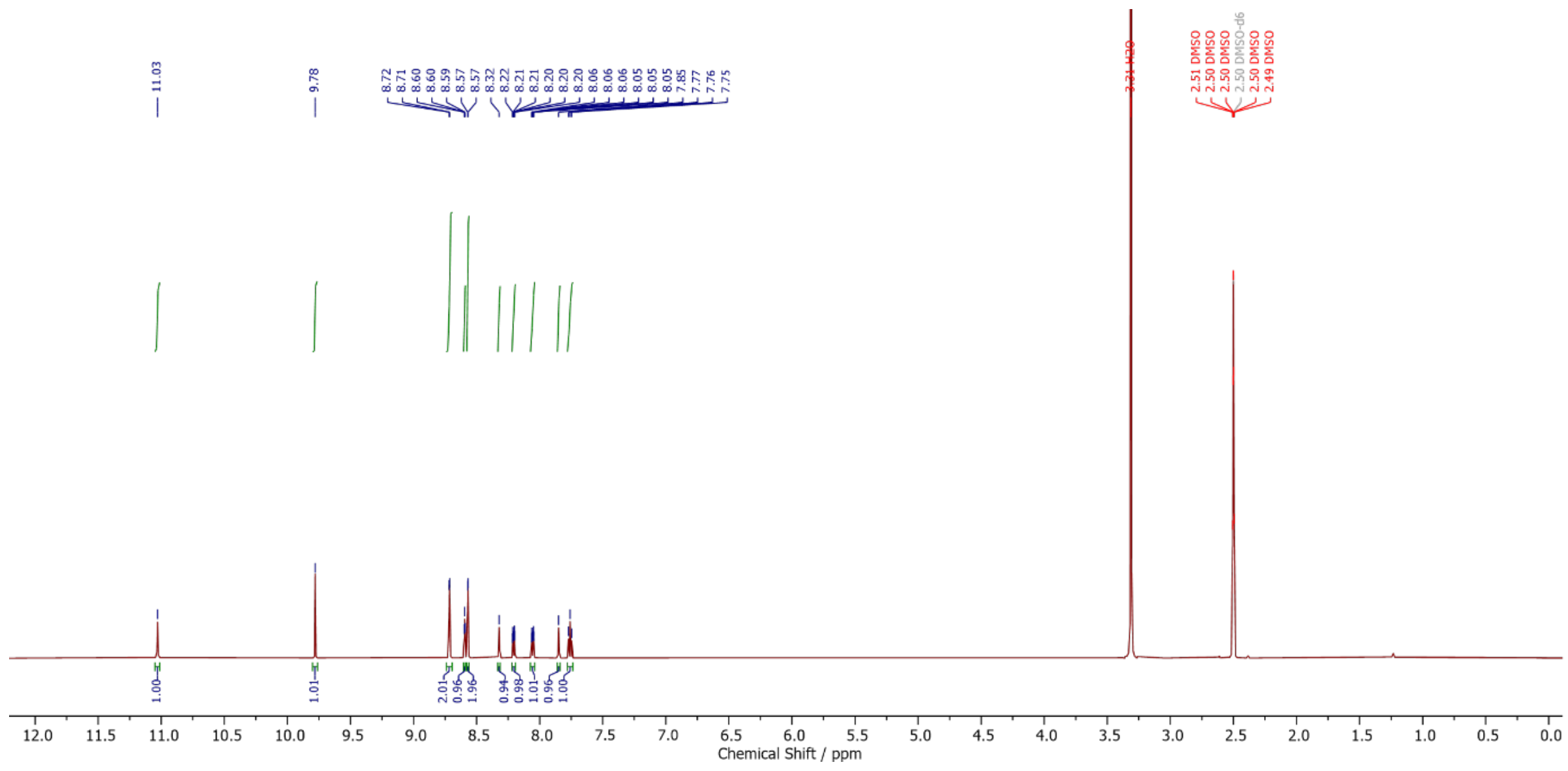

**Figure SI-3**: $^{1}$H NMR Spectrum of **1a** (DMSO-$d_6$, 298 K, 600 MHz)

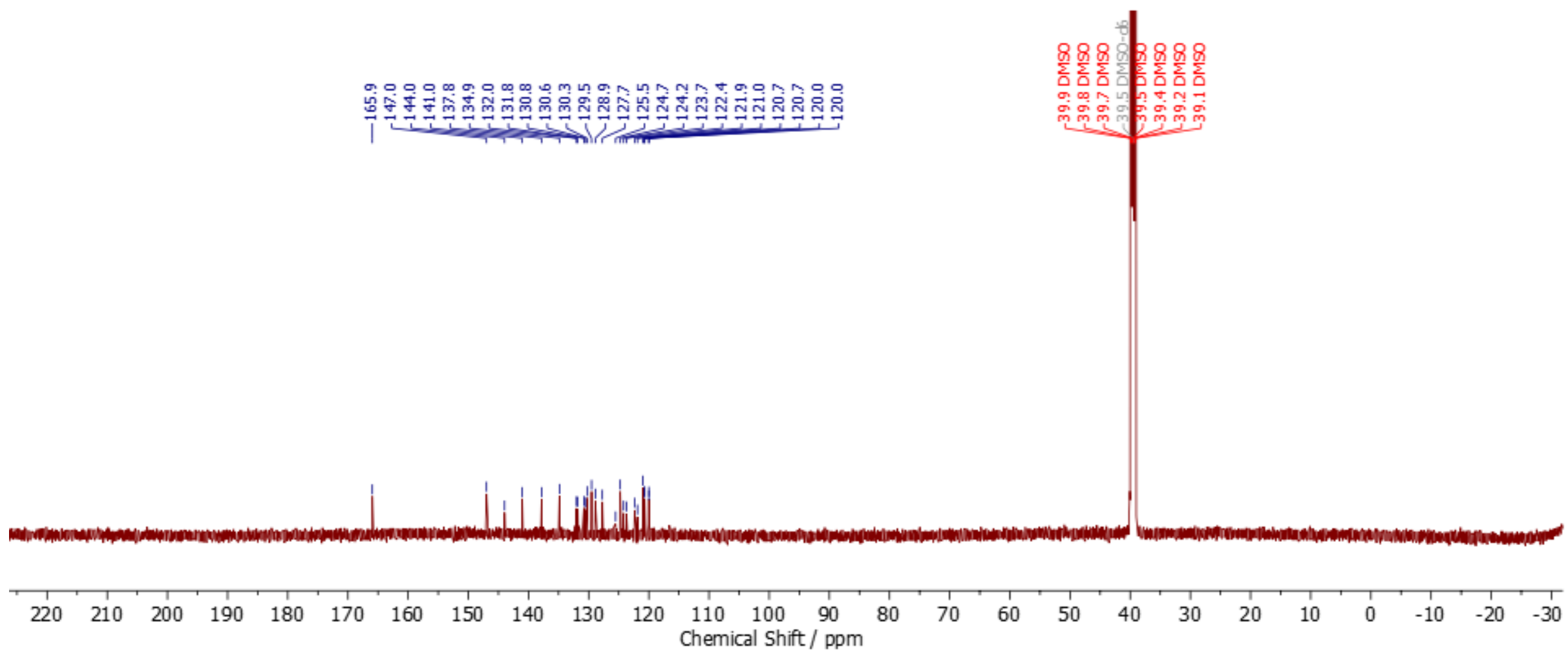

**Figure SI-4**: $^{13}$C NMR Spectrum of **1a** (DMSO-$d_6$, 298 K, 151 MHz)

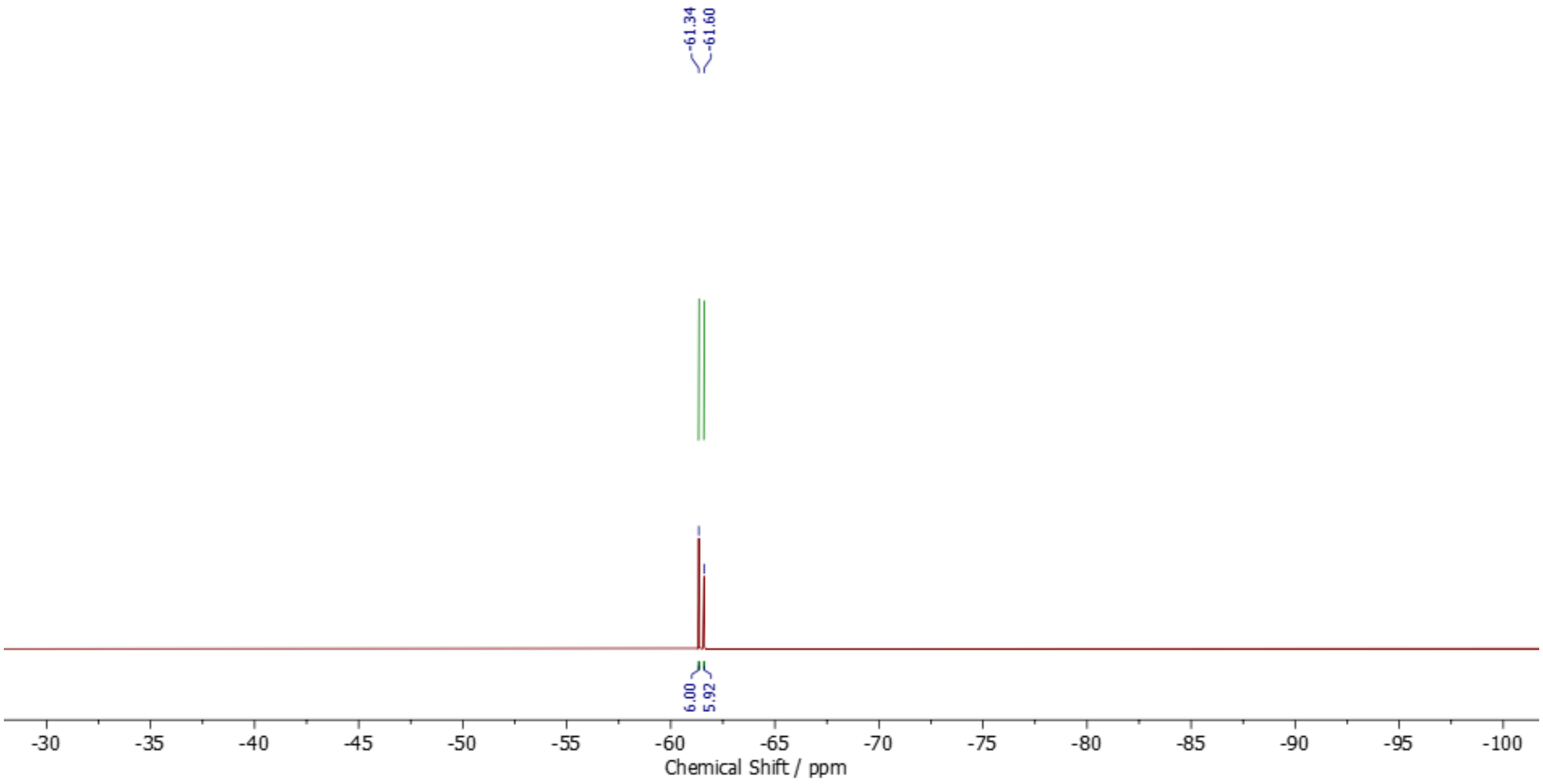


**Figure SI-5**: $^{19}$F NMR Spectrum of **1a** (DMSO-$d_6$, 298 K, 565 MHz)

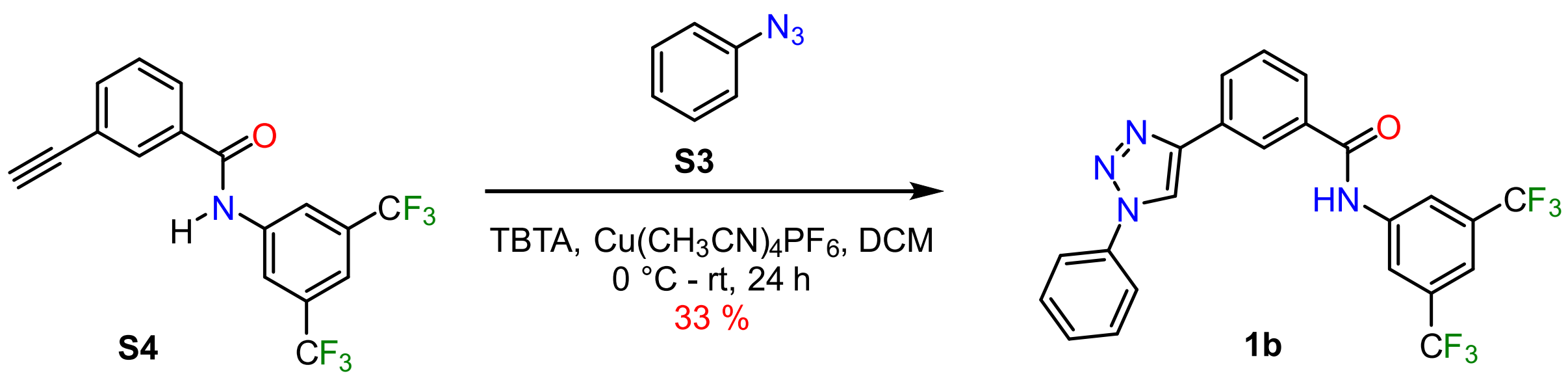


**Scheme 6:** *N*-(3,5-bis(trifluoromethyl)phenyl)-3-(1-phenyl-1*H*-1,2,3-triazol-4-yl)benzamide (Anionophore) **1b**

General click reaction procedure was followed. $Cu(MeCN)_4PF_6$ (10.5 mg, 0.03 mmol, 0.5 equiv.), TBTA (7.4 mg, 0.01 mmol, 0.25 equiv.) were stirred for 30 minutes in DCM (5 mL) under a nitrogen atmosphere. Alkyne **S4** (20 mg, 0.056 mmol, 1 equiv.) and phenylazide **S3** (8.7 mg, 0.07 mmol, 1.3 equiv.) were reacted. The reaction was then diluted with DCM (50 mL) and the organic phase was washed with 10 % aqueous EDTA/$NH_4OH$ solution (2 x 20 mL) and brine (20 mL), then dried over $MgSO_4$ and concentrated *in vacuo*. The crude product was purified by silica flash column chromatography in Hexane: Acetone 3:1 to yield a white powder **1b** (8.9 mg, 0.02 mmol, 33 %). $^1$H NMR (600 MHz, DMSO-$d_6$) δ 11.01 (s, 1H), 9.45 (s, 1H), 8.61 (t, *J* = 1.8 Hz, 1H), 8.57 (s, 2H), 8.20 (ddd, *J* = 7.7, 1.8, 1.1 Hz, 1H), 8.02 (ddd, *J* = 7.7, 1.9, 1.1 Hz, 1H), 8.01 – 7.97 (m, 2H), 7.86 – 7.83 (m, 1H), 7.73 (t, *J* = 7.7 Hz, 1H), 7.68 – 7.63 (m, 2H), 7.56 – 7.52 (m, 1H). $^{13}$C NMR (151 MHz, DMSO-$d_6$) δ 206.4, 166.0, 146.6, 141.1, 136.6, 134.8, 130.7 (q), 130.7, 130.0, 129.4, 128.9, 128.9, 127.5, 126.3 (q), 124.7, 120.3, 120.0 (3C), 118.1, 116.6, 116.5 (2C), 30.7. $^{19}$F NMR (565 MHz, DMSO-$d_6$) δ -61.48. HRMS-ESI (m/z) Calculated for $C_{23}H_{15}F_6N_4O$ $[M+H]^+$,477.1145; found 477.1124.

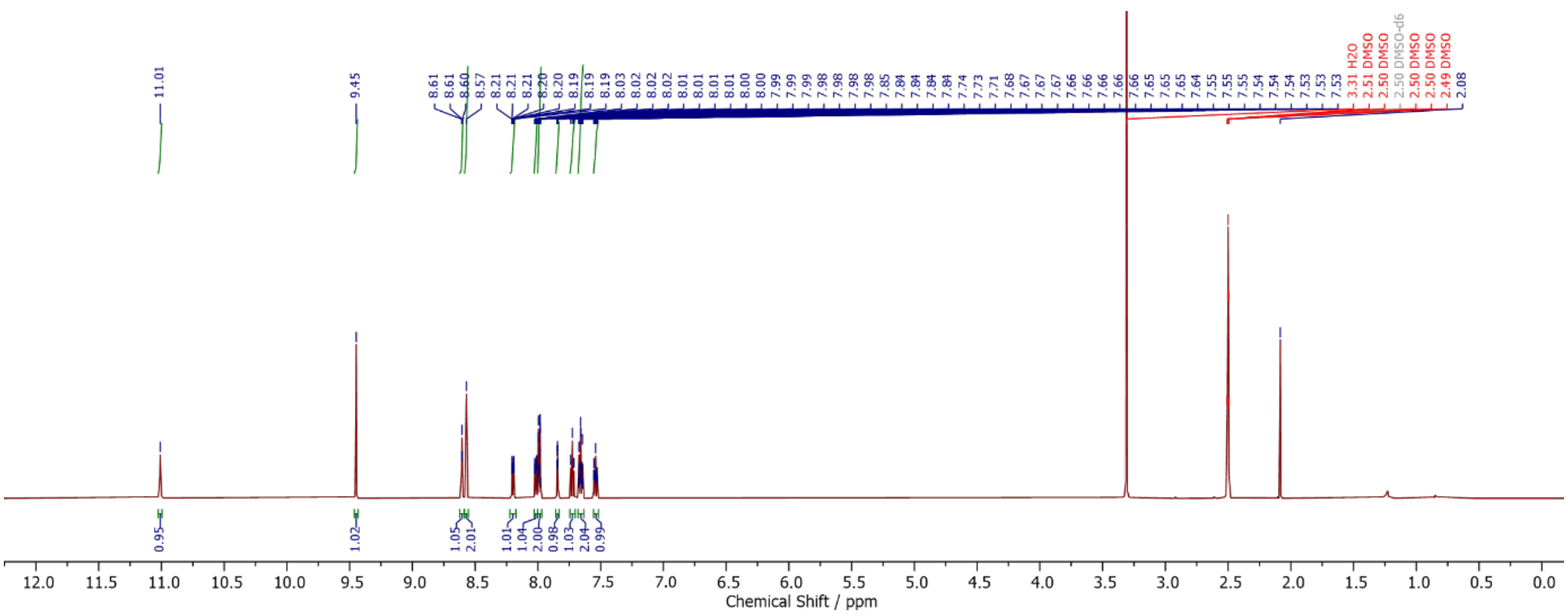


**Figure SI-6**: $^1$H NMR Spectrum of **1b** (DMSO-$d_6$, 298 K, 600 MHz)

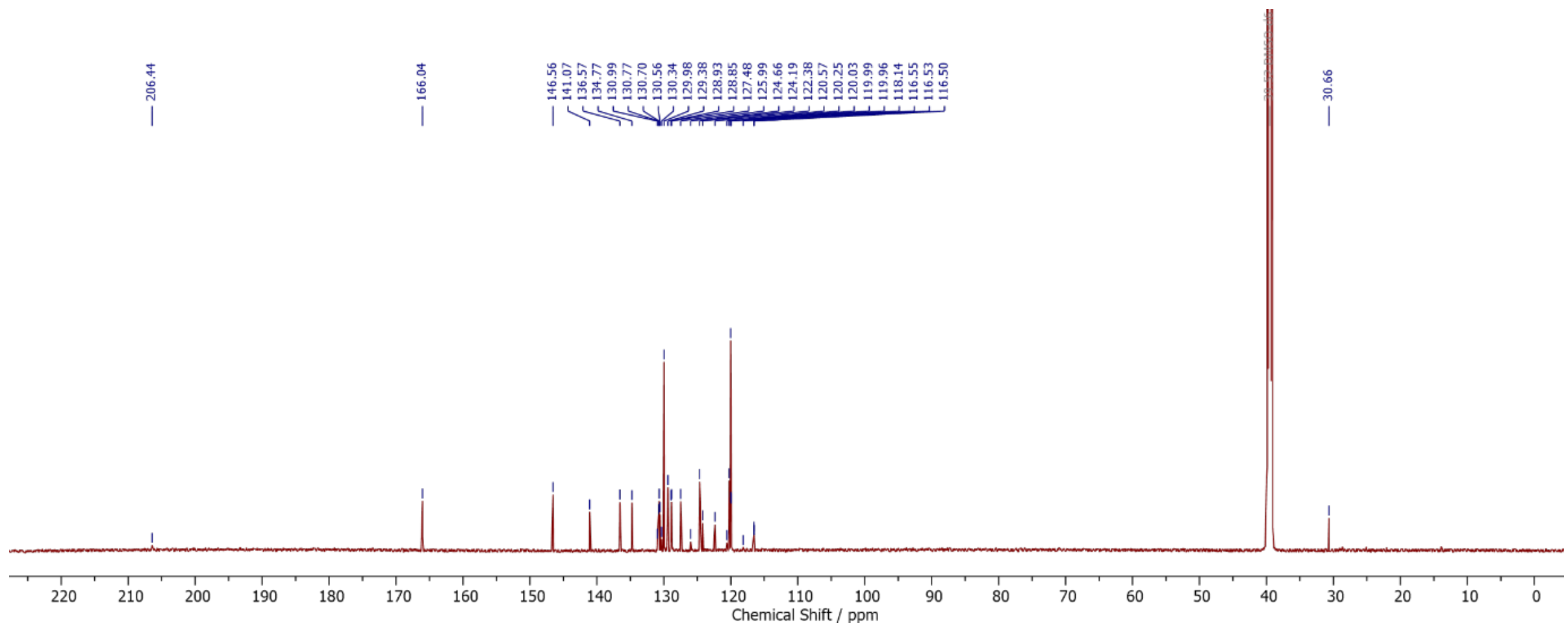

**Figure SI-7**: $^{13}$C NMR Spectrum of **1b** (DMSO-$d_6$, 298 K, 151 MHz)

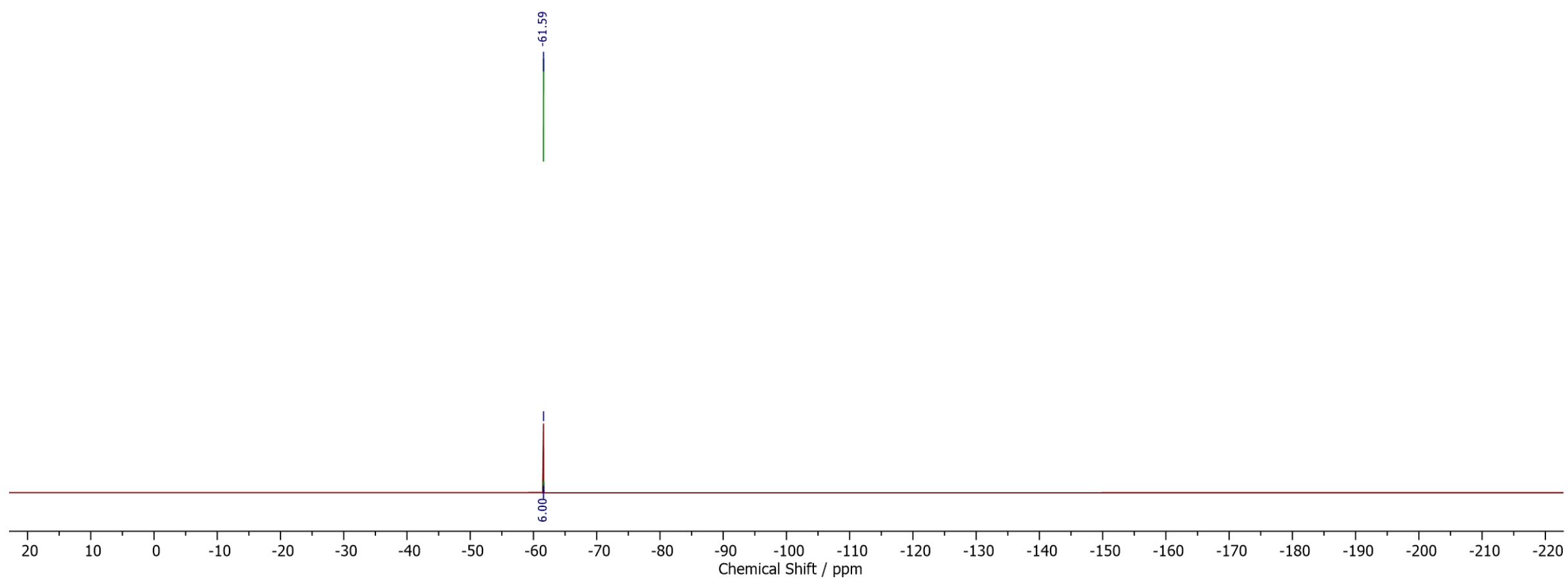

**Figure SI-8**: $^{19}$F NMR Spectrum of **1b** (DMSO-$d_6$, 298 K, 565 MHz)

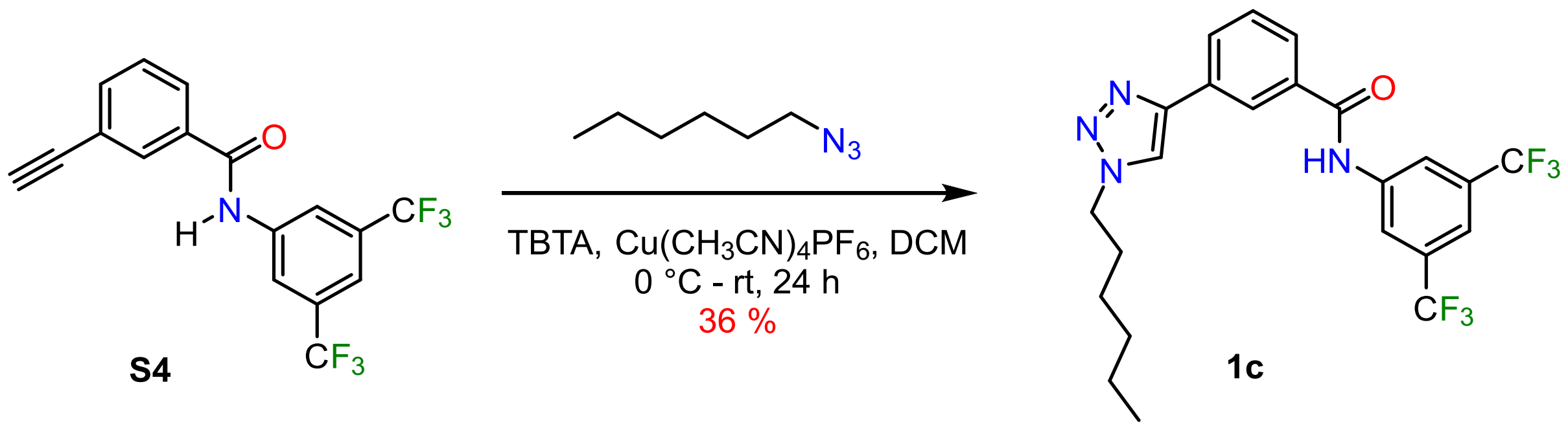


**Scheme 7:** *N*-(3,5-bis(trifluoromethyl)phenyl)-3-(1-hexyl-1*H*-1,2,3-triazol-4-yl)benzamide (Anionophore) **1c**

General click reaction procedure was followed. $Cu(MeCN)_4PF_6$ (10.0 mg, 0.027 mmol, 0.5 equiv.), TBTA (8.5 mg, 0.016 mmol, 0.25 equiv.) were stirred for 30 minutes in DCM (5 mL) under a nitrogen atmosphere. Alkyne **S4** (13.0 mg, 0.036 mmol, 1.0 equiv.) and hexyl azide (43.0 mg, 0.34 mmol) were reacted. The crude product was purified by silica flash column chromatography in Hexane: EtOAc 4:1 to yield a white powder **1c** (6.4 mg, 0.01 mmol, 36 %). $^{1}$H NMR (600 MHz, DMSO-$d_6$) δ 10.15 (s, 1H), 7.90 (s, 1H), 7.76 – 7.71 (m, 2H), 7.68 (t, *J* = 1.9 Hz, 1H), 7.28 (dt, *J* = 7.7, 1.5 Hz, 1H), 7.14 (dt, *J* = 7.7, 1.5 Hz, 1H), 7.02 (s, 1H), 6.85 (t, *J* = 7.8 Hz, 1H), 3.64 – 3.59 (m, 3H), 3.55 (d, *J* = 11.4 Hz, 2H), 1.07 (t, *J* = 7.2 Hz, 2H), 0.47 (tt, *J* = 6.2, 2.3 Hz, 6H). $^{13}$C NMR (151 MHz, DMSO-$d_6$) δ 166.1, 145.5, 141.1, 134.6, 131.3, 130.7 (q), 129.3, 128.7, 127.1, 126.3 (q), 124.3, 121.8, 120.0, 116.5, 72.5, 63.1, 49.6, 48.6, 30.6, 29.5, 25.5, 21.9, 13.8. $^{19}$F NMR (565 MHz, DMSO-$d_6$) δ -61.59. HRMS-ESI (m/z) Calculated for $C_{23}H_{22}F_6N_4ONa$ $[M+Na]^+$,507.1590; found 507.1597.

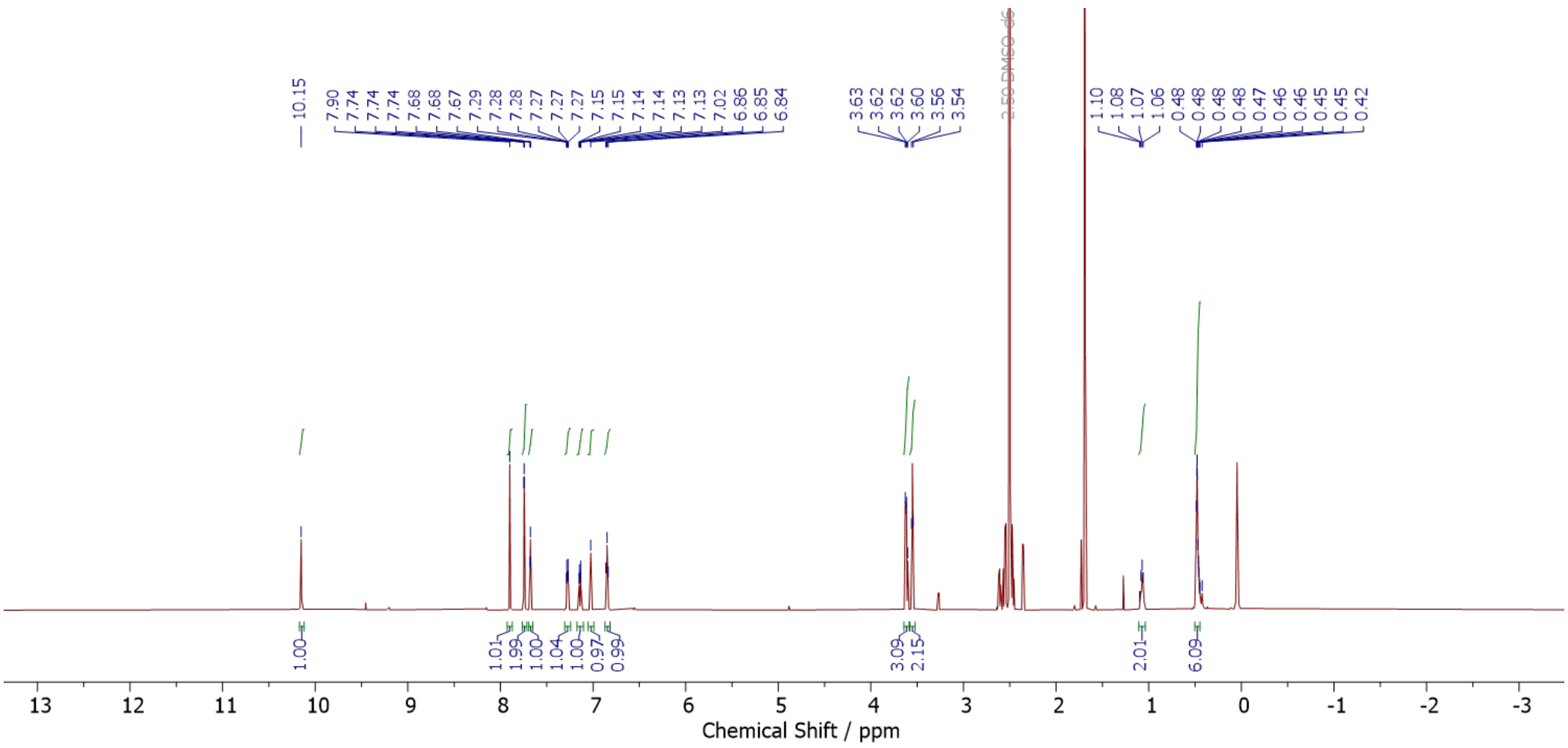


**Figure SI-9**: $^{1}$H NMR Spectrum of **1c** (DMSO-$d_6$, 298 K, 600 MHz)

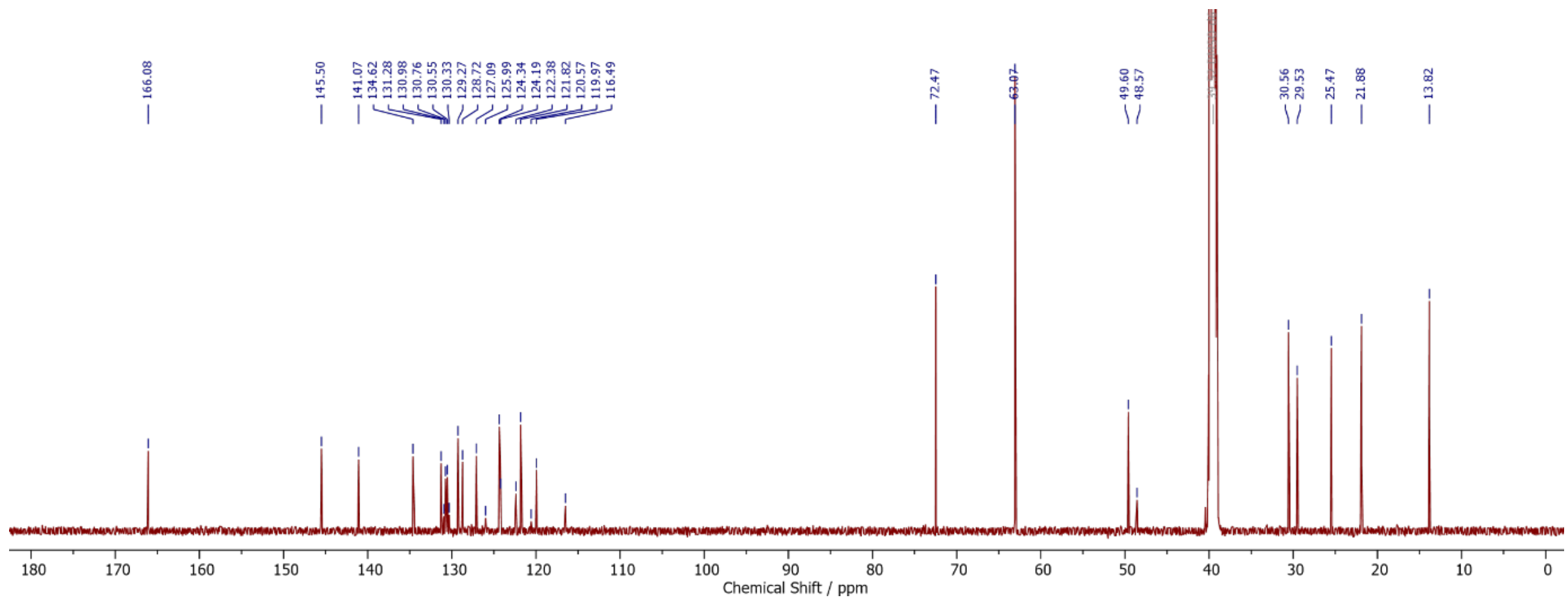


**Figure SI-10**: $^{13}C$ NMR Spectrum of **1c** (DMSO-$d_6$, 298 K, 151 MHz)

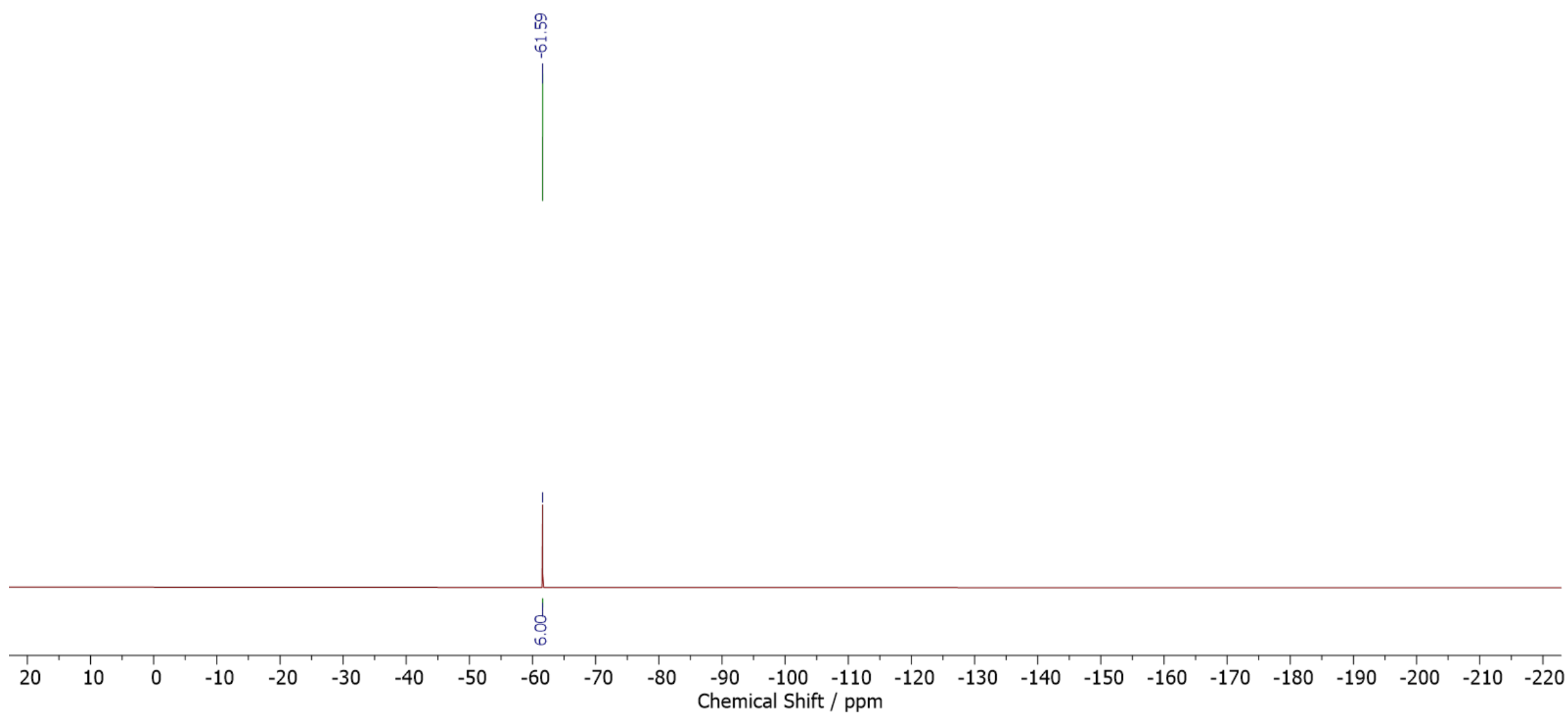


**Figure SI-11**: $^{19}F$ NMR Spectrum of **1c** (DMSO-$d_6$, 298 K, 565 MHz)

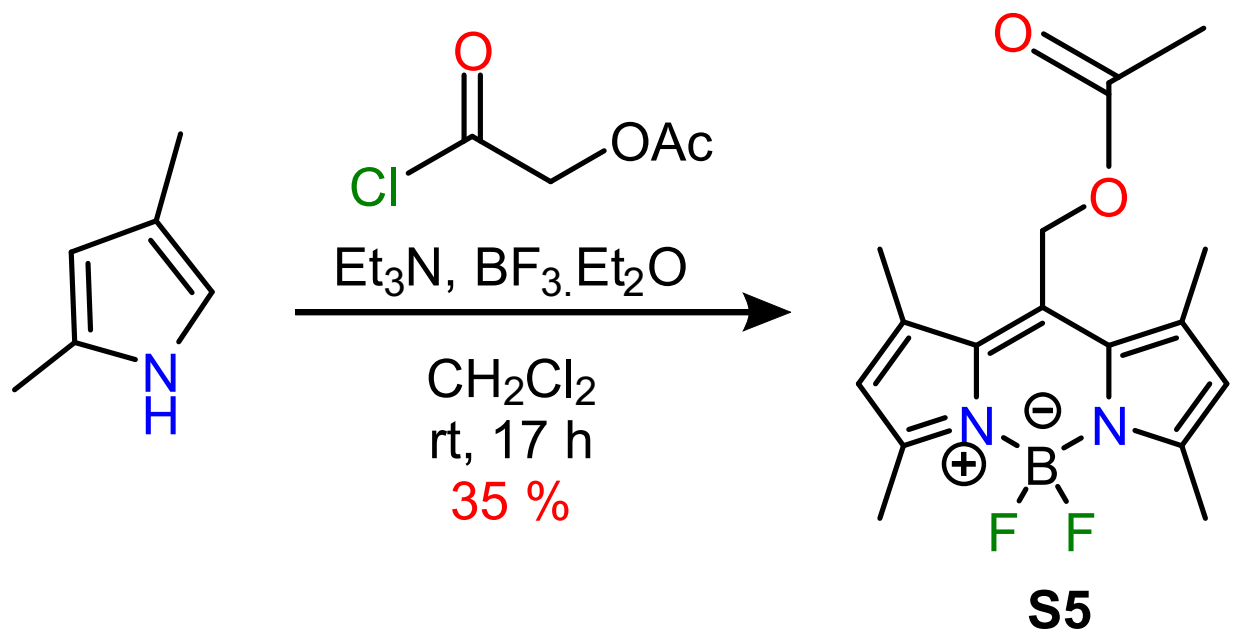


**Scheme 8:** Synthesis of 8-Acetoxymethyl-1,3,5,7-tetramethyl pyrromethene fluoroborate **S5**

Prepared by a literature procedure.[3] 2-Chloro-2-oxoethyl acetate (1.7 mL, 16 mmol, 1.2 equiv.) was added to a solution of 2,4-dimethylpyrrole (2.6 g, 31 mmol, 2 equiv.) in dry DCM (125 mL) under a nitrogen atmosphere. The reaction mixture was stirred in the dark at rt for 17 hrs. After this time, the flask was opened and TEA (11 mL, 79 mmol, 6 equiv.) was added. The resulting mixture was allowed to stir for 15 min. Then, the flask was again put under a nitrogen atmosphere, and boron trifluoride diethyl etherate (14.6 mL, 118 mmol, 9 equiv.) was added. After 1 hr, another portion of TEA (11 mL, 79 mmol, 6 equiv.) and boron trifluoride diethyl etherate (14.6 mL, 118 mmol, 9 equiv.) was added. Then, silica (100 g) was added to the flask, and the solvents were evaporated. The crude was purified by silica gel flash-column chromatography in pentane: $Et_2O$ 2:1. The product **S5** was obtained as red-gold crystals (1.5 g, 4.7 mmol, 35 % yield). $^1H$ NMR (400 MHz, $CDCl_3$) δ 6.09 (s, 2H), 5.30 (s, 2H), 2.53 (s, 6H), 2.36 (s, 6H), 2.13 (s, 3H). Data consistent with that given in the literature.[3, 4]

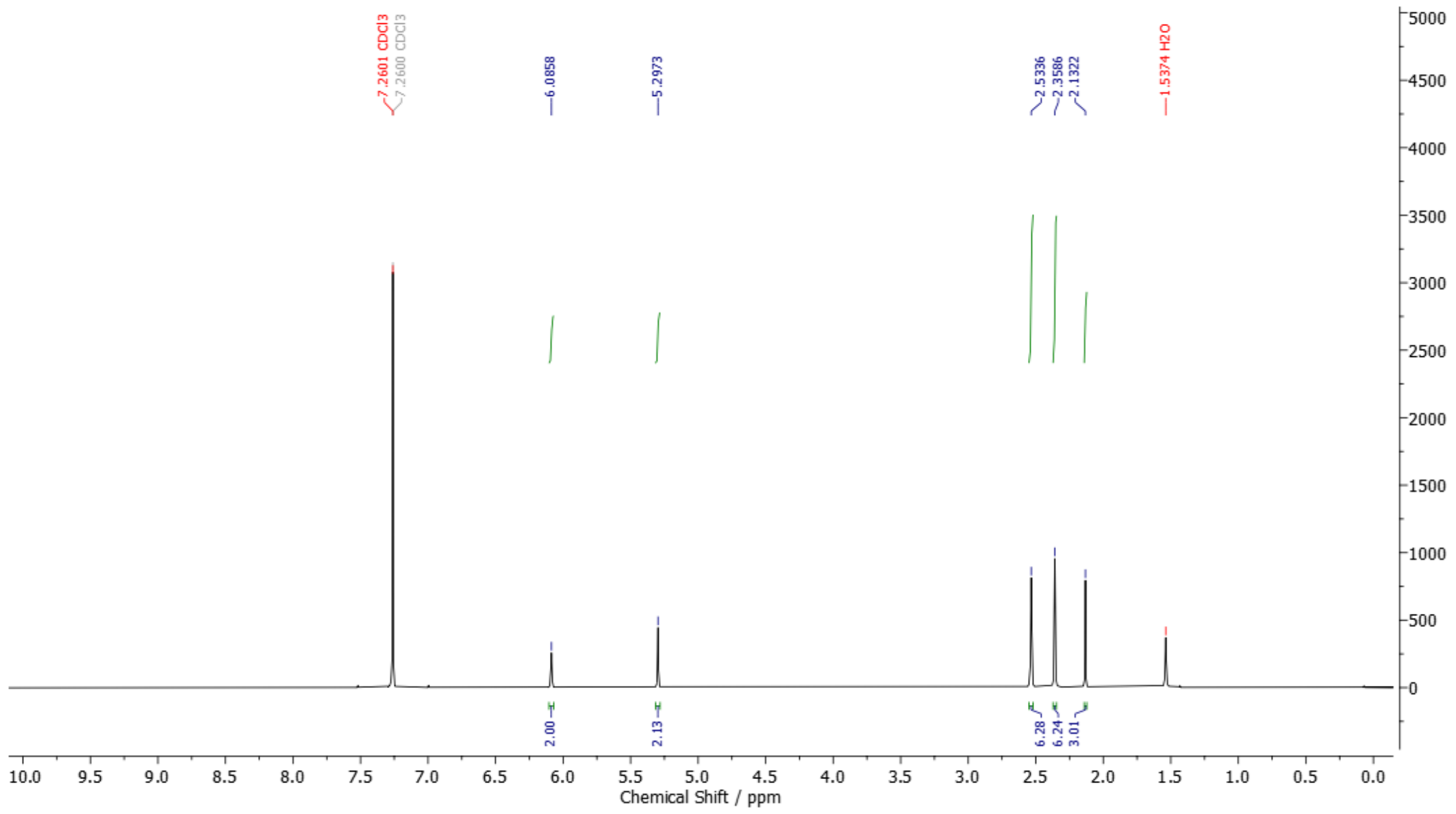


**Figure SI-12**: $^1H$ NMR Spectrum of **S5** ($CDCl_3$, 298 K, 400 MHz)

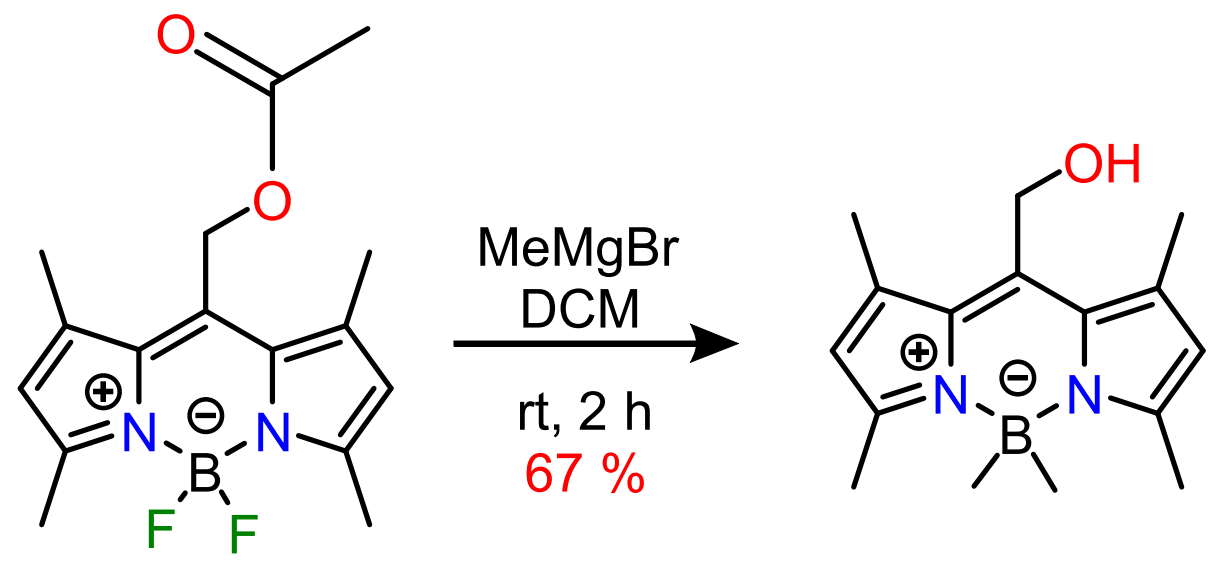


**Scheme 9:** Synthesis of 8-Hydroxymethyl-1,3,5,7-tetramethyl pyrromethene methylborate **S6**

Prepared by a literature procedure.[5] Methylmagnesium bromide in THF (1.1 mL, 9.4 mmol, 15 equiv.) was added to a solution of 8-Acetoxymethyl-1,3,5,7-tetramethyl pyrromethene fluoroborate **S5** (200 mg, 0.62 mmol, 1 equiv.) in dry DCM (10 mL) under a nitrogen atmosphere. The reaction mixture was stirred in the dark at rt for 1 hr, after which the reaction was complete by TLC. The reaction was quenched with $NH_4Cl$ (100 mL). The organic layer was extracted with EtOAc (50 mL) and washed with $NH_4Cl$ (60 mL) and with brine (2 x 60 mL), then dried over $MgSO_4$, filtered and concentrated *in vacuo*. Compound **S6** was purified by silica gel flash-column chromatography using DCM as the eluent. The product was obtained as a dark pink solid (114 mg, 0.42 mmol, 67 % yield). $^1$H NMR (400 MHz, $CDCl_3$) δ 6.09 (s, 2H), 4.96 (s, 2H), 2.52 (d, 6H), 2.46 (d, 6H), 1.56 (s, 1H), 0.18 (s, 6H). Data is consistent with that given in the literature.[4-6]

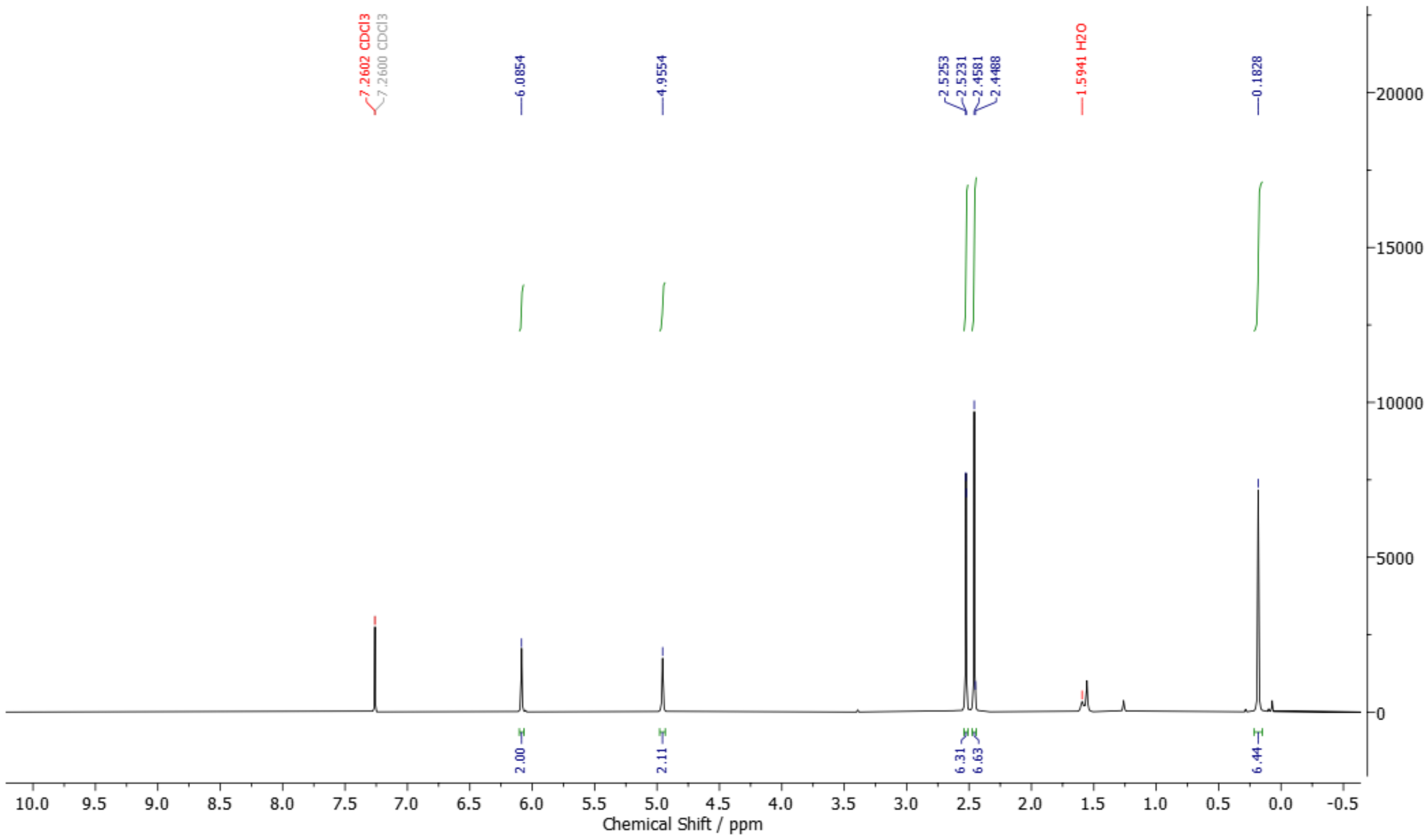


**Figure SI-13**: $^1$H NMR Spectrum of **S6** ($CDCl_3$, 298 K, 400 MHz)

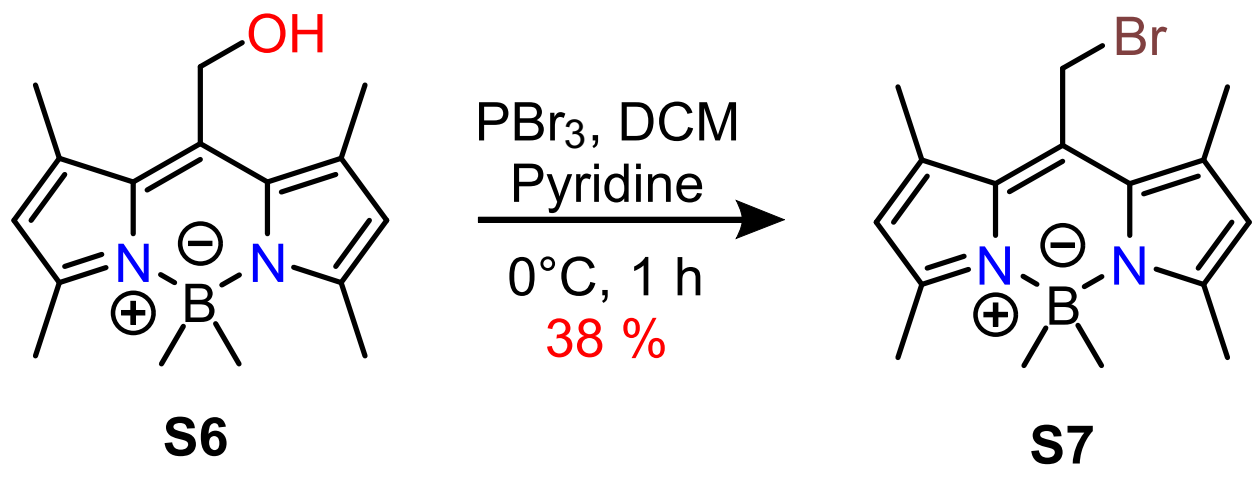


**Scheme 10:** Synthesis of 8-Bromomethyl-1,3,5,7-tetramethyl pyrromethene methylborate (BODIPY) **S7**

Prepared by a literature procedure.[5] Pyridine (66 µL, 0.81 mmol, 2 equiv.), followed by phosphorous tribromide ($PBr_3$) (55 µL, 0.61 mmol, 1.5 equiv.) was added to a solution of 8-hydroxymethyl-1,3,5,7-tetramethyl pyrromethene methylborate **S6** (110 mg, 0.41 mmol, 1 equiv.) in dry DCM (5.5 mL) at 0°C under a nitrogen atmosphere. The reaction mixture was stirred in the dark for 1 hr, after which the reaction was complete by TLC. Ice water (20 mL) was added to the reaction mixture and the organic layer was extracted with DCM (10 mL). The organic layer was washed with saturated $Na_2CO_3$ (20 mL), saturated $NH_4Cl$ (20 mL), and brine (20 mL), and then dried over $MgSO_4$. The solvent was reduced under vacuum. Compound **S7** was purified by silica gel flash-column chromatography in hexane: DCM 9:1. The product was obtained as a reddish solid (51 mg, 0.15 mmol, 38 % yield). $^1$H NMR (400 MHz, $CDCl_3$) δ 6.09 (s, $2H_{2,6}$), 4.77 (s, $2H_9$), 2.58 (d, $J$ = 0.9 Hz, $6H_{11,12}$), 2.46 (d, $6H_{13,14}$), 0.21 (s, $3H_{15/16}$), 0.15 (s, $3H_{16/15}$). Data consistent with that given in the literature.[5]

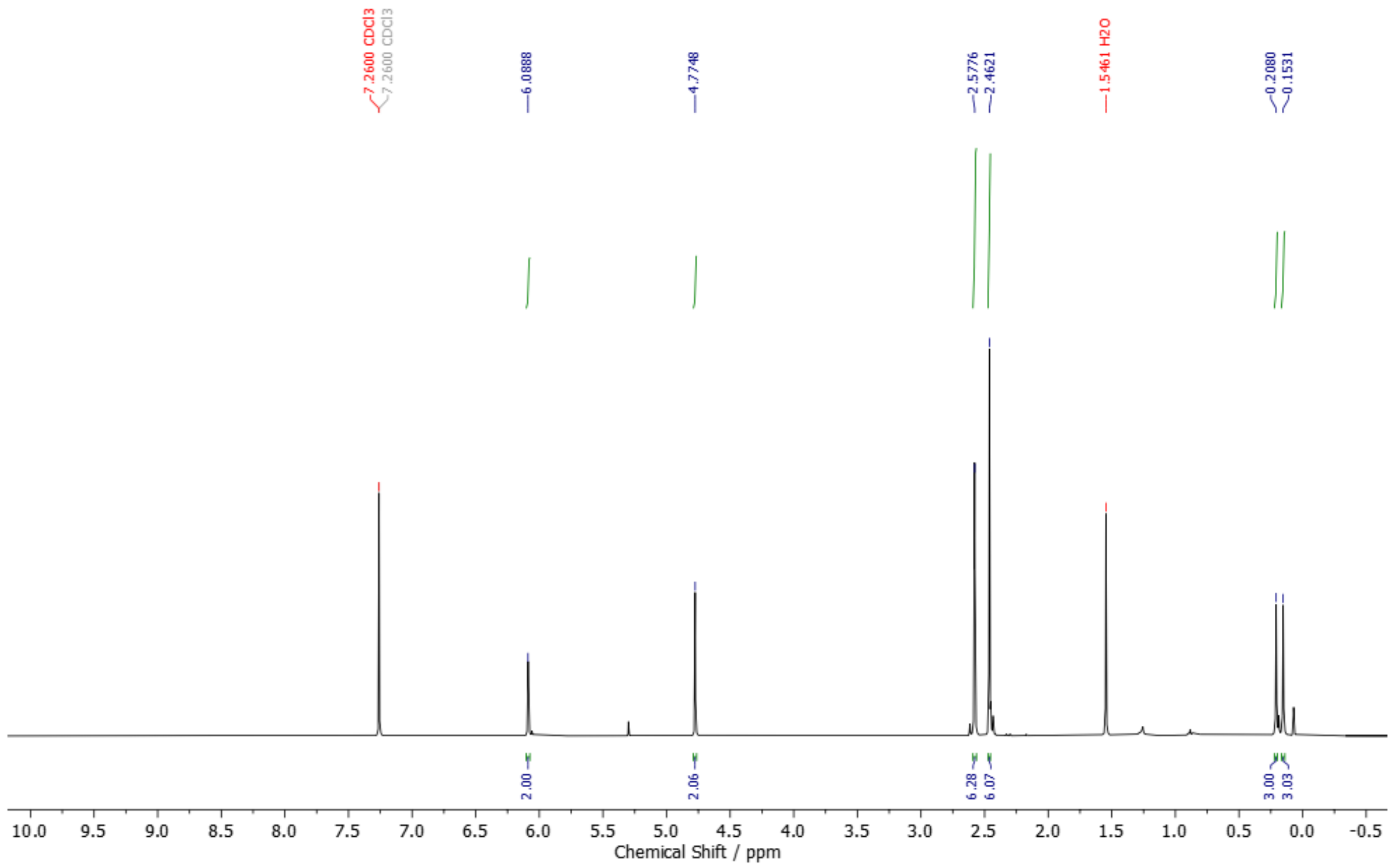


**Figure SI-14**: $^1$H NMR Spectrum of **S7** ($CDCl_3$, 298 K, 400 MHz)

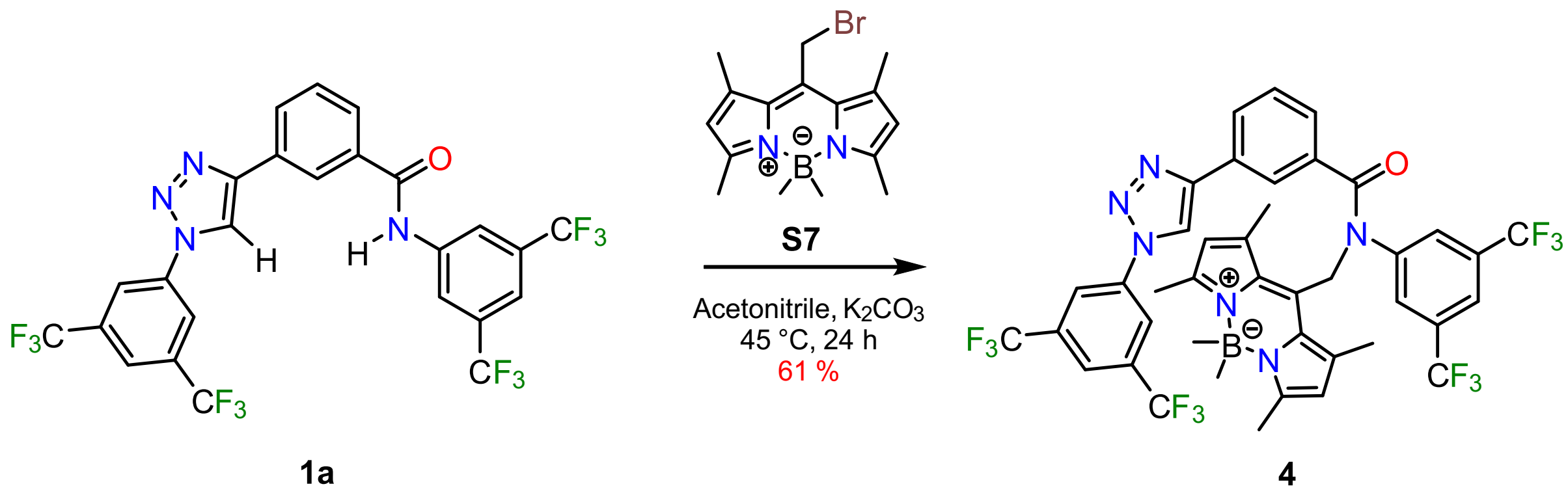

**Scheme 11:** Synthesis of BODIPY-photocaged amido-triazole **4**

Amido-triazole transporter **1a** (50.0 mg, 0.08 mmol, 1 equiv.) was combined with $K_2CO_3$ (33.9 mg, 0.24 mmol, 3 equiv.) in acetonitrile (10 mL) at 0˚C. After the reaction mixture returned to rt, BODIPY-photocage **S7** (40.8 mg, 0.12 mmol, 1.5 equiv.) was added and the reaction was left to stir for 24 hrs at 45˚C. The crude product was obtained by removing the solvent *in vacuo*, diluting the reaction with DCM (15 mL), washing with water (10 mL) and brine (10 mL) and drying over $MgSO_4$. After concentrating under reduced pressure, the crude oil was purified by silica gel flash column chromatography in Hexane: EtOAc 3:1 to yield product **4** as a reddish solid (42.8 mg, 0.05 mmol, 61 %). $^{1}$H NMR (600 MHz, $CDCl_3$) δ 8.27 (s, 2H), 8.16 (s, 1H), 7.97 (s, 1H), 7.86 (d, J = 6.2 Hz, 1H), 7.79 (s, 1H), 7.48 (s, 1H), 7.32 (t, J = 7.7 Hz, 1H), 7.27 (s, 1H), 7.19 (s, 2H), 6.07 (s, 2H), 5.46 (s, 2H), 2.53 (s, 6H), 2.35 (s, 6H), 0.09 (s, 3H), -0.30 (s, 3H). $^{13}$C NMR (151 MHz, $CDCl_3$) δ 170.3, 153.6, 148.1, 141.2, 137.9, 136.5, 135.7, 134.2, 134.0, 133.8, 133.5 (2C), 131.9, 131.7, 131.4, 131.2, 130.3, 129.8, 129.2, 128.9, 127.9, 126.1, 125.4, 122.6 (q), 123.5, 123.4, 122.5, 121.8, 121.6, 121.5 (2C), 120.5, 120.4 (3C), 119.8, 117.5, 45.0, 29.9, 16.9, 16.5. $^{19}$F NMR (565 MHz, $CDCl_3$) δ -63.00, -63.06. HRMS-ESI (m/z) Calculated for $C_{41}H_{33}BF_{12}N_6O$ $[M+H]^+$, 865.2690; found 865.2696.

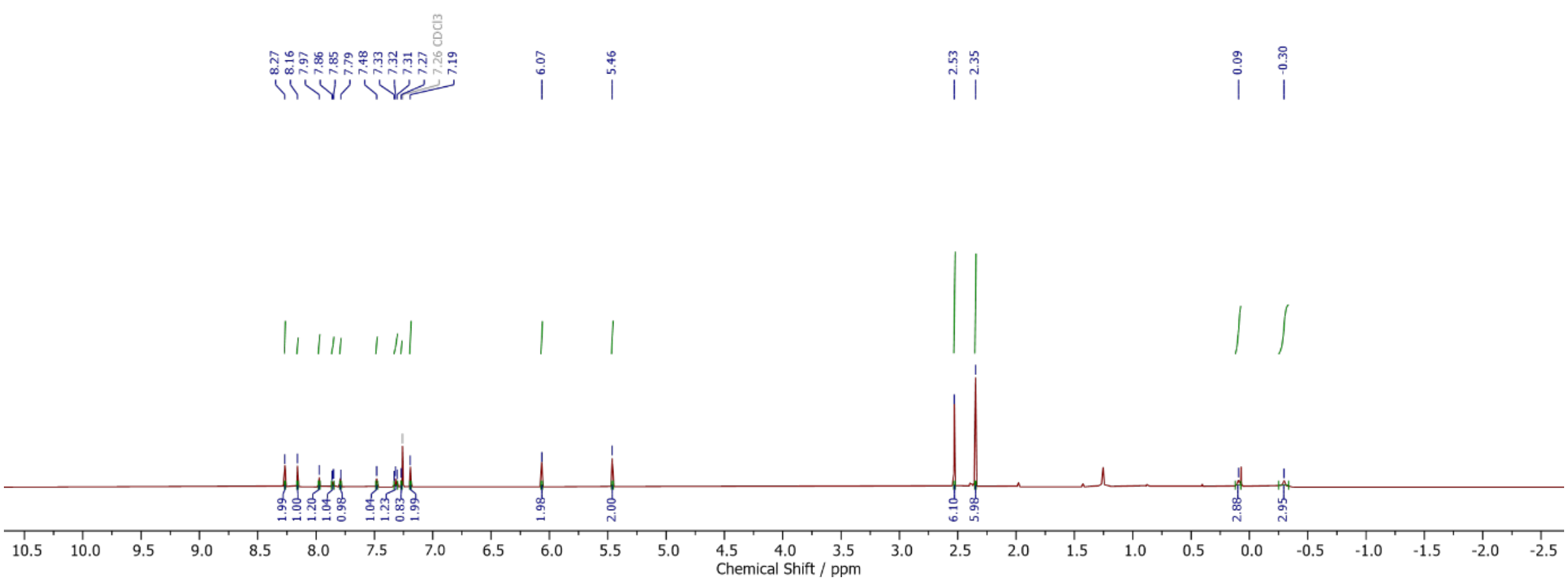

**Figure SI-15**: $^{1}$H NMR Spectrum of **4** ($CDCl_3$, 298 K, 600 MHz)

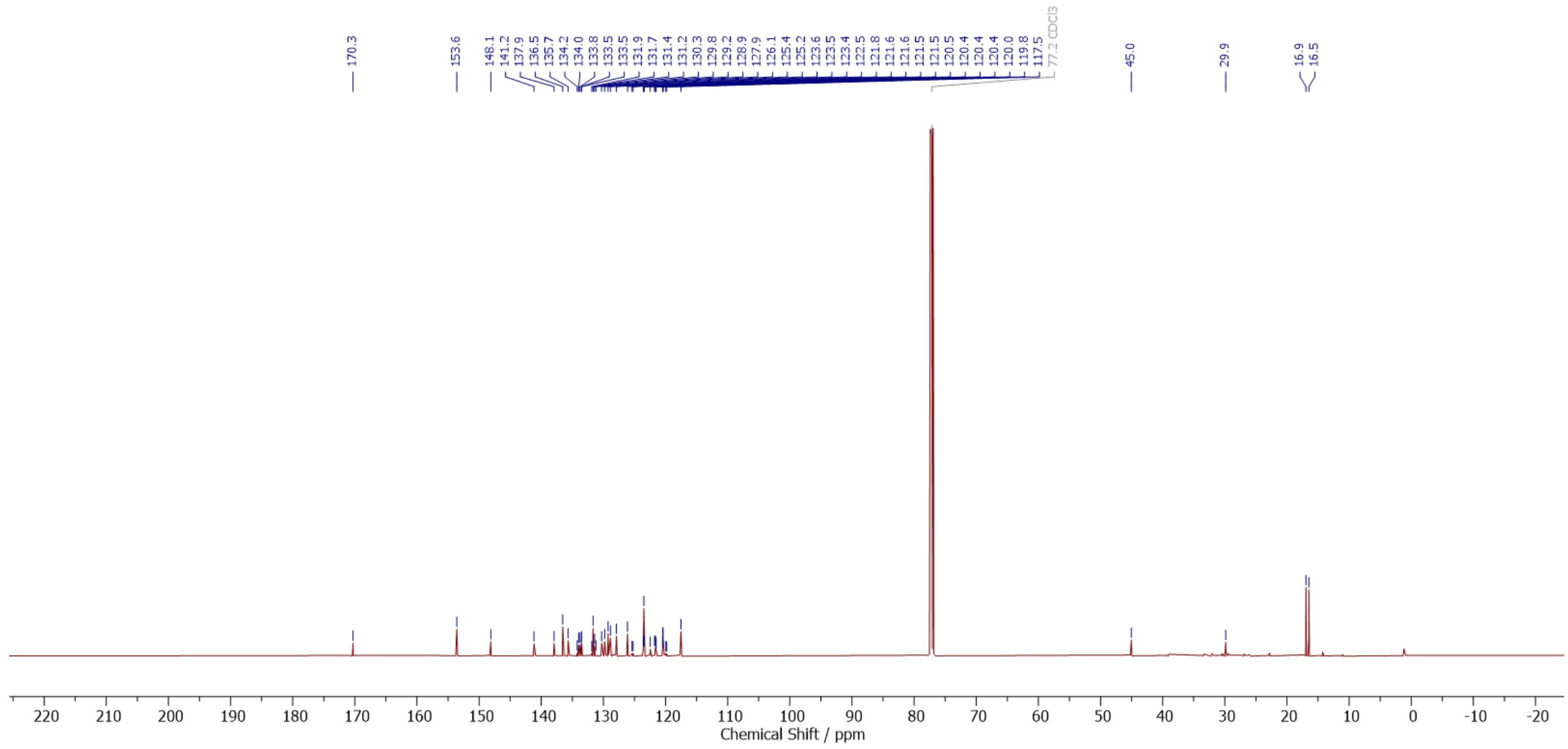

**Figure SI-16**: $^{13}C$ NMR Spectrum of **4** ($CDCl_3$, 298 K, 151 MHz)

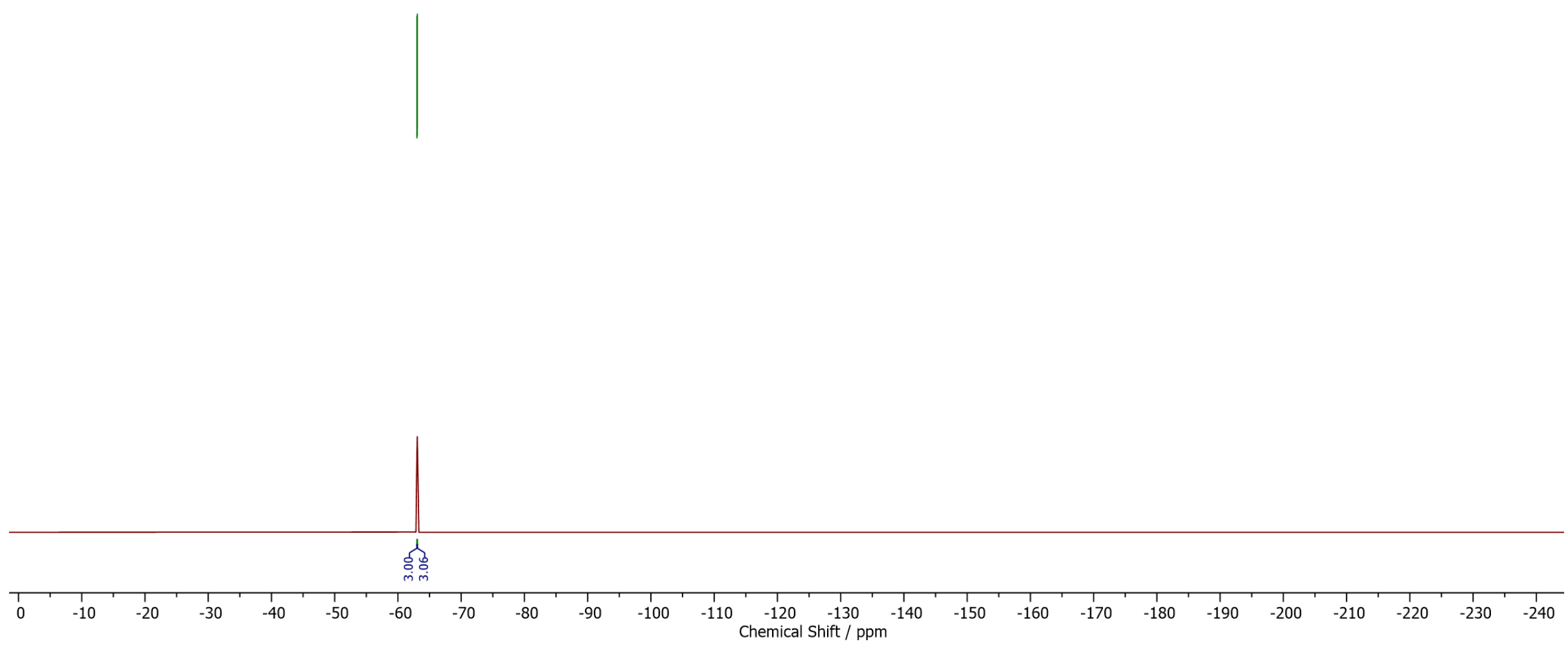

**Figure SI-17**: $^{19}F$ NMR Spectrum of **4** ($CDCl_3$, 298 K, 565 MHz)

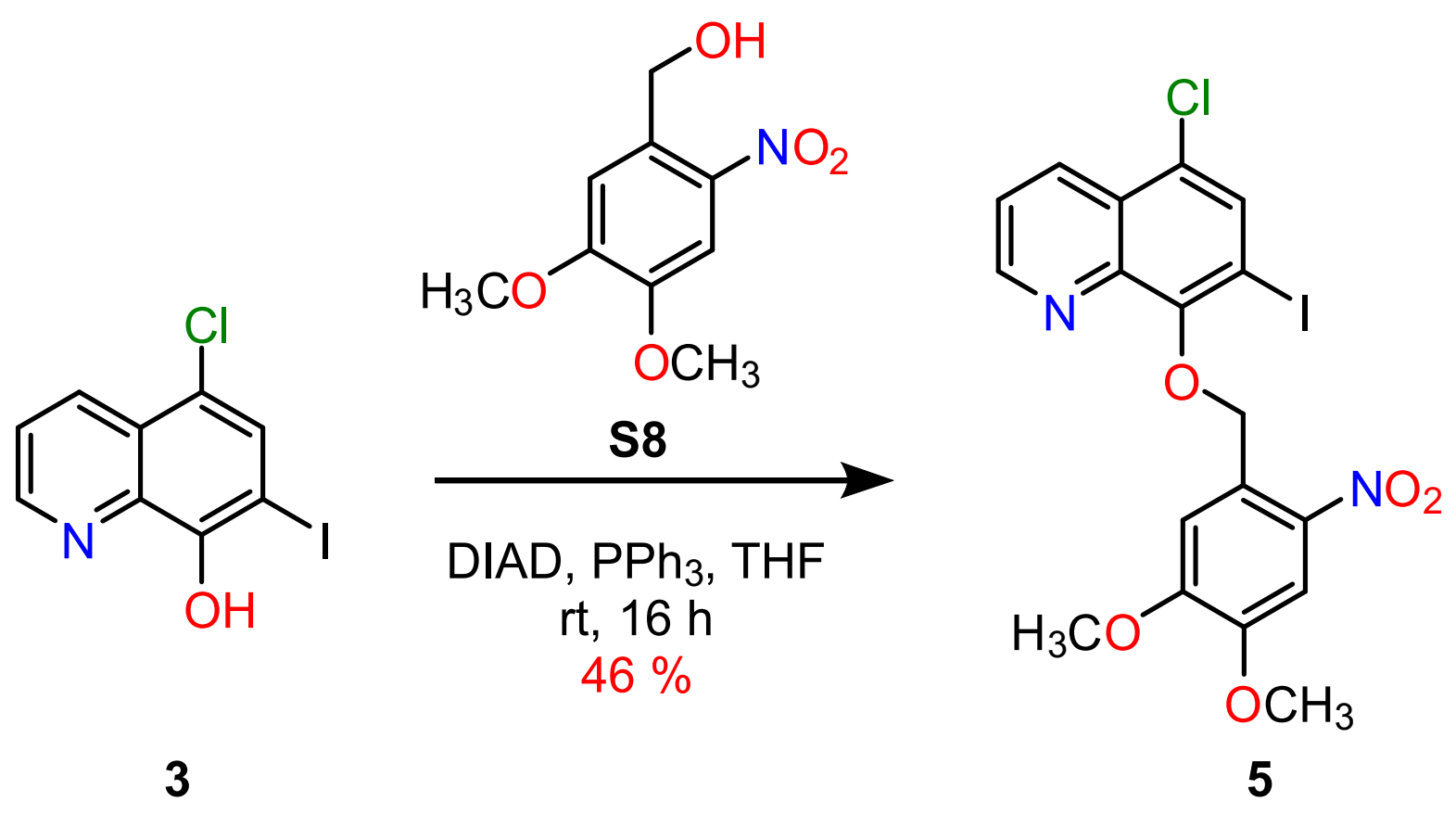


**Scheme 12:** Synthesis of DMNB-photocaged clioquinol **5**

Prepared by a modified literature procedure.[7] Clioquinol **3** (100 mg, 0.33 mmol, 1.0 equiv.), 4,5-dimethoxy-2-nitrobenzyl alcohol **S8** (140 mg, 0.65 mmol, 2.0 equiv.) and triphenylphosphine (172 mg, 0.65 mmol, 2.0 equiv.) were dissolved in anhydrous THF (3.0 mL). DIAD (56 µL, 0.36 mmol, 1.1 equiv.) was added to the solution and allowed to stir at room temperature for 16 hrs. The precipitate was filtered and washed with cold THF to give the product as an off white solid **5** (75 mg, 0.15 mmol, 46 %). $^1$H NMR (600 MHz, DMSO-$d_6$) δ 9.02 (dd, $J$ = 4.2, 1.6 Hz, 1H), 8.58 (dd, $J$ = 8.6, 1.6 Hz, 1H), 8.18 (s, 1H), 7.86 (s, 1H), 7.79 (dd, $J$ = 8.5, 4.1 Hz, 1H), 7.74 (s, 1H), 5.80 (s, 2H), 3.98 (s, 3H), 3.90 (s, 3H). $^{13}$C NMR (151 MHz, DMSO-$d_6$) δ 154.4, 153.4, 151.0, 147.7, 141.6, 138.9, 134.7, 133.3, 128.3, 126.7, 125.8, 123.3, 111.3, 107.9, 91.5, 72.3, 56.4, 56.1. HRMS-ESI (m/z) Calculated for $C_{18}H_{15}ClIN_2O_5$ $[M+H]^+$, 500.9709; found 500.9712.

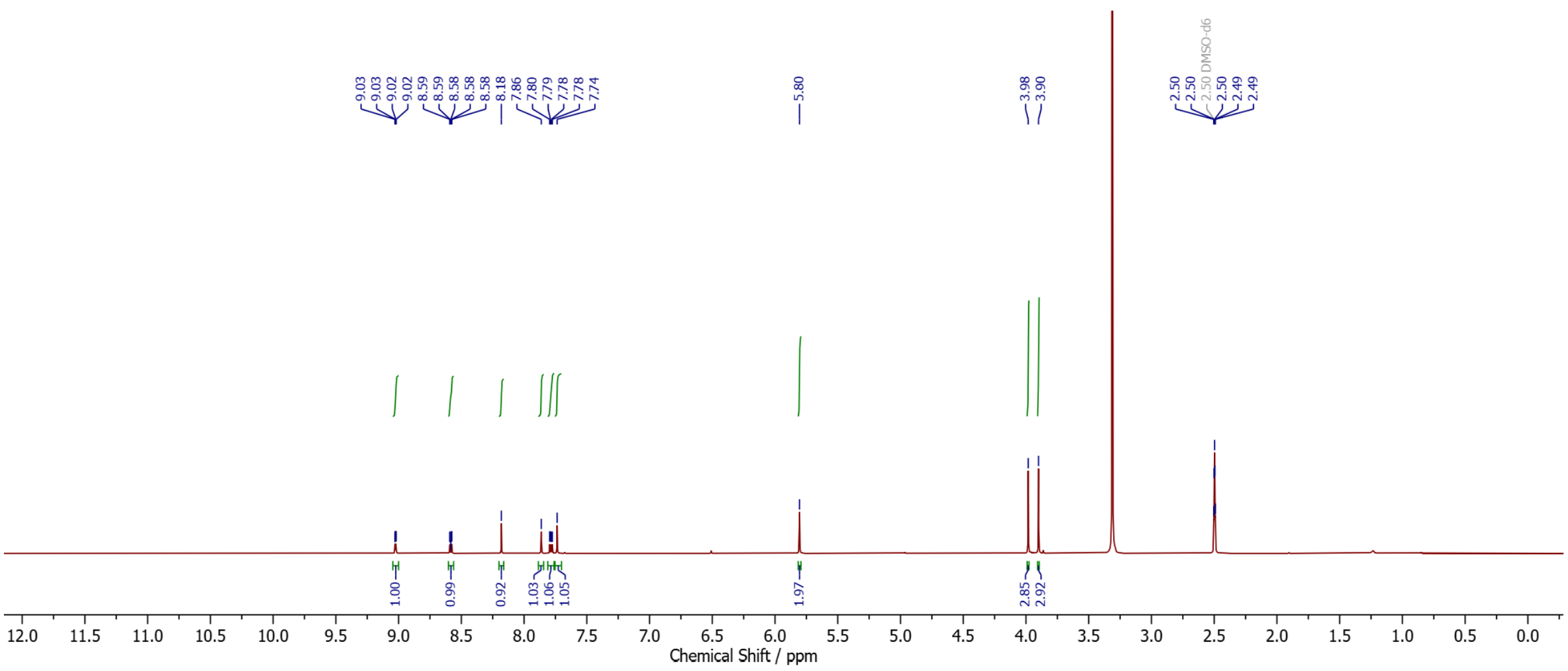


**Figure SI-18**: $^1$H NMR Spectrum of **5** (DMSO-$d_6$, 298 K, 600 MHz)

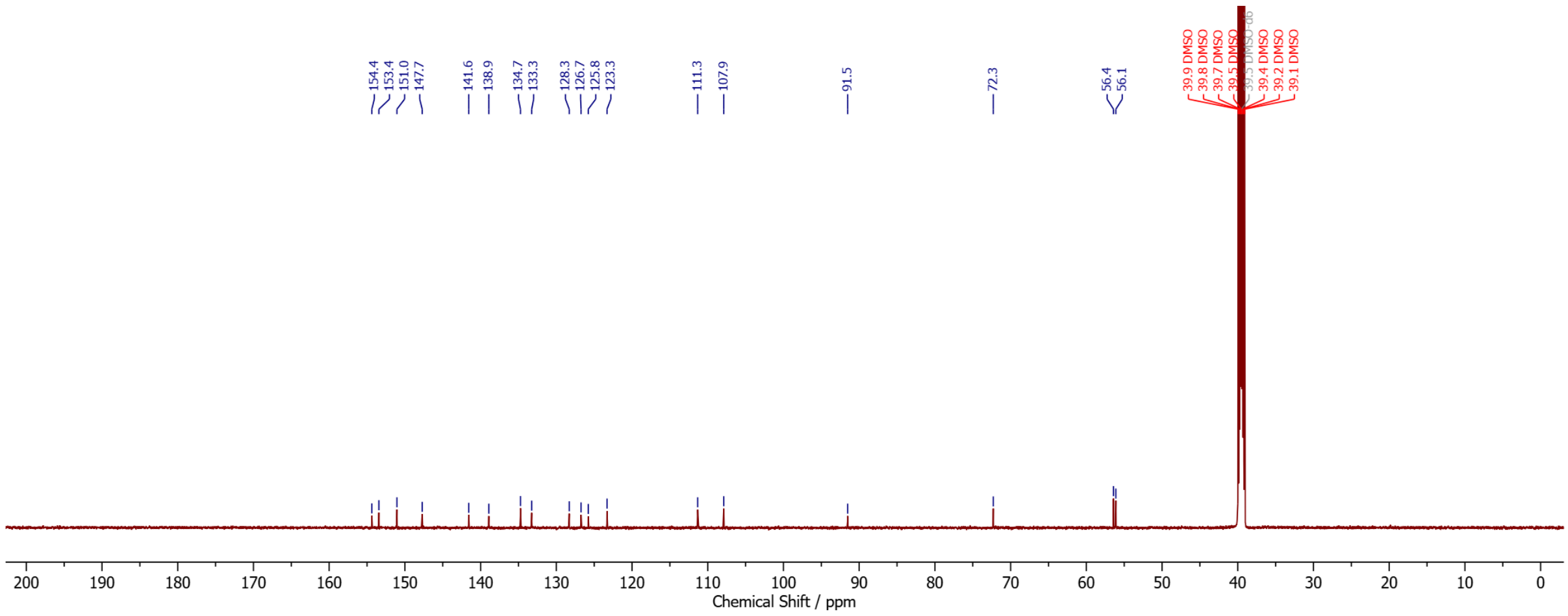


**Figure SI-19**: $^{13}$C NMR Spectrum of **5** (DMSO-$d_6$, 298 K, 151 MHz)

## 3 $^{1}$H NMR Titration Experiments

Chloride binding by amido-triazole transporters **1a-c** were assessed by $^{1}$H NMR titrations using a Bruker AVIII 500 spectrometer at 500 MHz and 298 K. The initial sample volume was 0.5 mL, at a concentration of 1 mM of host dissolved in MeCN-$d_3$. Known volumes of the anion guest, as the tetrabutylammonium (TBA) salt in the same solvent, were added to the host and the spectra were recorded after 0, 0.1, 0.2, 0.3, 0.4, 0.5, 1.0, 2.0, 3.0, 4.0, 6.0, 8.0, 10.0, 12.0, and 14.0 equivalents, the stacked partial $^{1}$H spectra shown in **Figure SI-20(ii)**. The chemical shift perturbations of the host spectra were monitored as a function of guest concentration. The spectra were referenced to the residual solvent peak. The data was analysed using a global fit procedure using the Bindfit program,[8, 9] using non-linear least squares analysis. The data were fitted to 1:1 host-guest binding isotherm, to afford the association constants in **SI-Table 1**. The association of guest and host was fast on the NMR timescale.

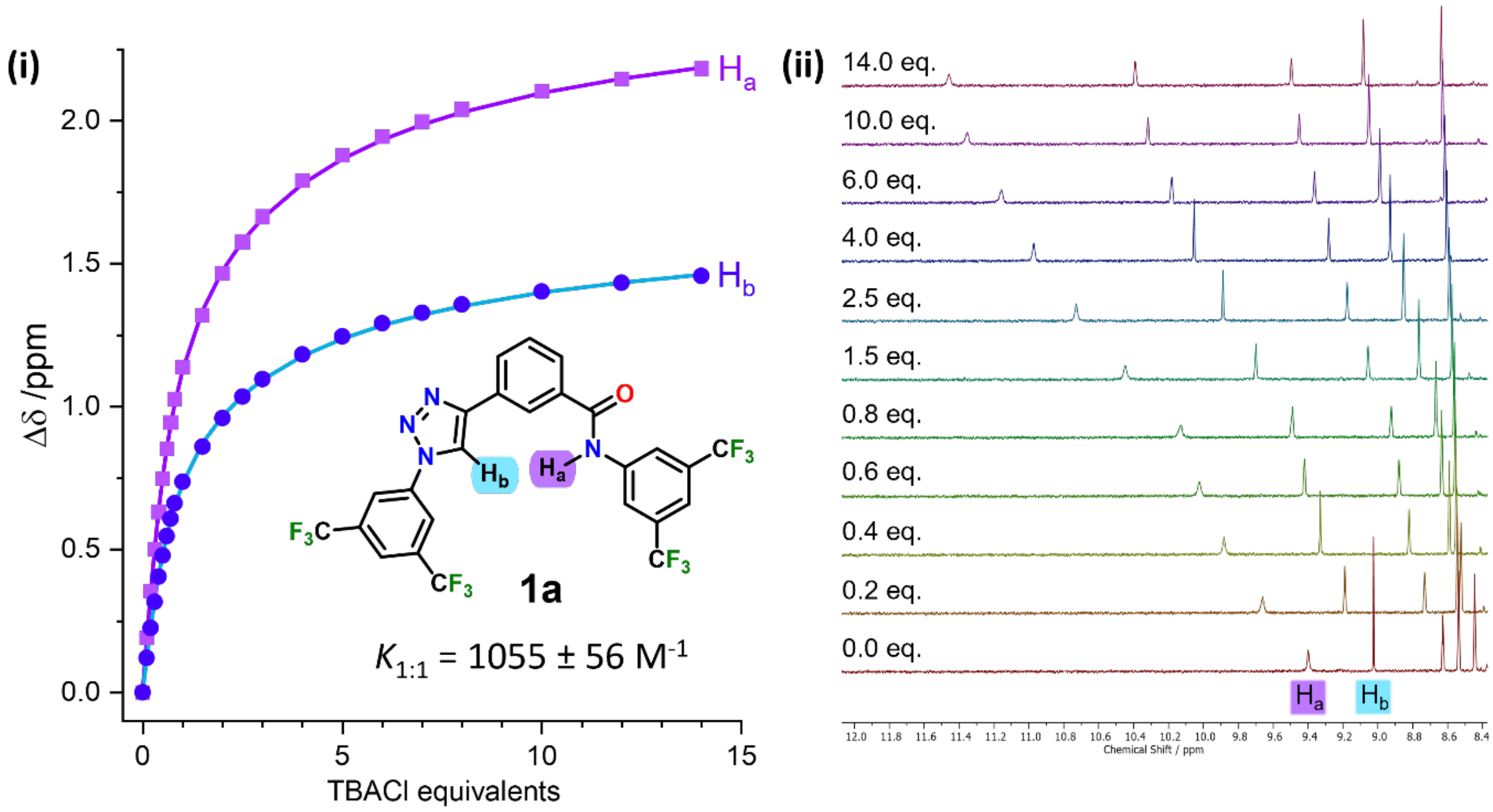


**Figure SI-20: (i)** Chloride binding isotherm for amido-triazole transporter **1a** in MeCN-$d_3$. Experimental data shown by symbols (■, ●), fitted 1:1 binding isotherm shown by solid lines (▬, ▬). **(ii)** Stacked partial $^{1}$H NMR Spectra of **1a** with increasing equivalents of TBACl.

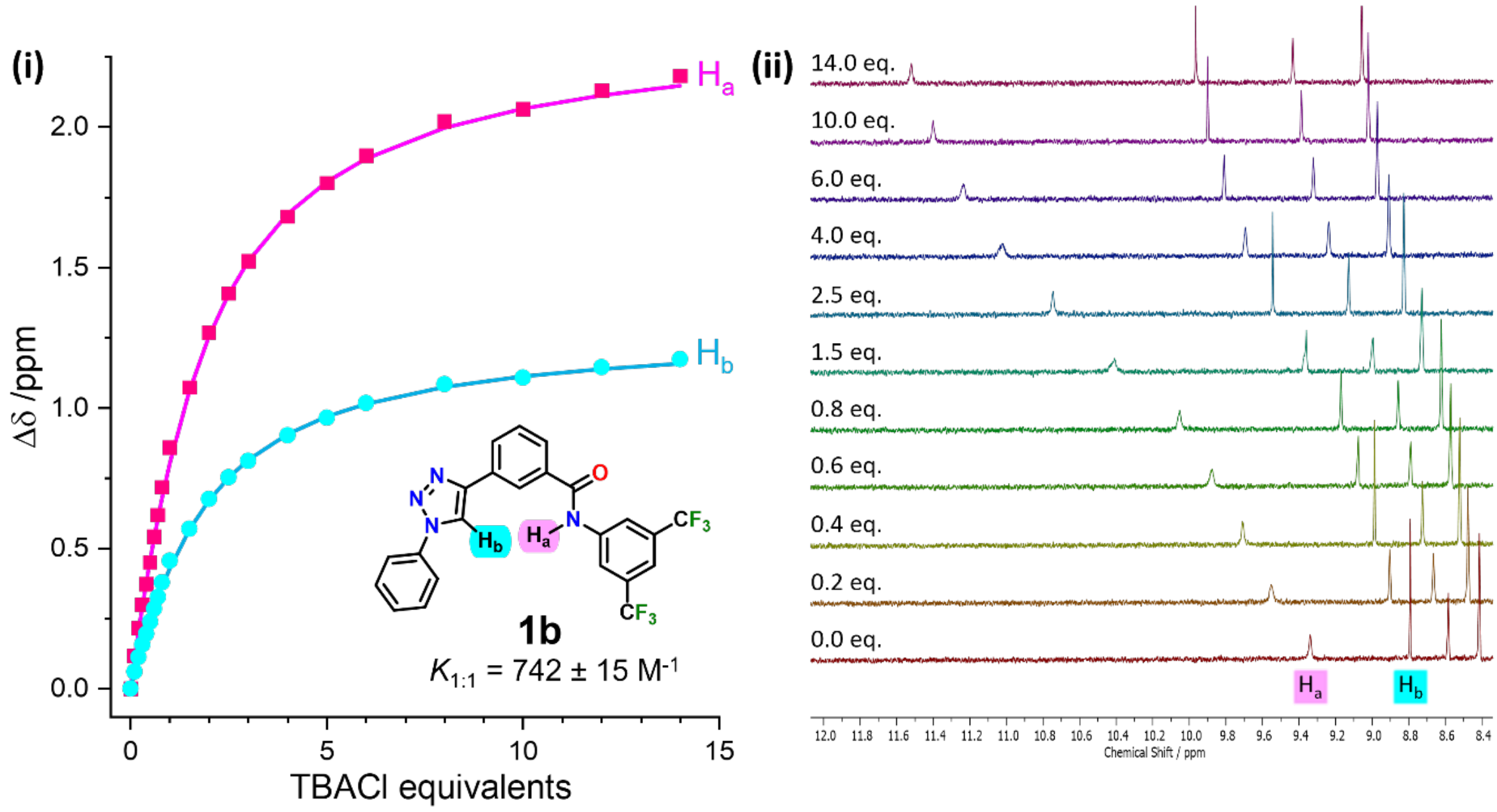


**Figure SI-21: (i)** Chloride binding isotherm for amido-triazole transporter **1b** in MeCN-$d_3$. Experimental data shown by symbols (■, ●), fitted 1:1 binding isotherm shown by solid lines (▬, ▬). **(ii)** Stacked partial $^1$H NMR Spectra of **1b** with increasing equivalents of TBACl.

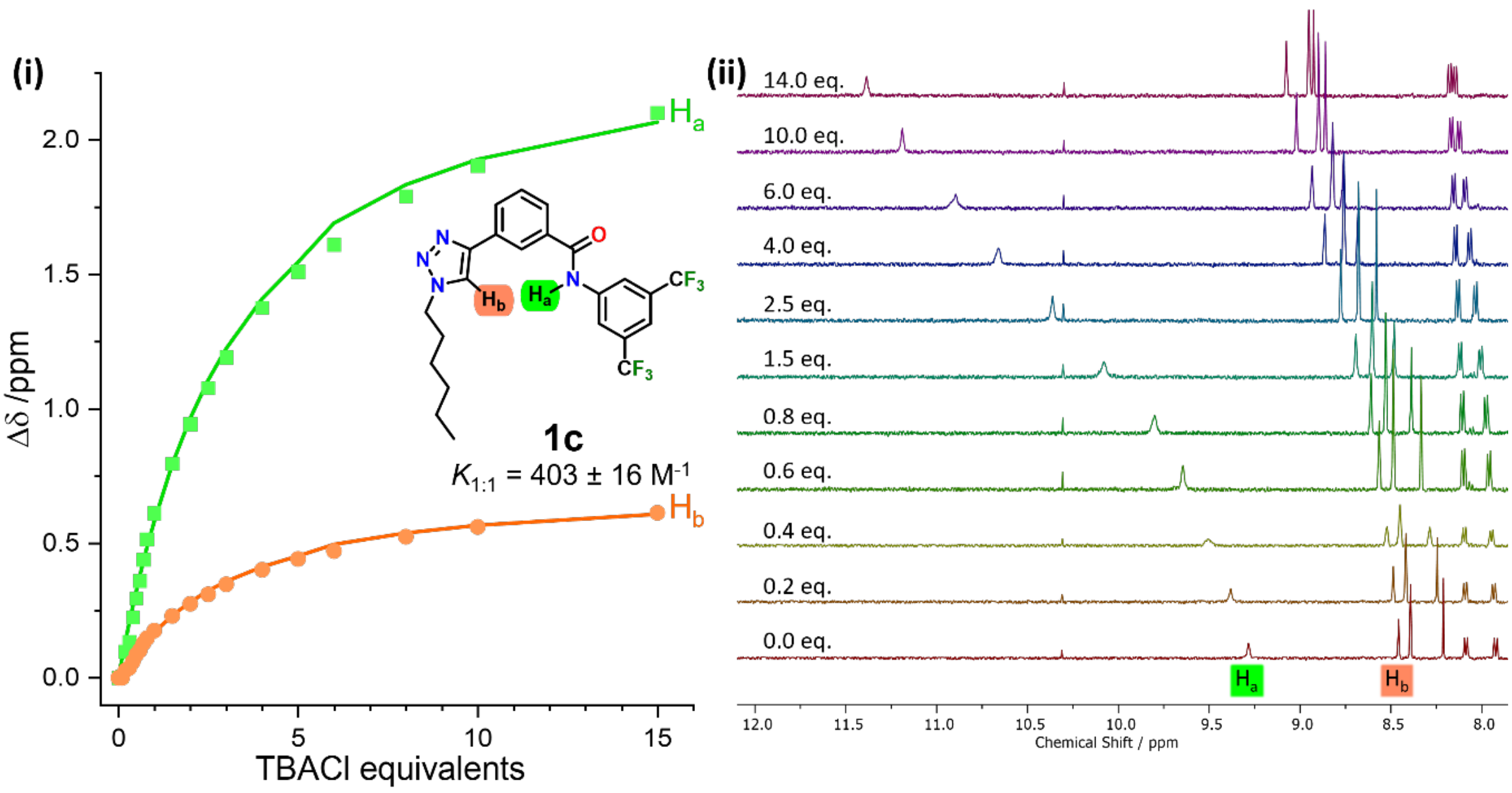


**Figure SI-22: (i)** Chloride binding isotherm for amido-triazole transporter **1c** in MeCN-$d_3$. Experimental data shown by symbols (■, ●), fitted 1:1 binding isotherm shown by solid lines (▬, ▬). **(ii)** Stacked partial $^1$H NMR Spectra of **1c** with increasing equivalents of TBACl.

**SI-Table 1:** Binding affinities of amido-triazole based anionophores in MeCN-$d_3$, fitted to a 1:1 stoichiometric host-guest binding model using BindFit v0.5.[8]

| 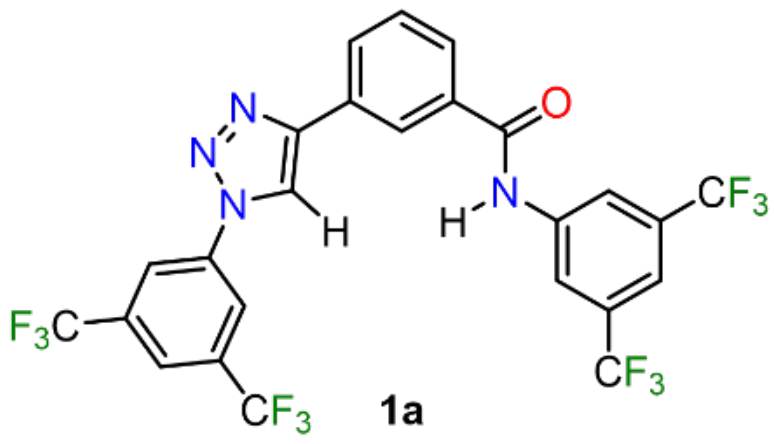 **1a** | 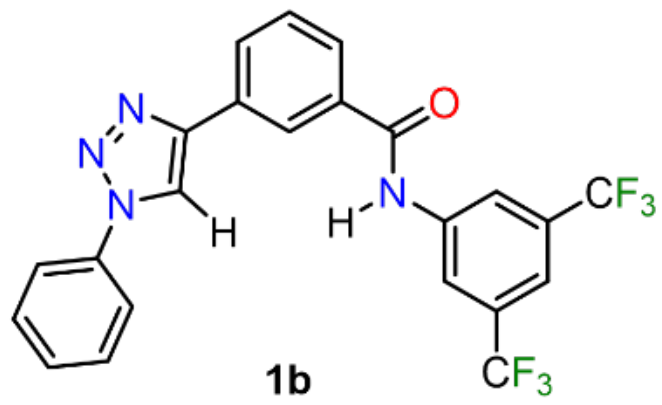 **1b** | 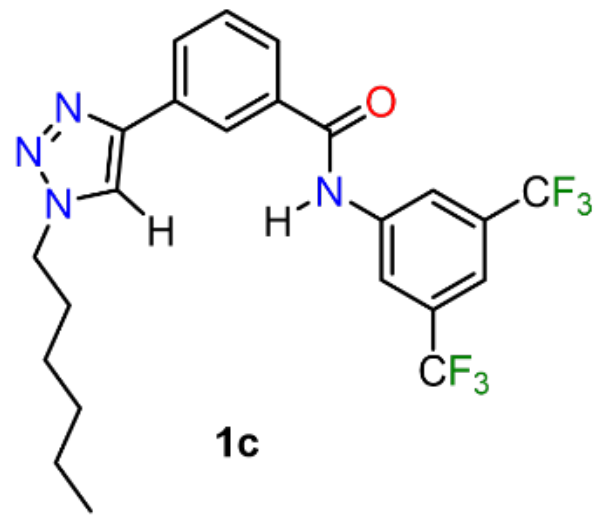 **1c** |
|---|---|---|
| $K_{1:1}$ = 1055 ± 56 $M^{-1}$ | $K_{1:1}$ = 742 ± 15 $M^{-1}$ | $K_{1:1}$ = 403 ± 16 $M^{-1}$ |

## 4 Photophysical Analysis

### 4.1 UV-Vis Absorbance

UV-Vis spectra were recorded on a V-770 UV-Visible/NIR Spectrophotometer equipped with Peltier temperature controller and stirrer using quartz cuvettes of 1 cm path length. Experiments were conducted at 25°C. Molar absorption coefficients, ε, were determined by recording UV-vis spectra for each compound, samples were dissolved in MeCN (UV-cut off 190 nm), at five concentrations and then preparing a Beer-Lambert plot of concentration against absorbance at $\lambda_{max}$, and plotted between 200 and 600 nm (**Figure SI-24**). UV-vis spectral overlaps of photocages and photocaged analogues, with the respective LED irradiation at 365 nm and 530 nm (**Figure SI-23**).

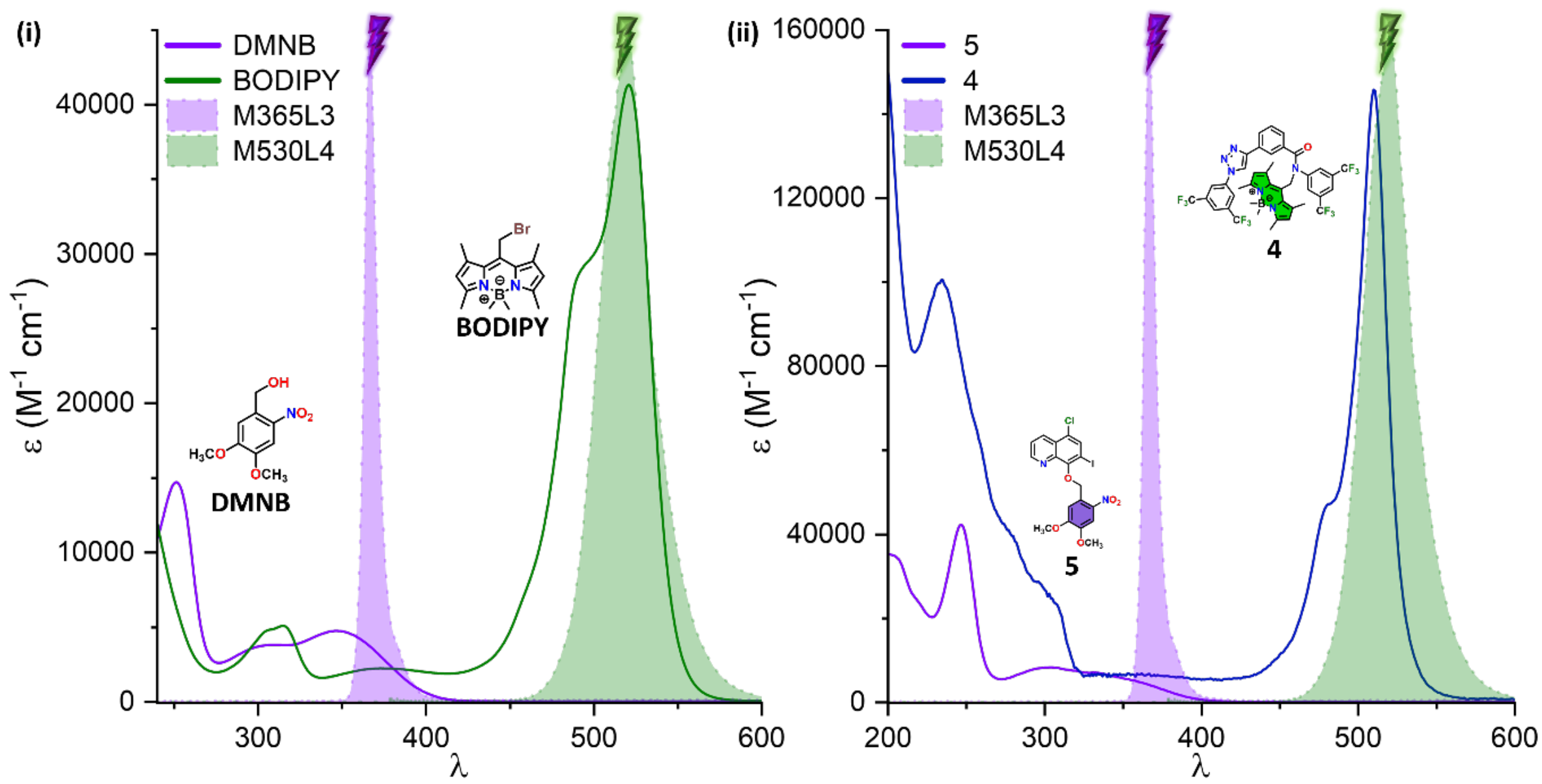


**Figure SI-23:** UV-visible absorbance spectra with the respective overlap of LED irradiation of **(i)** photocages dimethoxy-*o*-nitrobenzyl (DMNB) and BODIPY and **(ii)** photocaged analogues **4** and **5**.

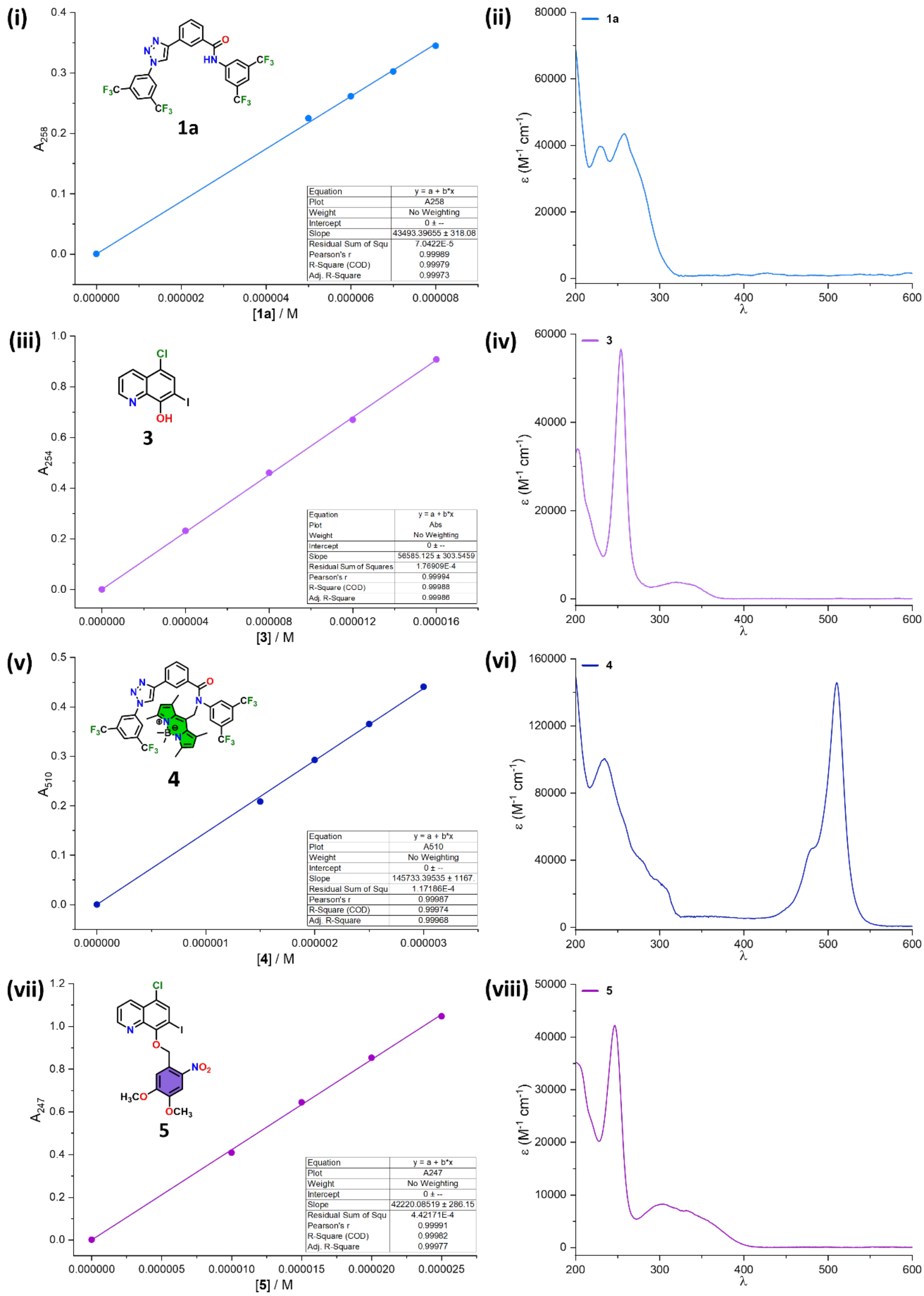


**Figure SI-24:** Beer-Lambert plot and UV-visible absorbance spectra of free transporters **1a** (**i-ii**), **3** (**iii-iv**), and their photocaged analogues **4** (**v-vi**) and **5** (**vii-viii**), respectively.

## 4.2 Fluorescence Excitation and Emission

Fluorescence spectroscopic data were recorded using a Horiba Duetta fluorescence spectrophotometer, equipped with Peltier temperature controller and stirrer using quartz cuvettes of 1 cm path length. Experiments were conducted at 25°C. A combined sample containing both **4** and **5** dissolved in MeCN, 1 µM and 20 µM respectively, was analysed at excitation wavelengths of 365 nm and 530 nm, and plotted as shown in **Figure SI-25.**

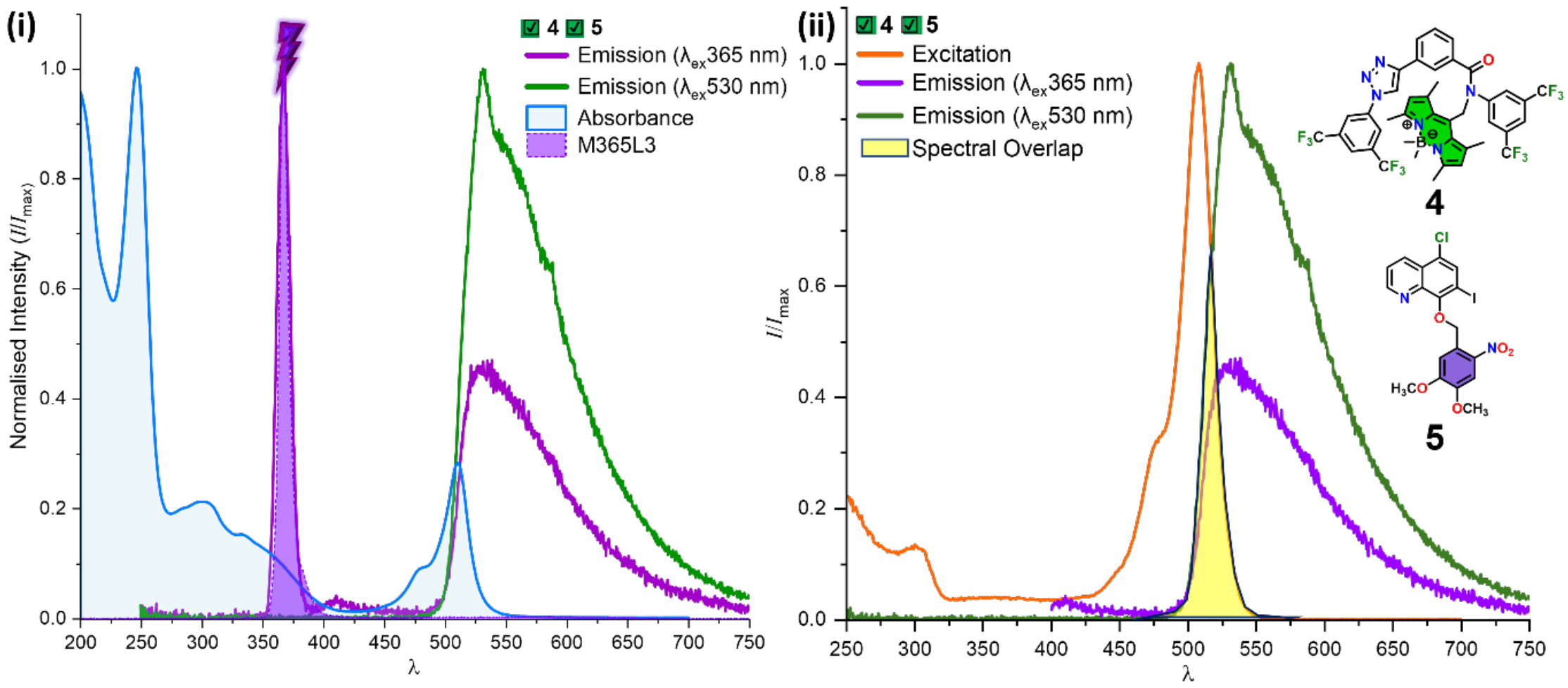


**Figure SI-25:** Combined sample of **4** (1 µM) and **5** (20 µM) in MeCN with fluorescence emission spectra from excitation as both 365 nm and 530 nm **(i)** UV-visible absorbance and emission spectra **(ii)** Excitation and emission spectra showing highlighted spectral overlap.

# 5 Photocleavage Experiments

## 5.1 Irradiation Set-up

Photo-irradiation of samples was carried out using Thorlabs high-power mounted LEDs (M530L4, 530 nm, 370 mW; M365L3, 365 nm, 880 mW) using an in-house custom-built set-up with optical components supplied by Thorlabs (**Figure SI-26**).[10] For irradiating small vials of samples, NMR tubes and cuvettes, a Thorlabs cuvette holder (CVH100/M) equipped with the mounted LEDs was used. Samples were irradiated for various times to reach the photocleaved state, as confirmed by $^{1}$H NMR or UV-vis experiments. The same set-up was used for *in situ* transport experiments in LUVs, whereby the cuvette containing fluorophore encapsulated vesicles and photo-caged transporter(s) was placed in the cuvette holder and covered for specific irradiation times.

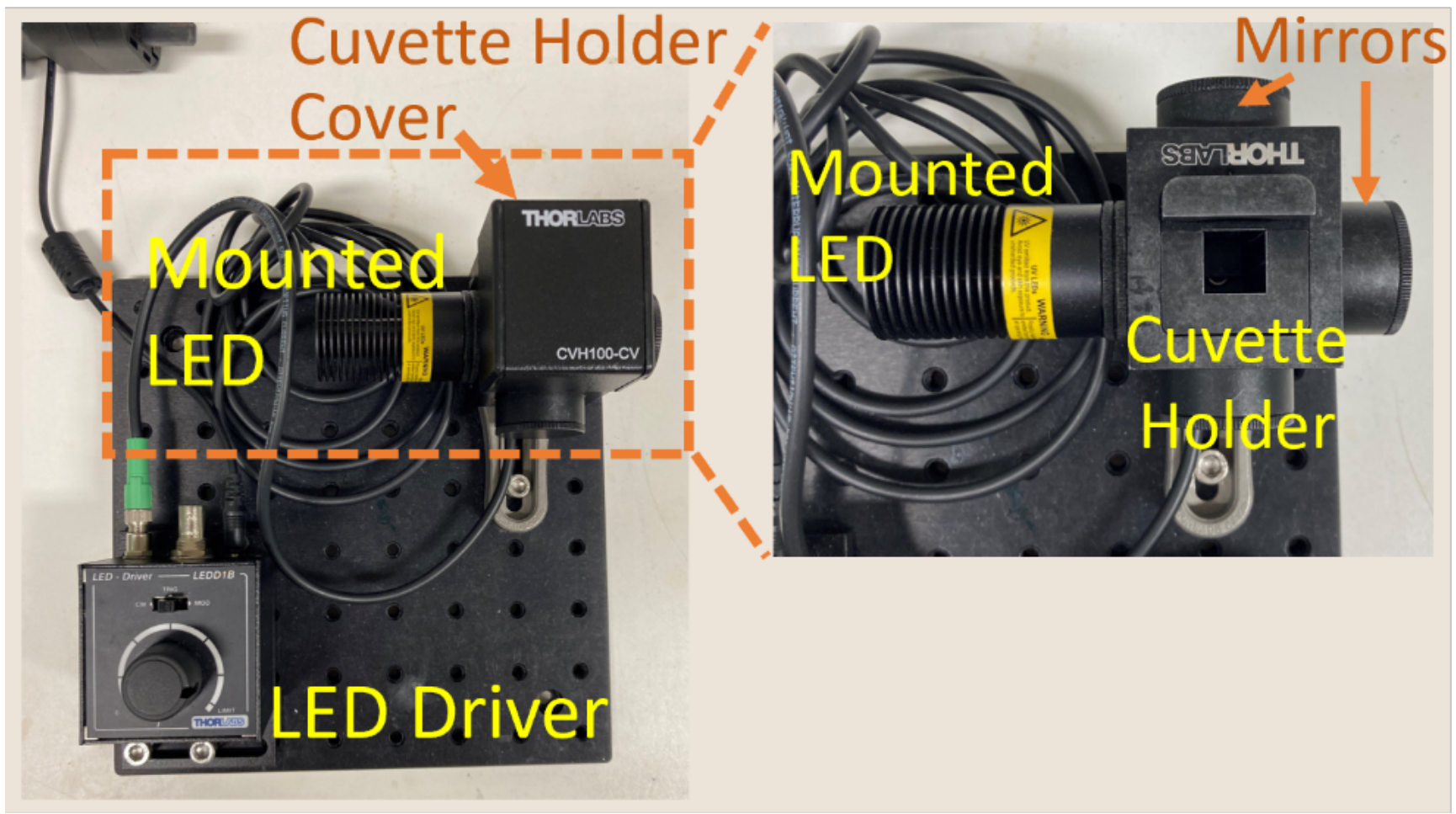


**Figure SI-26:** In-house custom-built set-up for irradiating samples for de-caging analysis by $^{1}$H NMR, UV-Vis, and for LUV transport experiments.

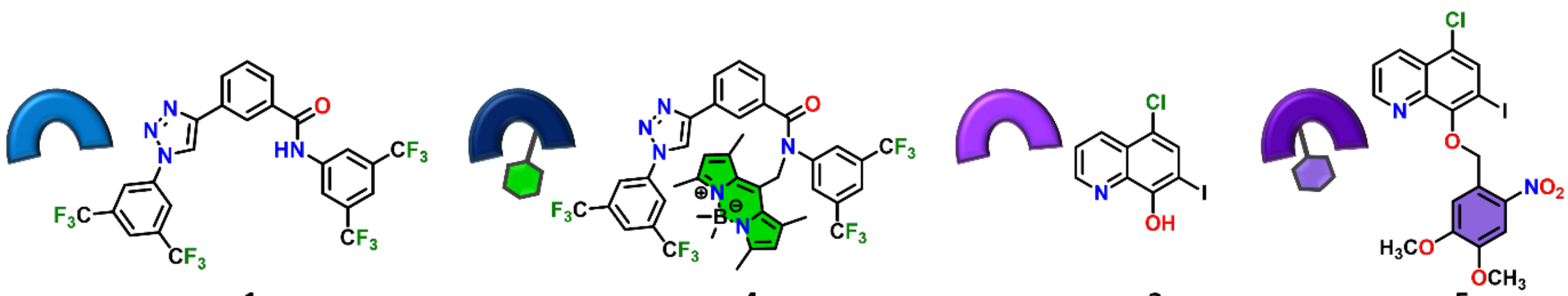

1a 4 3 5

**Figure SI-27:** Photocaged ionophores, and their respective free forms for reference, used in photocleavage and photoactivated experiments.

## 5.2 $^1$H NMR Analysis

Photocleavage of **4** and **5** was conducted in the solution phase by irradiating 1 mM samples in an NMR tube with Thorlabs mounted LEDs at 530 nm (M530L4, 370 mW) or 365 nm (M365L3, 880 mW) respectively, in a Thorlabs cuvette holder (CVH100/MM). The LED was supplied with 1 A current using Thorlabs T-cub LED driver (LEDD1B). The deprotection was monitored using $^1$H NMR experiments at known time intervals.

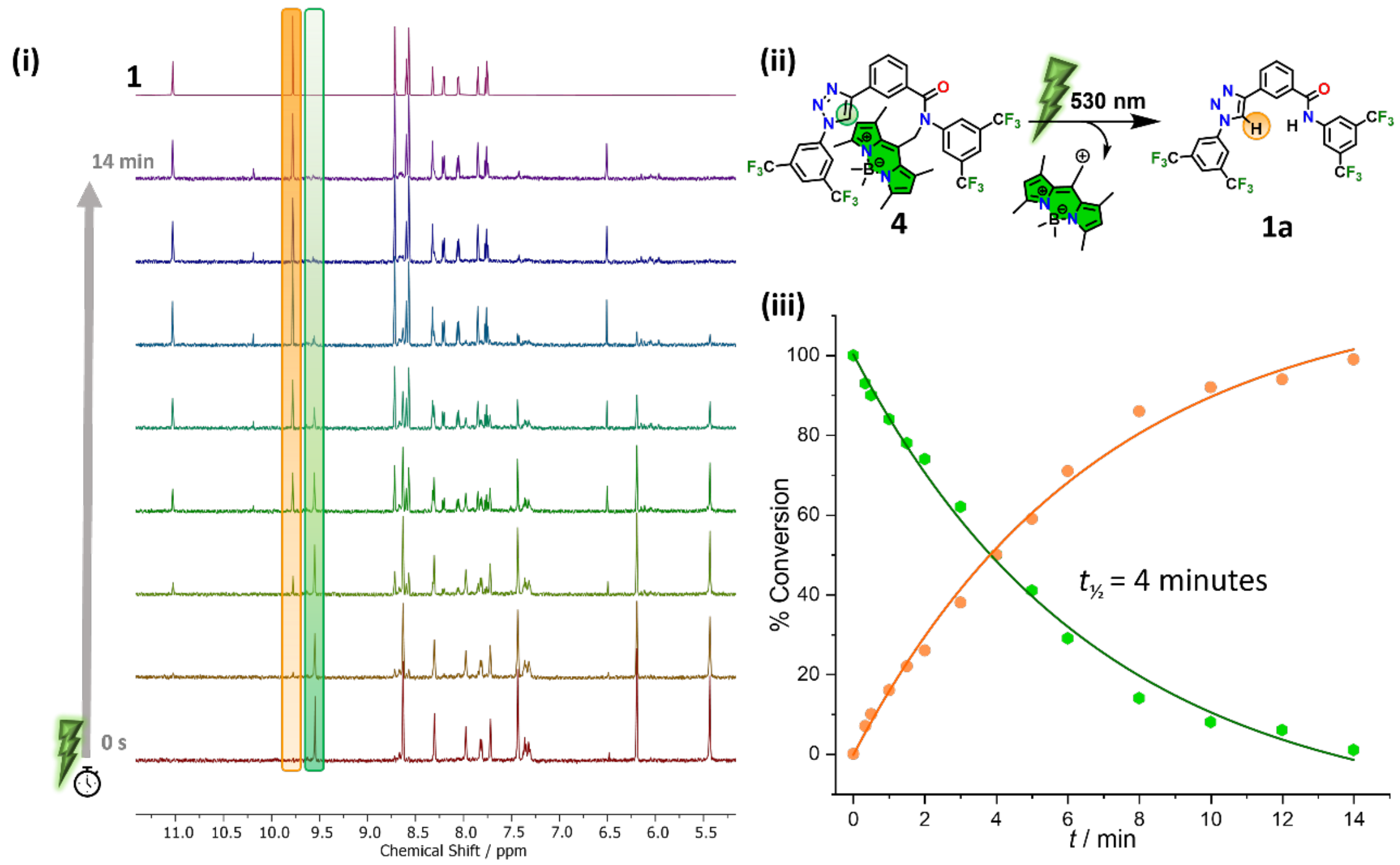


**Figure SI-28: (i)** Stacked partial $^1$H NMR Spectra of **4** (1 mM sample in DMSO-$d_6$, 298 K) with increasing irradiation times (0 seconds – 14 minutes) with a 530 nm LED (370 mW); the spectrum of the free transporter **1a** is included for reference. **(ii)** Schematic representation of the photo-activation of free anionophore **1a**. **(iii)** Conversion of **4** after irradiation with a 530 nm LED (370 mW).

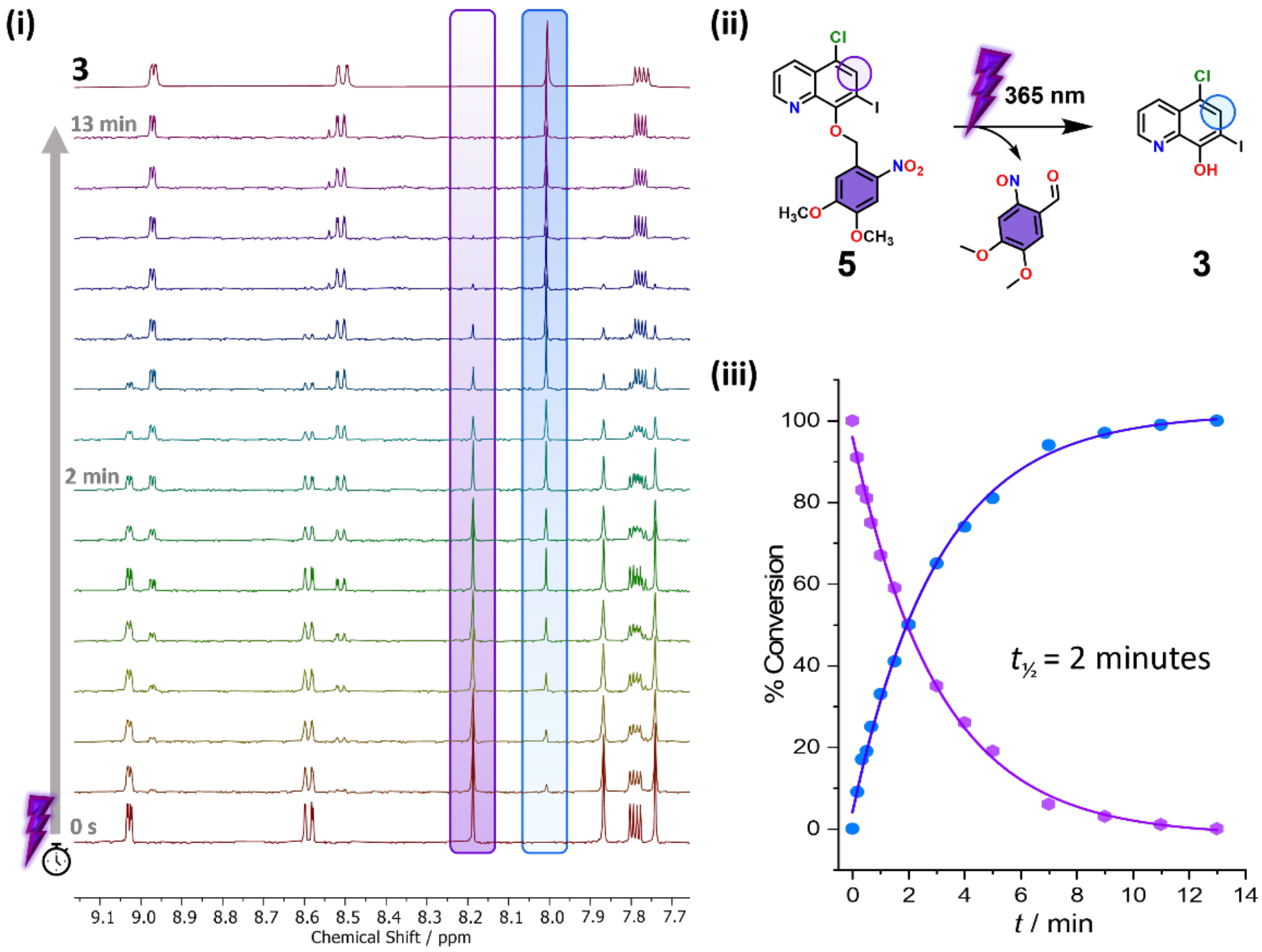


**Figure SI-29: (i)** Stacked partial $^{1}H$ NMR Spectra of **5** (1 mM sample in DMSO-$d_6$, 298 K) with increasing irradiation times (0 seconds – 13 minutes) with a 365 nm LED (880 mW); the spectrum of the free transporter **3** is included for reference. **(ii)** Schematic representation of the photo-activation of free cationophore **3**. **(iii)** Conversion of **5** after irradiation with a 365 nm LED (880 mW).

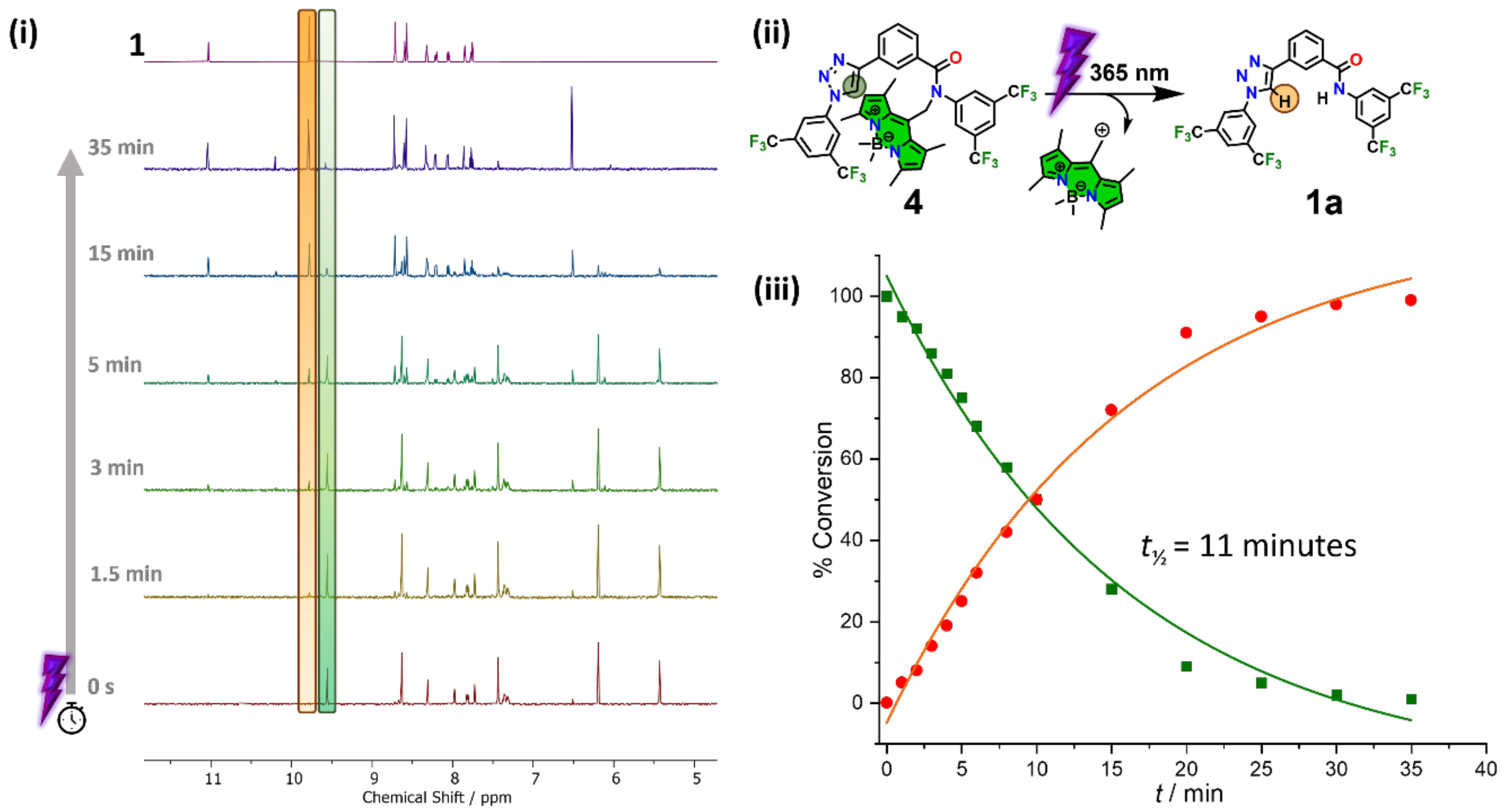


**Figure SI-30: (i)** Stacked partial $^{1}$H NMR Spectra of **4** (1 mM sample in DMSO-$d_6$, 298 K) with increasing irradiation times (0 seconds – 35 minutes) with a 365 nm LED (880 mW); the spectrum of the free transporter **1a** is included for reference. **(ii)** Schematic representation of the photo-activation of free anionophore **1a**. **(iii)** Conversion of **4** after irradiation with a 365 nm LED (880 mW).

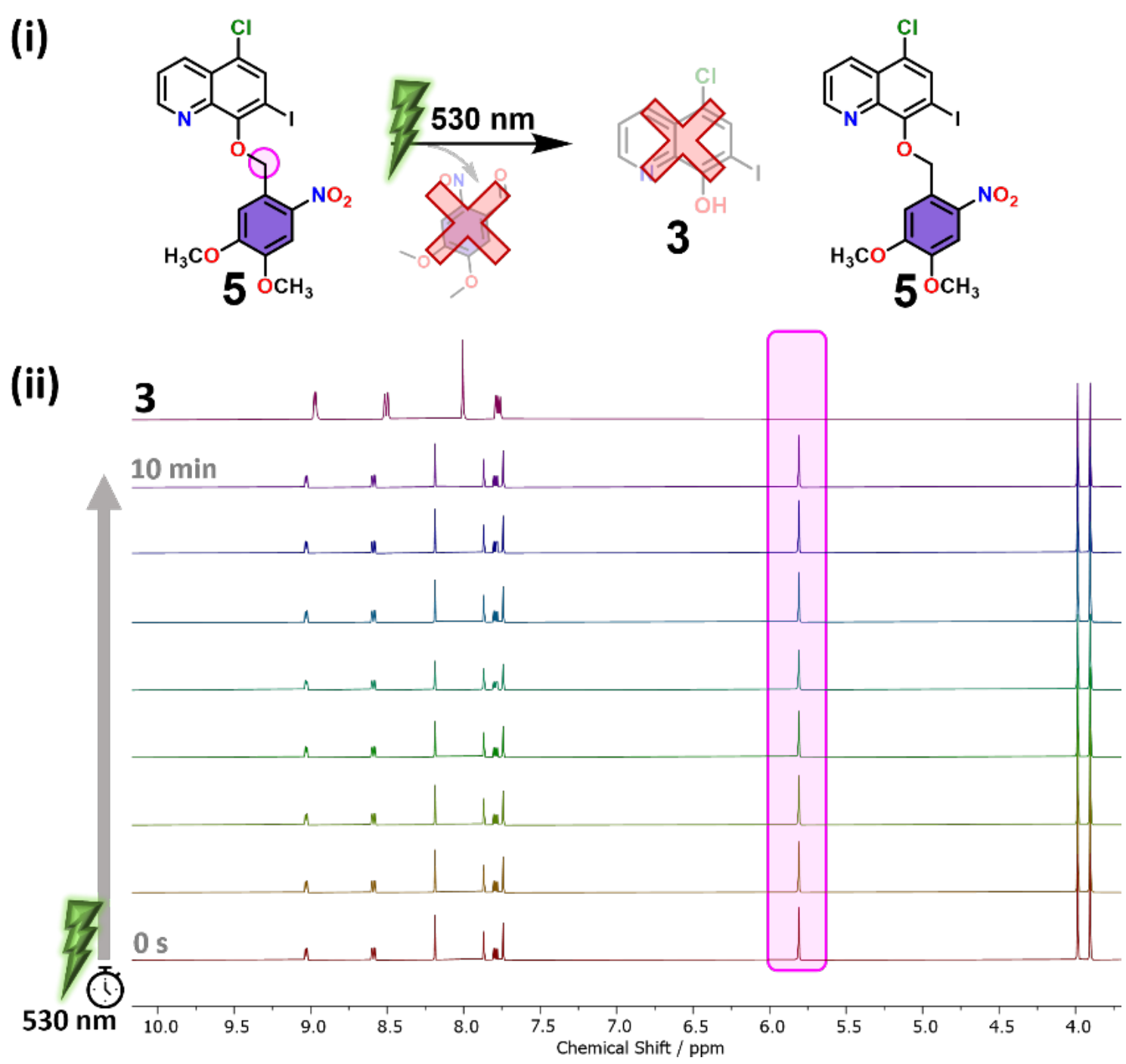


**Figure SI-31:** Stacked partial $^{1}$H NMR Spectra of **5** (1 mM sample in DMSO-$d_6$, 298 K) with increasing irradiation times (0 seconds – 10 minutes) with a 530 nm LED (370 mW).

## 6 Anion Transport Experiments

### 6.1 Vesicle Preparation for HPTS Assays[11]

A thin film of lipid (1-palmitoyl-2-oleoyl-*sn*-3-phosphatidylcholine (POPC) or dipalmitoyl phosphatidylcholine (DPPC)) was formed by evaporating a chloroform solution of the lipid under reduced pressure on a rotary evaporator (40 °C) and then under high vacuum for 6 hours. Then the lipid film was hydrated by vortexing with 1 mL of the prepared internal buffer solution. The lipid suspension was then subjected to 5 freeze-thaw cycles using liquid nitrogen and a water bath (40°C) followed by extrusion 19 times through a polycarbonate membrane (pore size 200 nm). Extrusion was performed at 50°C in the case of DPPC lipids. Extra-vesicular components were removed by size exclusion chromatography on a Sephadex G-25 column with 100 mM NaCl, 10 mM HEPES, pH 7.0. Final conditions: LUVs (2.5 mM lipid); inside 100 mM NaCl, 10 mM HEPES, 1 mM 8-Hydroxypyrene-1,3,6-trisulfonic acid trisodium salt (HPTS), pH 7.0; outside: 100 mM NaCl, 10 mM HEPES, pH 7.0. Vesicles for the sodium gluconate assay, and the MCl cation selectivity assay, were prepared by the same procedure, substituting NaCl for NaGluconate, or using other MCl salts in the buffer solution.

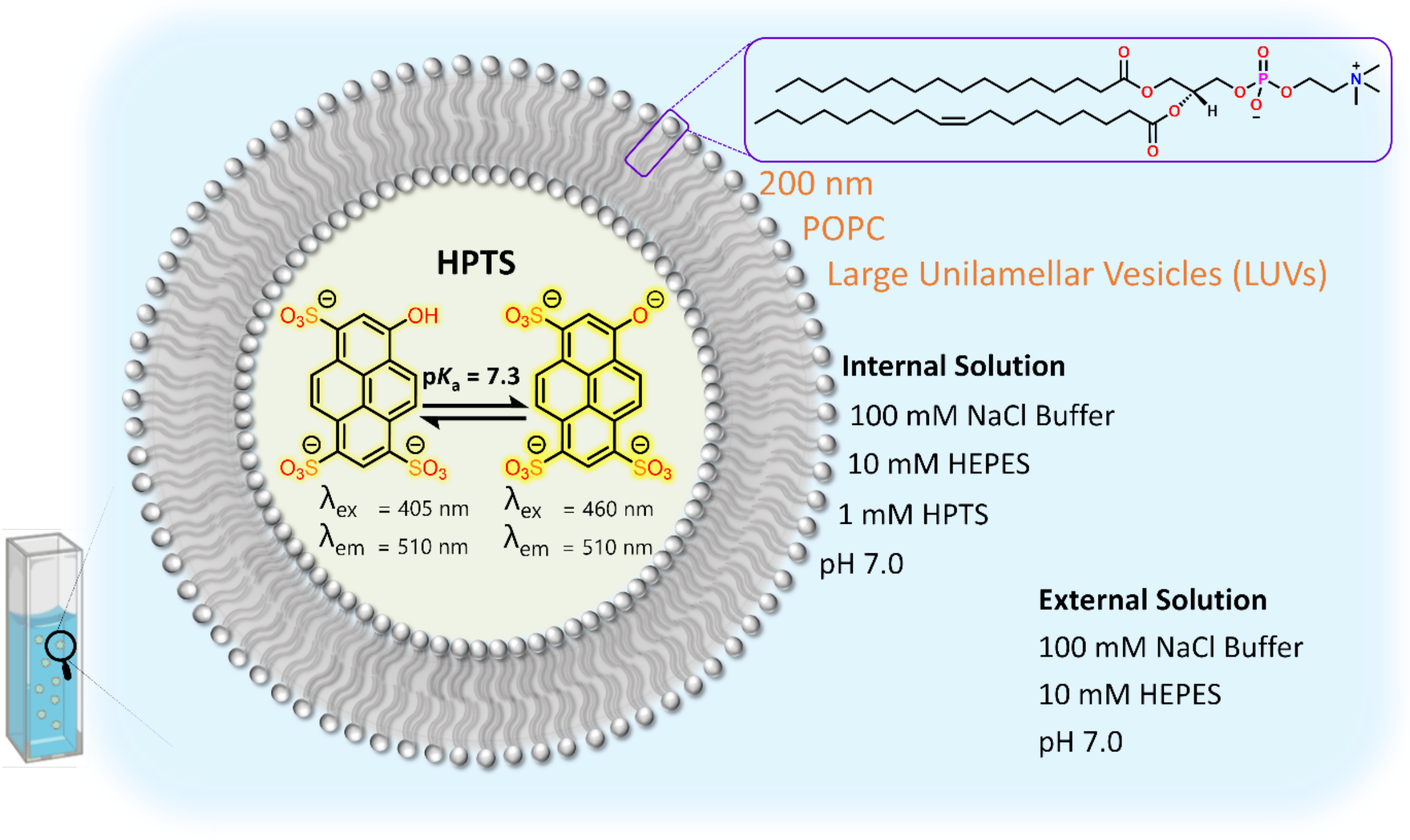


**Figure SI-32:** POPC vesicles encapsulating HPTS probe (POPC-LUVs⊃HPTS) to monitor ion transport across the lipid membrane.

### 6.1.1 The Standard HPTS Assay

In a typical experiment, the LUVs containing HPTS (25 µL, final lipid concentration 31 µM) were added to buffer (1950 µL of 100 mM NaCl, 10 mM HEPES, pH 7.0) at 25°C under gentle stirring. A pulse of NaOH (20 µL, 0.5 M – final concentration 5 mM) was added at 40 seconds to initiate the experiment. At 90 seconds the test transporter (various concentrations, 5 µL in DMSO) was added, followed by detergent (25 µL of Triton X-100 in 7:1 (v/v) $H_2O$-DMSO) at 290 seconds to lyse the vesicles for calibration. The fluorescence emission was monitored at $\lambda_{em}$ = 510 nm ($\lambda_{ex}$ = 460/405 nm). For transporter **1**, a Hill plot was fitted to 12 data points spanning the required concentration range, and each individual concentration was repeated at-least twice and averaged. The fractional fluorescence intensity ($I_{rel}$) was calculated using Equation S1, where $R_t$ is the fluorescence ratio at time t, $R_0$ is the fluorescence ratio at time 0, and $R_d$ is the fluorescence ratio after the addition of detergent.

$$I_{\mathrm{rel}} = \frac{R_t - R_0}{R_d - R_0} \qquad \text{(S1)}$$

The fractional fluorescence intensity ($I_{rel}$) at 276 s just prior to lysis, defined as the fractional activity $y$, was plotted as a function of the transporter concentration (x / mol%). Hill coefficients (n) and $EC_{50}$ values were calculated by fitting to the Hill equation (S2):

$$y = y_0 + (y_{max} - y_0 \frac{x^n}{EC_{50}^n + x^n} \qquad \text{(S2)}$$

where $y_0$ is the fractional activity in the absence of transporter, $y_{max}$ is the fractional activity with excess transporter, x is the transporter concentration in the cuvette.

The $k_{ini}$ values were calculated by fitting the transport kinetics curves ($I_{rel}$ vs $t$) with the exponential function $I_{rel}$ = a – exp(-$t$/b) using Origin 2025. $k_{ini}$ (the initial rate of transport immediately after the base pulse) is then given by differentiation as $k_{ini}$ = 1/b ($s^{-1}$).

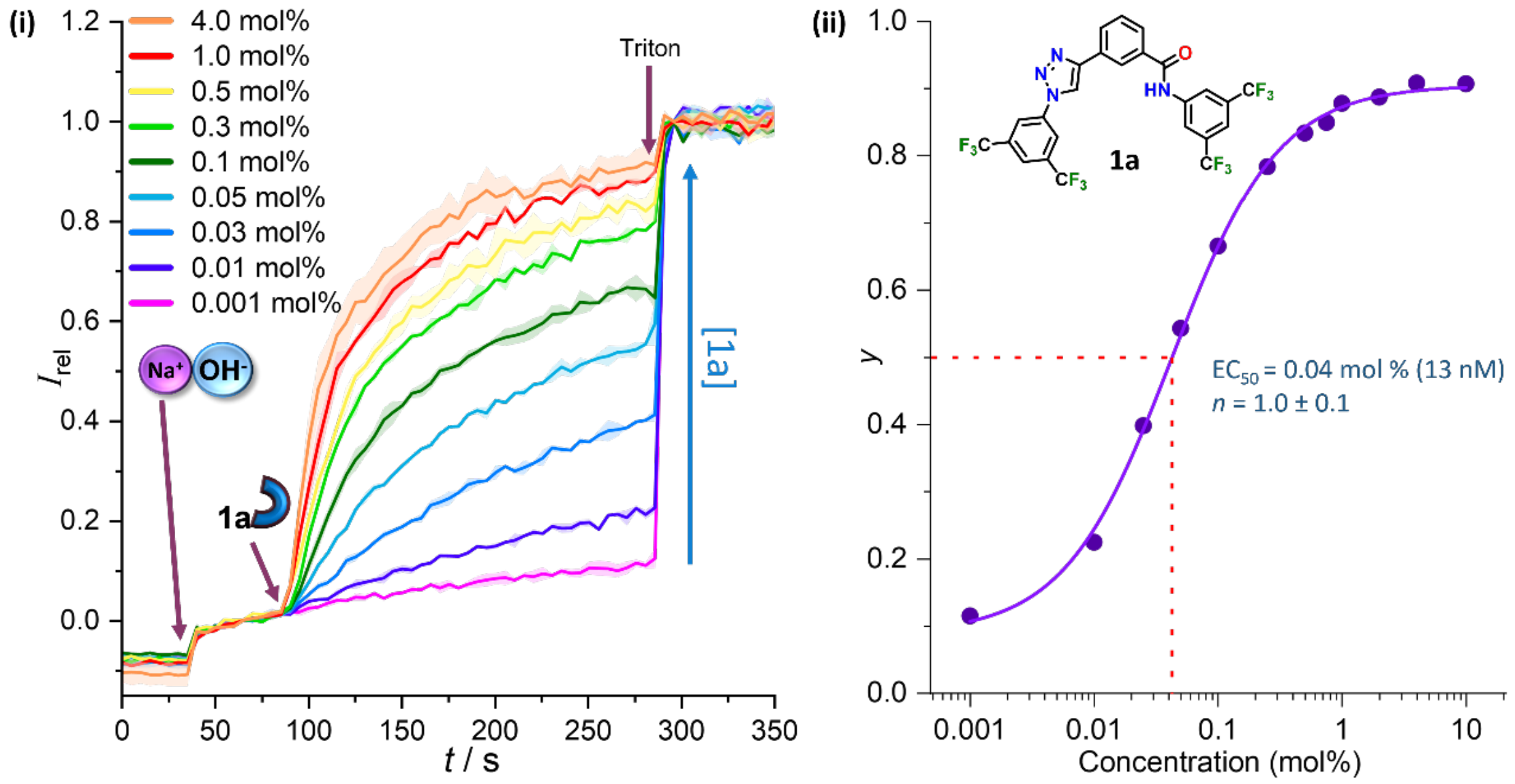


**Figure SI-33: (i)** Change in ratiometric emission, $I_{rel}$ ($\lambda_{em}$ = 510 nm; $\lambda_{ex1}$ = 405 nm, $\lambda_{ex2}$ = 460 nm) with increasing concentration of anionophore **1a**, upon addition of a NaOH base pulse (5 mM) to POPC LUVs (31 µM) containing 1 mM HPTS, 100 mM internal and external NaCl, buffered with 10 mM HEPES at pH 7.0. **(ii)** Dependence on fractional activities (*y*, the relative intensity at *t* = 276 s immediately prior to lysis) on concentration of **1a**, (●), and fitted to the Hill equation (▬)

### 6.1.2 HPTS Assay with DPPC Lipids

The HPTS assay was carried out using the same procedure as described above, however, the POPC LUVs were replaced with dipalmitoyl phosphatidylcholine (DPPC) LUVs. These lipids are extruded at 50 °C. Experiments with DPPC lipids were conducted in the same way as described above. For elevated temperature studies, the buffer was equilibrated at 45 °C for 5 minutes prior to initiating the experiment.

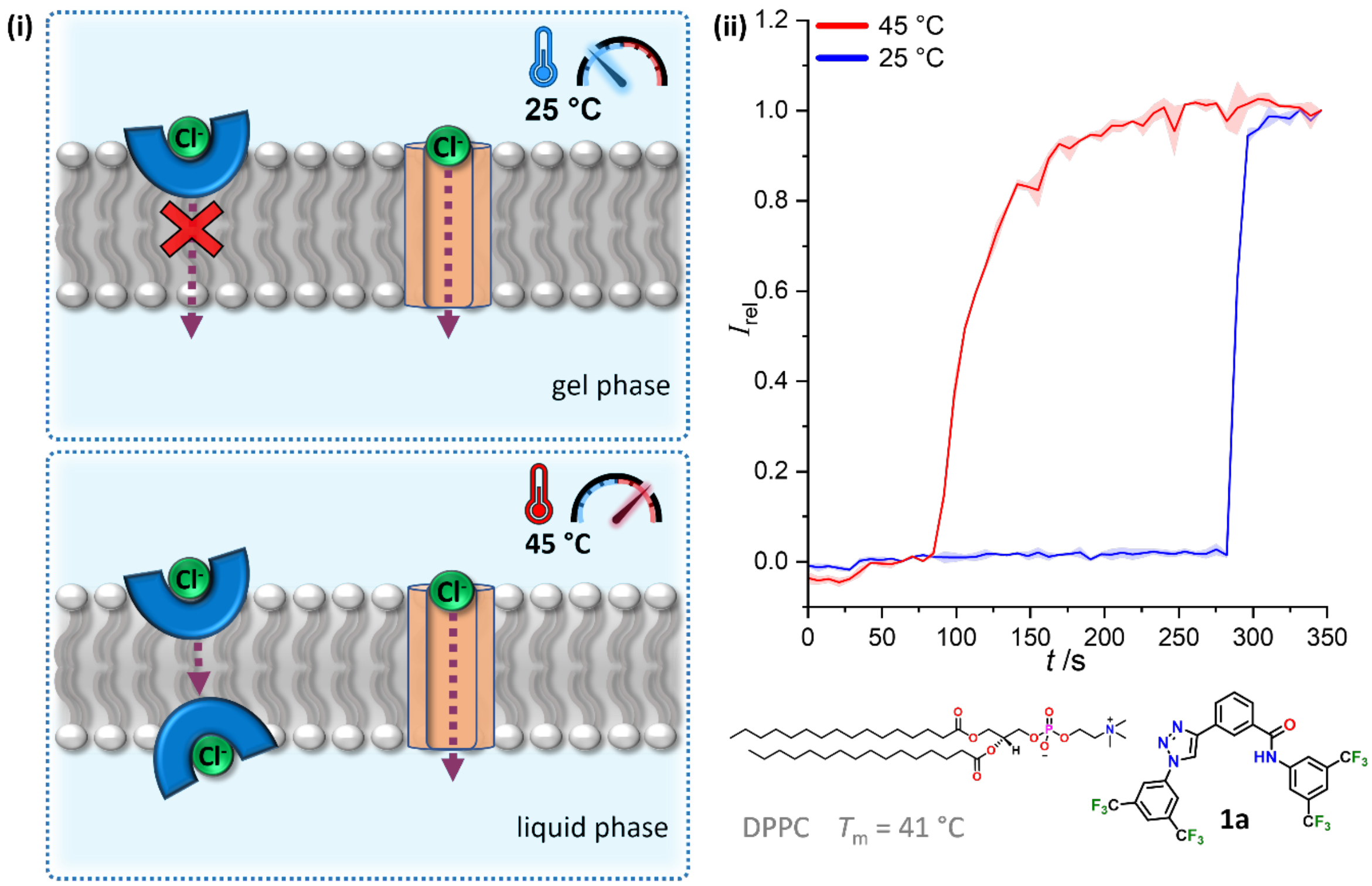


**Figure SI-34: (i)** Schematic diagram of the DPPC-LUVs⊃HPTS assay, where ion carriers are inhibited in the gel phase but mobile in the liquid phase of DPPC lipids. Ion channels are capable of allowing ion passage in either phase. **(ii)** Ion transport HPTS assay data for anionophore **1a** (0.1 mol% w.r.t lipid) in DPPC LUVs at 25 °C and 45 °C.

### 6.1.3 HPTS Assay with NaX Buffers

POPC LUVs were prepared with an internal solution of 100 mM NaCl, 10 mM HEPES, 1 mM HPTS buffered to pH 7.0 and an external buffer of 100 mM NaX (where X = Cl, $NO_3$, Br, I), 10 mM HEPES, pH 7.0. In the case of NaI buffer, 5 mM $Na_2S_2O_3$ was added. This assay was performed with **1a** at concentration of 31.3 nM (0.1 mol% transporter with respect to lipid). A cuvette was prepared with POPC LUVs and external NaX buffer. **1a** was incubated for 5 minutes, to allow for anion exchange, before starting the fluorescent recording. At 40 s, a NaOH base pulse (5 mM) was added and at 290 s Triton X-100 was added to lyse the vesicles. Each run was repeated at least twice.

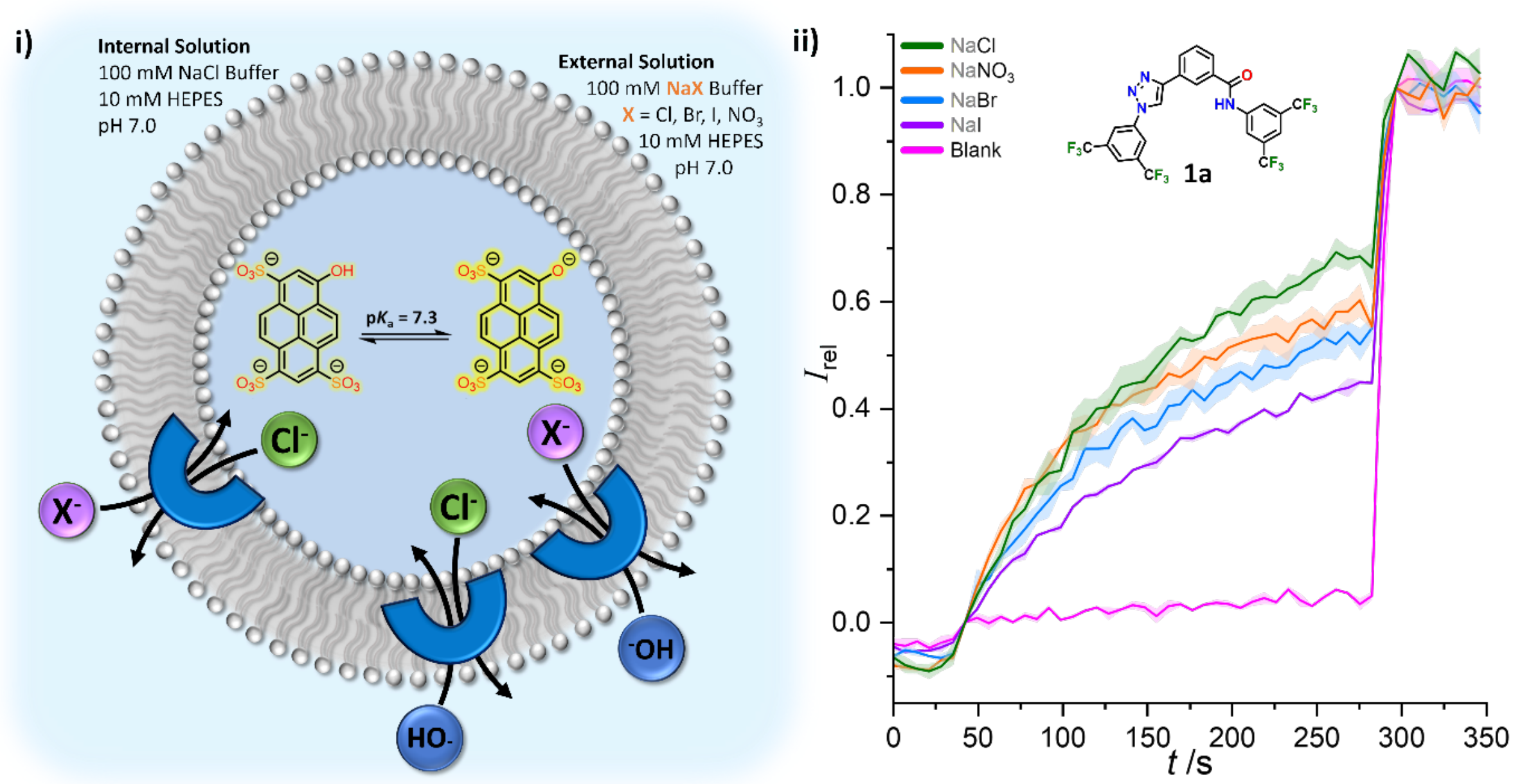


**Figure SI-35: (i)** Schematic diagram of the POPC-LUVs⊃HPTS assay with internal NaCl buffer and external NaX buffers, where X = Cl, $NO_3$, Br, I. **(ii)** Ion transport data for anionophore **1a** (0.1 mol% w.r.t lipid) in POPC LUVs with a variety of external NaX buffers.

### 6.1.4 HPTS Assay with Sodium Gluconate

POPC LUVs were prepared with an internal solution of 100 mM sodium gluconate, 10 mM HEPES, 1 mM HPTS buffered to pH 7.0 and an external buffer of 100 mM sodium gluconate, 10 mM HEPES, pH 7.0. This assay was performed with **1a** at concentration of 31.3 nM (0.1 mol% transporter with respect to lipid) and repeated at least twice.

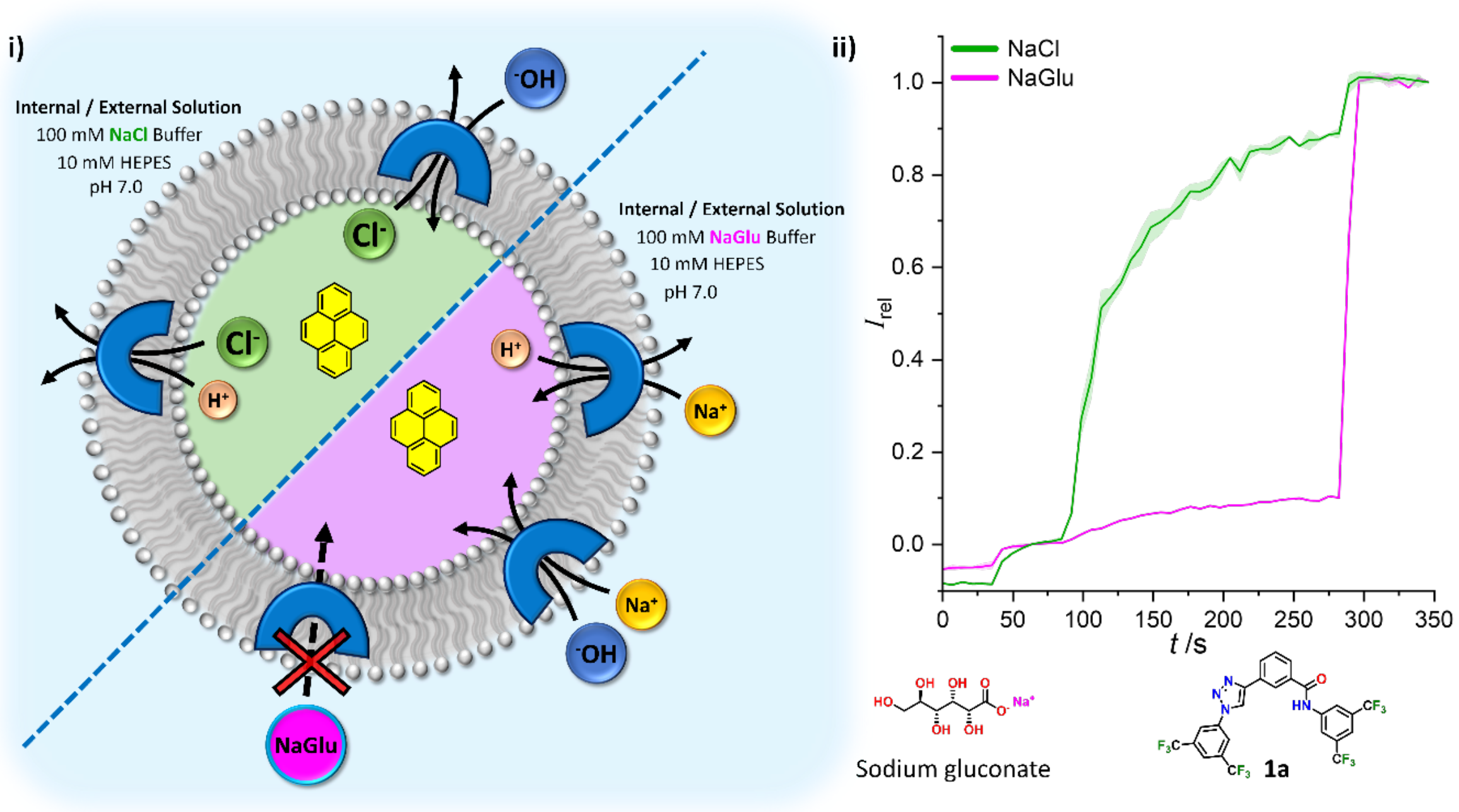


**Figure SI-36: (i)** Schematic diagram of the POPC-LUVs⊃HPTS assay with either NaCl or NaGlu buffers. **(ii)** ion transport data for anionophore **1a** (0.1 mol% w.r.t lipid) in POPC LUVs with sodium gluconate (NaGlu) buffer (▬), with comparison of ion transport activity at the same mol% concentration with sodium chloride (NaCl) buffer (▬).

### 6.1.5 HPTS Assay with MCl Buffers

POPC LUVs were prepared with an internal solution of 100 mM NaCl, 10 mM HEPES, 1 mM HPTS buffered to pH 7.0 and an external buffer of 100 mM MCl (where M = Na, K, Li, Rb, Cs), 10 mM HEPES, pH 7.0. This assay was performed with **1a** at concentration of 31.3 nM (0.1 mol% transporter with respect to lipid) and repeated at least twice.

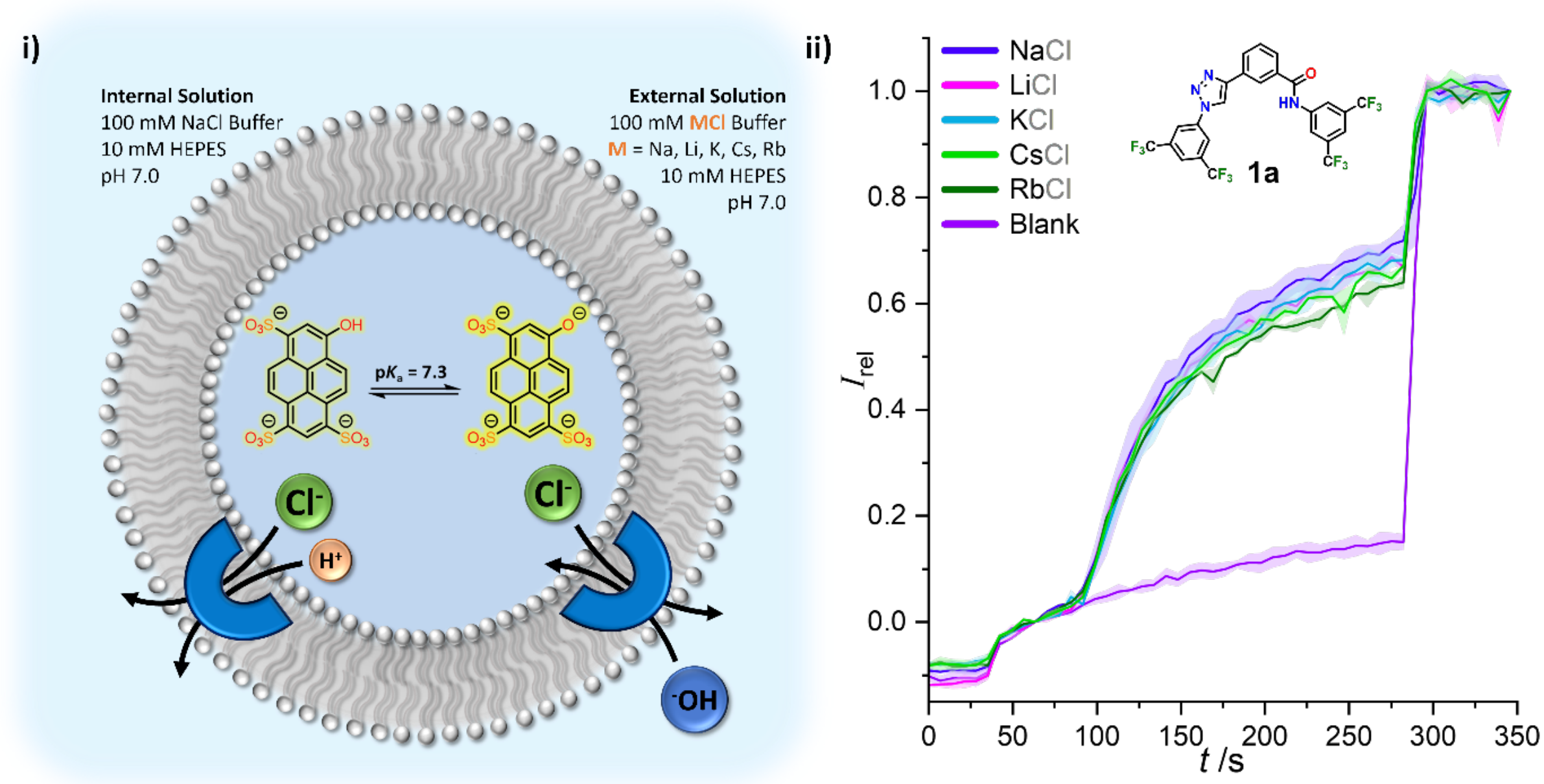


**Figure SI-37: (i)** Schematic diagram of the POPC-LUVs⊃HPTS assay with internal NaCl buffer and external MCl buffers, where M = Na, Li, K, Cs, Rb. **(ii)** Ion transport data for anionophore **1a** (0.1 mol% w.r.t lipid) in POPC LUVs with a variety of external MCl buffers.

### 6.1.6 HPTS Assay with FCCP

Experiments in the presence of protonophore trifluoromethoxy carbonylcyanide phenylhydrazone (FCCP) were carried out using the above procedure, except that a DMSO solution of FCCP (5 µL of 100 µM solution, final concentration 0.25 µM/0.8 mol%) was added to the vesicle suspension prior to the addition of the NaOH pulse, but after the irradiation step. At this concentration, FCCP does not cause appreciable dissipation of the transmembrane pH gradient alone.

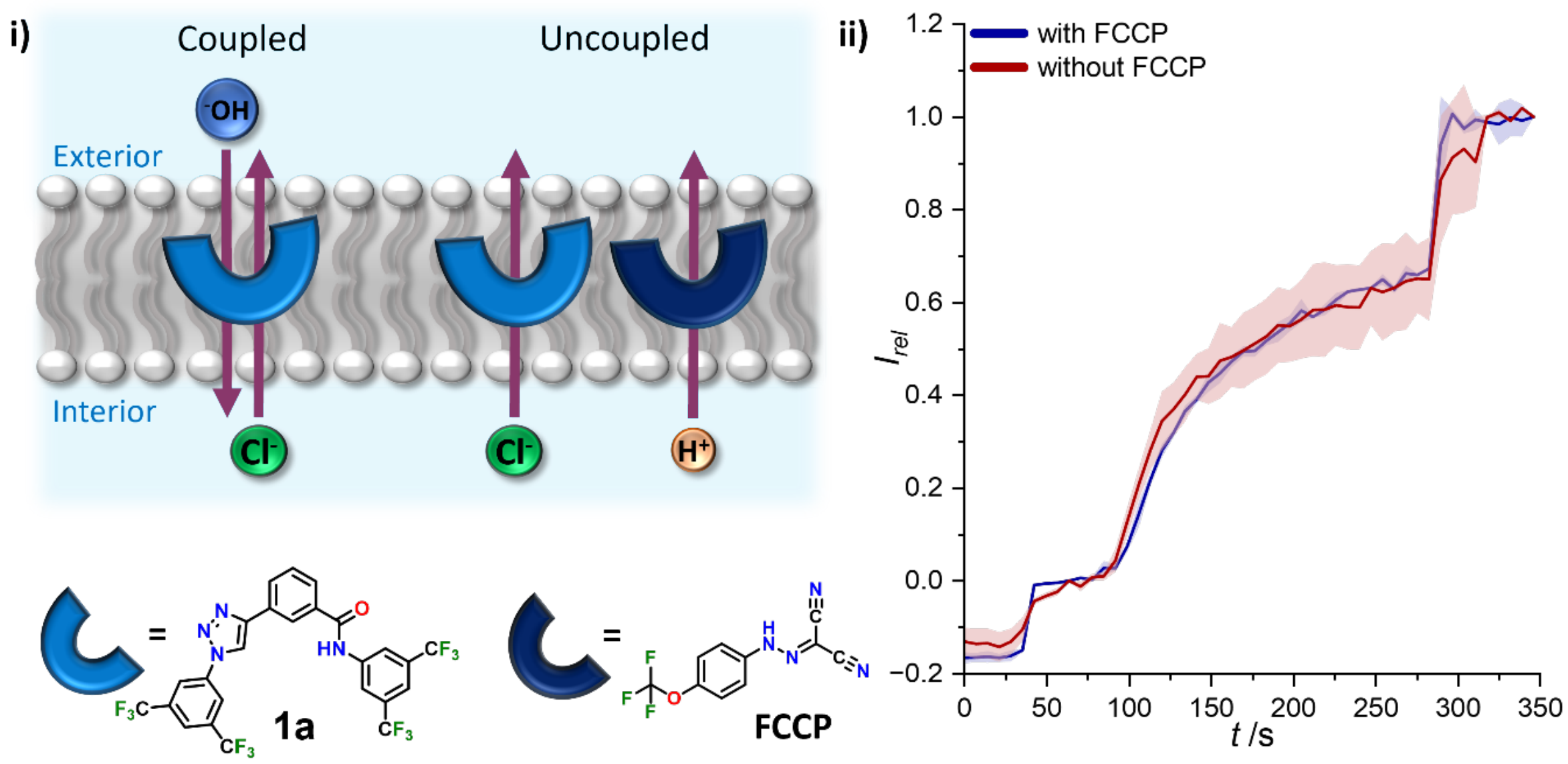


**Figure SI-38: (i)** Schematic diagram of the POPC-LUVs⊃HPTS assay conducted in the presence of FCCP. **(ii)** Ion transport HPTS assay data for anionophore **1a** (0.1 mol% w.r.t lipid) in POPC LUVs with 100 mM NaCl in 10 mM HEPES buffer in the presence and absence of FCCP (5 µL of 100 µM solution in DMSO) added prior to start of the experiment.

## 6.2 Vesicle Preparation for Magnesium Green (MgG) Assays[7]

A thin film of lipid (1-palmitoyl-2-oleoyl-*sn*-3-phosphatidylcholine (POPC) was formed by evaporating a chloroform solution under reduced pressure on a rotary evaporator and then under high vacuum (6 hours). The lipid film was hydrated by vortexing with the prepared buffer (100 mM NaCl, 10 mM 4-(2-hydroxyethyl)-1-piperazineethanesulfonic acid (HEPES), 50 µM magnesium green pentapotassium salt (MgG) and 10 µM ethylenediaminetetraacetic acid (EDTA) at pH 7.0. The lipid suspension was then subjected to 5 freeze-thaw cycles using liquid nitrogen and a water bath (40 °C), followed by extrusion 19 times through a polycarbonate membrane (pore size 200 nm). Extravesicular components were removed by size exclusion chromatography on a Sephadex G-25 column with prepared buffer (100 mM NaCl, 10 mM HEPES pH 7.0). Final conditions: large unilamellar vesicles (LUVs) (2.5 mM lipid); inside 100 mM NaCl, 10 mM HEPES, 10 µM EDTA, 50 µM MgG; outside 100 mM NaCl, 10 mM HEPES, pH 7.0.

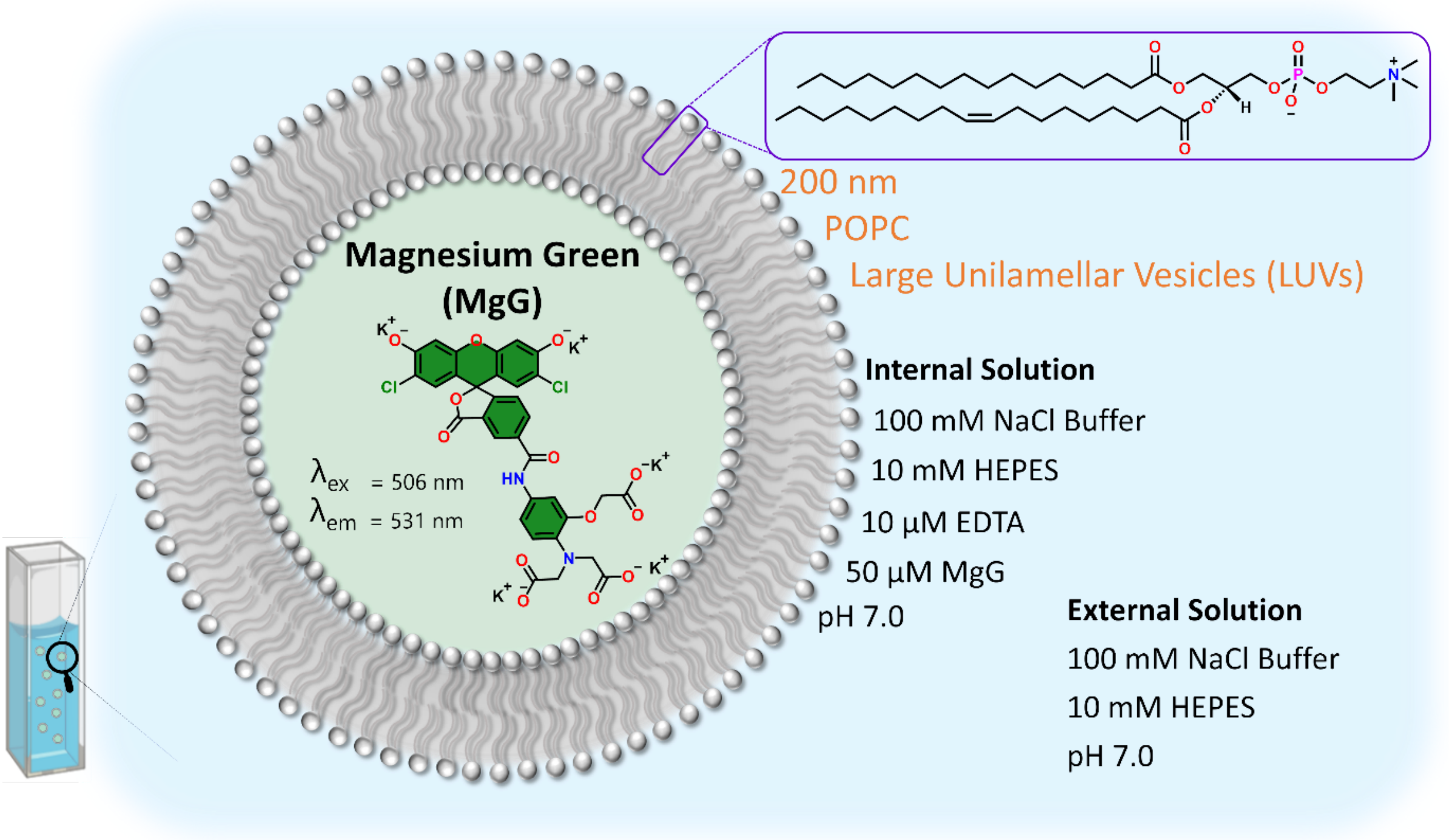


**Figure SI-39:** POPC vesicles encapsulating Magnesium Green (MgG) probe (POPC-LUVs⊃MgG) to monitor zinc transport across the lipid membrane.

### 6.2.1 The Magnesium Green (MgG) Assay

In a typical experiment, the LUVs containing MgG (80 µL, final concentration 100 µM lipid) were added to buffer (1895 µL of 100 mM NaCl, 10 mM HEPES, pH 7.0) at 25 °C under gentle stirring. A pulse of $ZnCl_2$ (20 µL, 10 mM) was added to initiate the experiment, before the test transporter (5 µL in DMSO) was added. The fluorescence emission was monitored over time at $\lambda_{em}$ = 531 nm ($\lambda_{ex}$ = 506 nm). The data was normalised using Equations S3:

$$I = \frac{I_{raw}}{I_{63}} - 1 \qquad \text{(S3)}$$

where $I_{raw}$ is the raw fluorescence intensity value, $I_{63}$ is the fluorescence intensity at 63 s (time point after the $Zn^{2+}$ gradient is established over the lipid membrane but prior to transporter addition). Normalisation to 100 % transport (achieved by addition of 0.1 mol% of **3**) was carried out using Equation S4:

$$I_{\mathrm{rel}} = I \times \frac{1}{I_{0.1}} \qquad \text{(S4)}$$

where $I_{0.1}$ is the fluorescence intensity of 0.1 mol% of **3** (w.r.t lipid) addition after 290 s, considered to be 100 % transport.

## 6.3 Vesicle Preparation for Lucigenin Assays[12]

A thin film of lipid (1-palmitoyl-2-oleoyl-*sn*-3-phosphatidylcholine (POPC) was formed by evaporating a chloroform solution of the lipid under reduced pressure on a rotary evaporator (40 °C) and then under high vacuum for 6 hours. Then the lipid film was hydrated by vortexing with 1 mL of the prepared internal buffer solution. The lipid suspension was then subjected to 5 freeze-thaw cycles using liquid nitrogen and a water bath (40°C) followed by extrusion 19 times through a polycarbonate membrane (pore size 200 nm). Extra-vesicular components were removed by size exclusion chromatography on a Sephadex G-50 column with external buffer solution. The collected vesicles suspension was diluted to 3.5 mL. Final conditions for the standard lucigenin assay (**A**): LUVs (2.5 mM lipid); inside 100 mM $NaNO_3$, 10 mM HEPES, 1 mM lucigenin, pH 7.0; outside: 100 mM $NaNO_3$, 10 mM HEPES, pH 7.0. Final conditions for the coupled-electroneutral transport lucigenin assay (**B**): LUVs (2.5 mM lipid); inside 100 mM $Na_2SO_4$, 20 mM MES, 1 mM *N*,*N'*-dimethyl-9,9'-biacridinium dinitrate (lucigenin), pH 6.5; outside: 100 mM $Na_2SO_4$, 20 mM MES, pH 6.5.

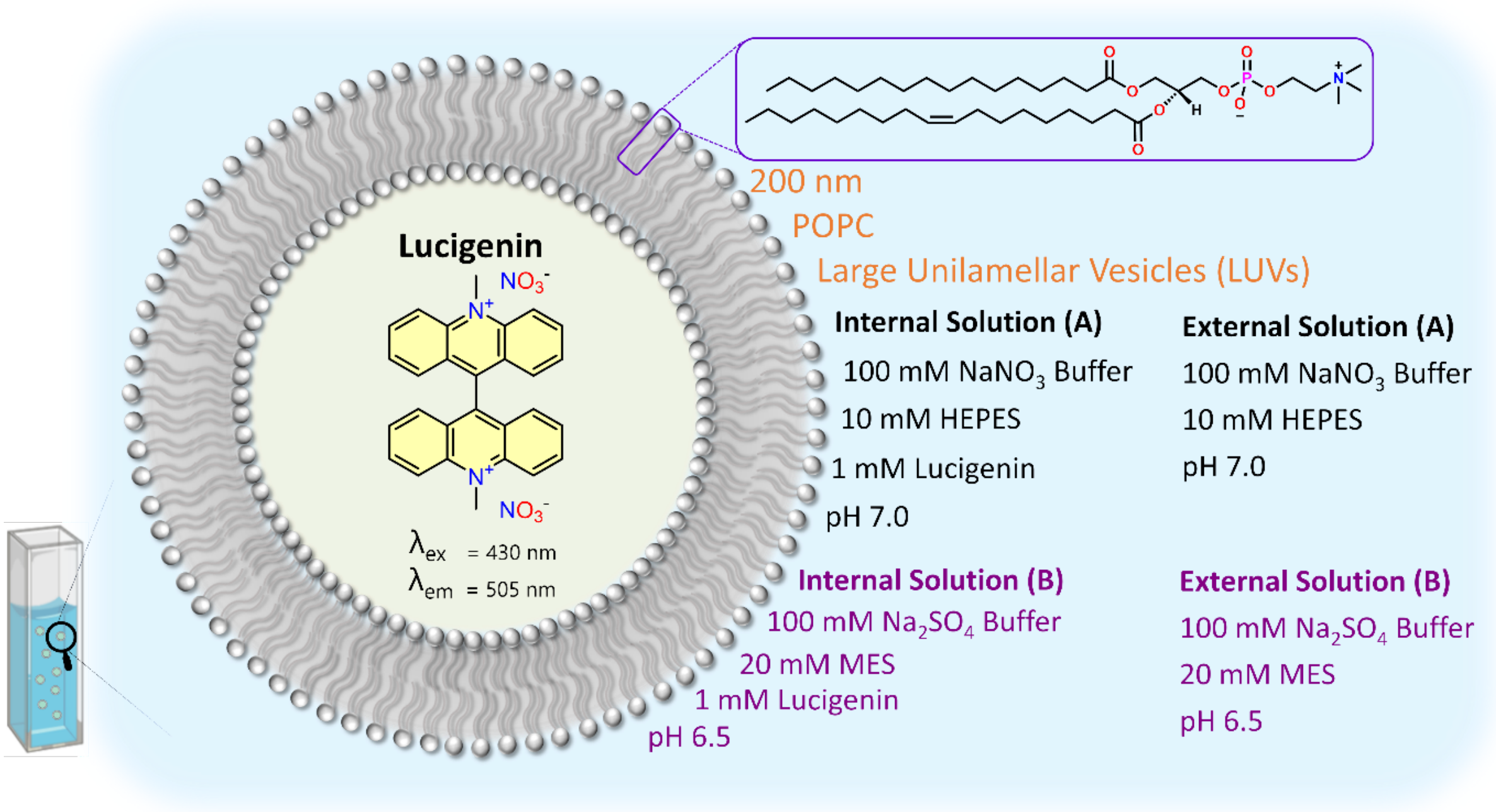


**Figure SI-40:** POPC vesicles encapsulating lucigenin probe (POPC-LUVs⊃Lucigenin) to monitor chloride transport across the lipid membrane with internal and external solutions **(A)**, and chloride transport facilitated by coupled-electroneutral transport **(B)**.

### 6.3.1 The Standard Lucigenin Assay

In a typical experiment, the LUVs containing lucigenin (40 µL, final lipid concentration 33 µM) were added to buffer (2890 µL of 100 mM $NaNO_3$, 10 mM HEPES, pH 7.0) at 25°C under gentle stirring. A pulse of NaCl (20 µL, 0.5 M – final concentration 5 mM) was added at 40 seconds to initiate the experiment. At 90 seconds the test transporter (various concentrations, 20 µL in DMF) was added, followed by detergent (40 µL of Triton X-100 in 7:1 (v/v) $H_2O$-DMSO) at 290 seconds to lyse the vesicles for calibration. The fluorescence intensity, $I_F$, was normalised using Equation S5, where $I_0$ is the initial intensity, $I_t$ is the intensity at time $t$, and $I_\infty$ is the final intensity after addition of Triton X-100.

$$I_F = -\left(\frac{I_t - I_0}{I_\infty - I_0}\right) \qquad \text{(S5)}$$

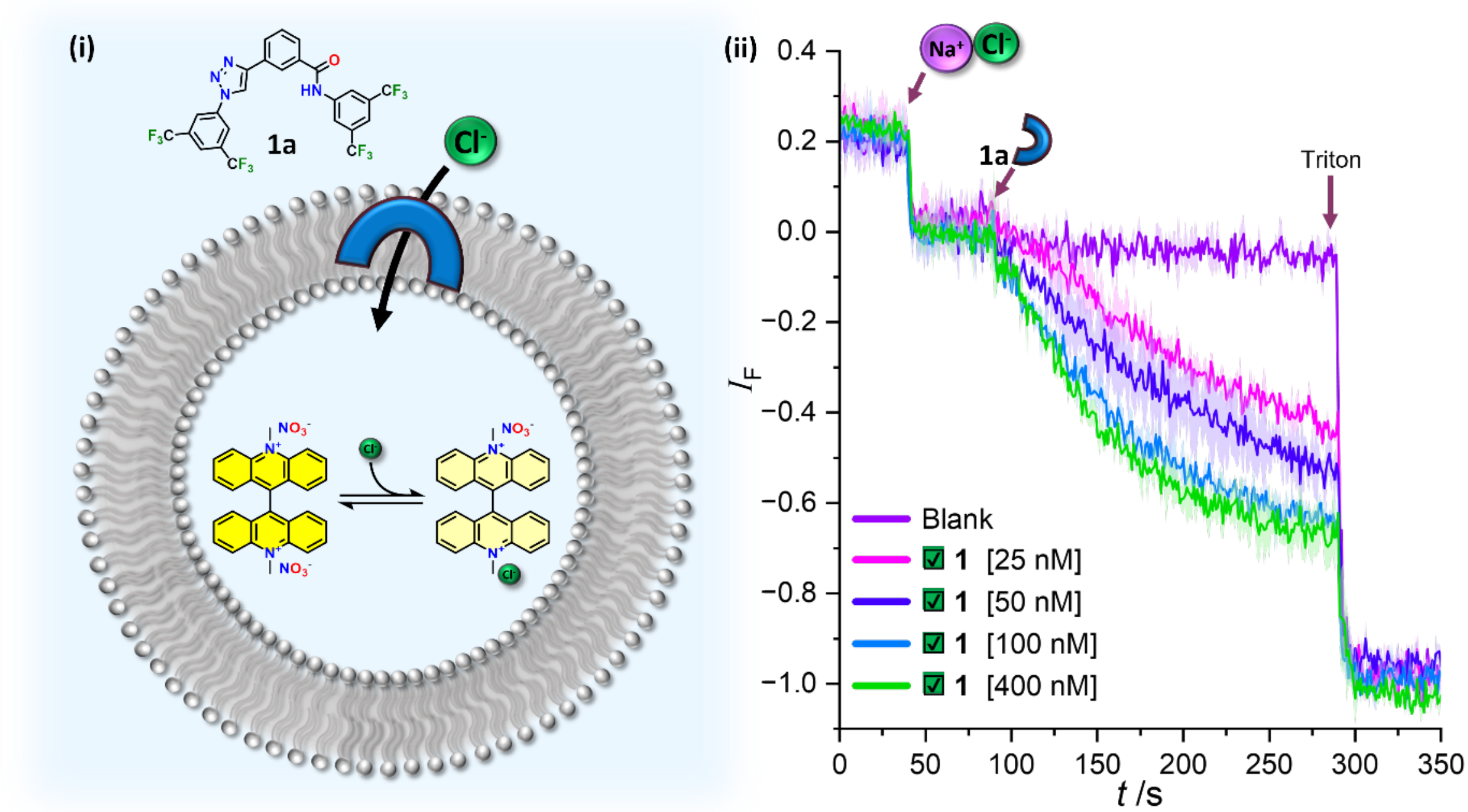


**Figure SI-41: (i)** Schematic diagram of the chloride transport assay with POPC-LUVs⊃lucigenin **(ii)** Change in fluorescence intensity, $I_F$ ($\lambda_{em}$ = 505 nm; $\lambda_{ex}$ = 430 nm) with addition of a NaCl pulse (30 mM), upon addition of anionophore **1a** at increasing concentrations (25 nM, 0.075 mol%; 50 nM, 0.15 mol%; 100 nM, 0.3 mol%; and 400 nM, 1.2 mol% w.r.t lipid) to POPC LUVs (33 µM) containing 1 mM lucigenin, 100 mM internal and external $NaNO_3$, buffered with 10 mM HEPES at pH 7.0.

### 6.3.2 The Coupled-Electroneutral Transport Lucigenin Assay

LUVs containing lucigenin (40 µL, final lipid concentration 33 µM) were added to buffer (2890 µL or 2840 µL of 100 mM $Na_2SO_4$, 20 mM MES, pH 6.5 – final volume in assay 3000 µL) at 25°C under gentle stirring. A pulse of KCl (50 µL, 1.8 M – final concentration 30 mM) or $ZnCl_2$ (100 µL, 0.9 M – final concentration 30 mM) was added at 40 seconds to initiate the experiment. At 60 seconds the first test transporter (10 µL in DMF) was added, followed by the second test transporter (10 µL in DMF) at 80 seconds, and finally the addition of detergent (40 µL of Triton X100 in 7:1 (v/v) $H_2O$-DMSO) at 290 seconds to lyse the vesicles for calibration.

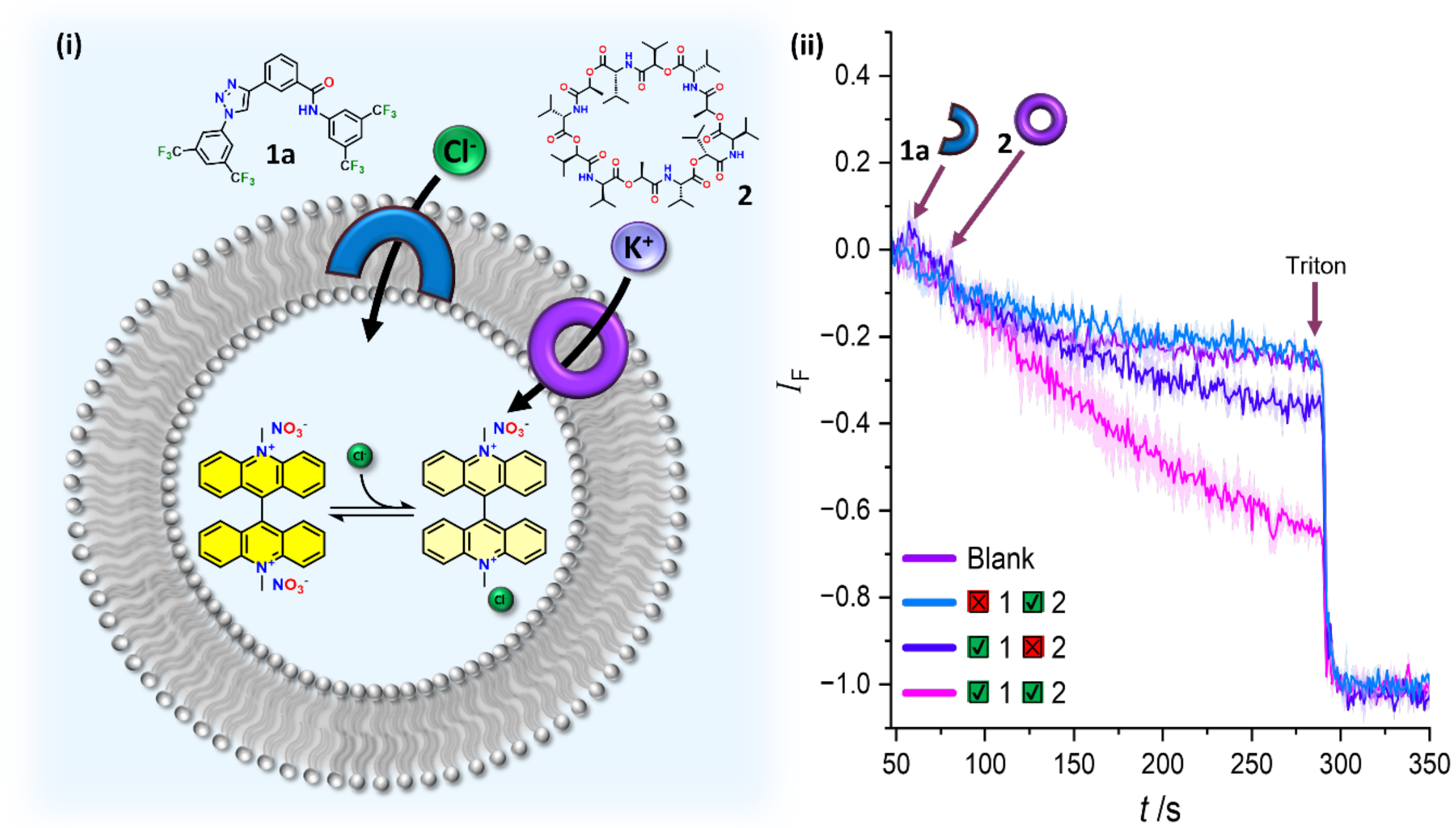


**Figure SI-42: (i)** Schematic diagram of the coupled-electroneutral transport assay with POPC-LUVs⊃lucigenin **(ii)** Change in fluorescence intensity, $I_F$ ($\lambda_{em}$ = 505 nm; $\lambda_{ex}$ = 430 nm) with addition of a KCl pulse (30 mM), upon addition of anionophore **1a** (100 nM, 0.3 mol% w.r.t lipid) and cationophore **2** (100 nM, 0.3 mol% w.r.t lipid) to POPC LUVs (33 µM) containing 1 mM lucigenin, 100 mM internal and external $Na_2SO_4$, buffered with 20 mM MES at pH 6.5.

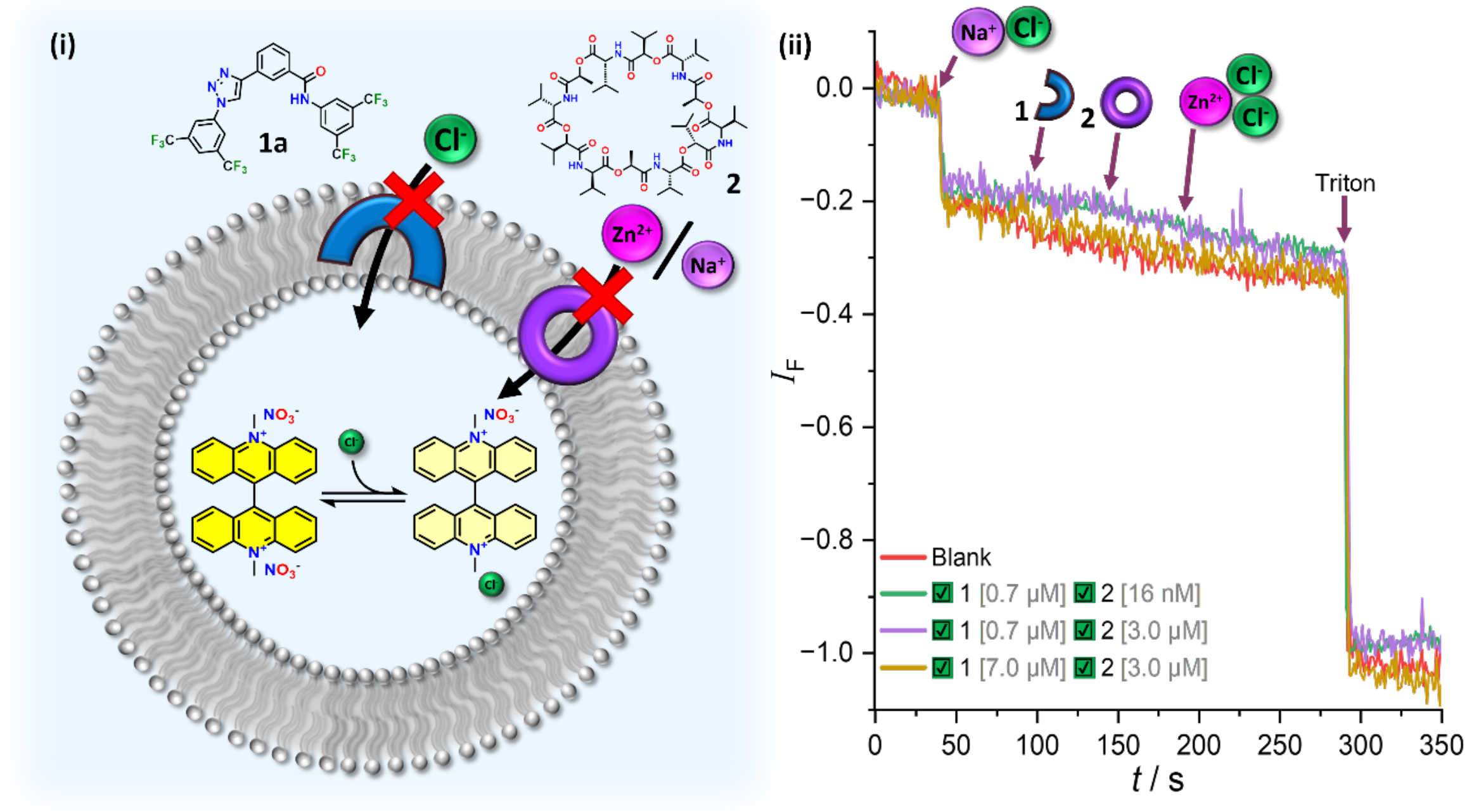


**Figure SI-43: (i)** Schematic diagram of the coupled-electroneutral transport ***control*** assay with POPC-LUVs⊃lucigenin. The highly $K^+$-selective cationophore **2** is unable to facilitate coupled-electroneutral transport in the absence of $K^+$, thereby restricting chloride transport by anionophore **1a**. **(ii)** Change in fluorescence intensity, $I_F$ ($\lambda_{em}$ = 505 nm; $\lambda_{ex}$ = 430 nm) with addition of a KCl pulse (30 mM), upon addition of anionophore **1a** (0.7 µM or 7 µM) and cationophore **2** (16 nM or 3 µM) to POPC LUVs (33 µM) containing 1 mM lucigenin, 100 mM internal and external $Na_2SO_4$, buffered with 20 mM MES at pH 6.5.

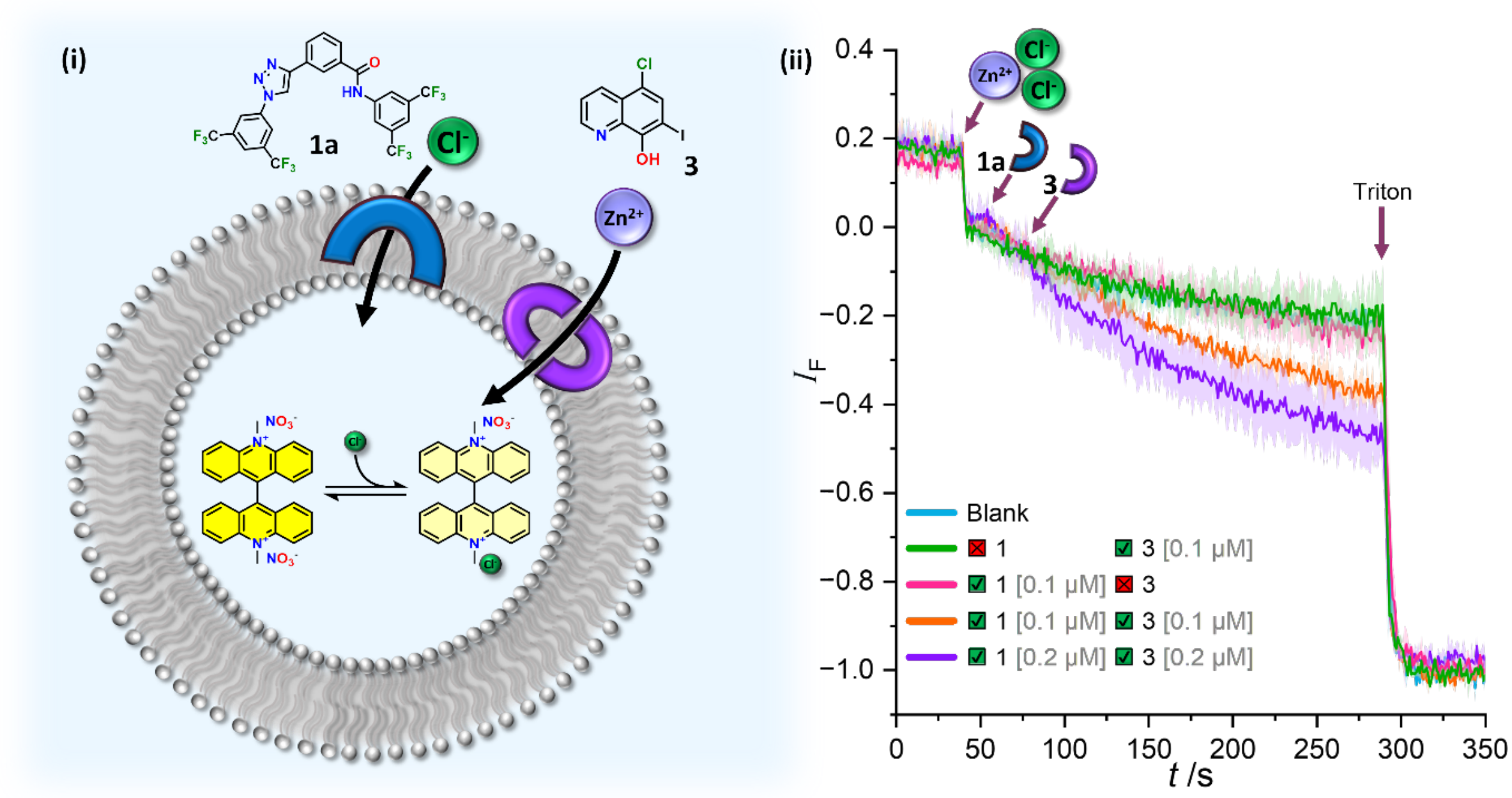


**Figure SI-44: (i)** Schematic diagram of the coupled-electroneutral transport assay with POPC-LUVs⊃lucigenin with anionophore **1a** and cationophore **3**. **(ii)** Change in fluorescence intensity, $I_F$ ($\lambda_{em}$ = 505 nm; $\lambda_{ex}$ = 430 nm) with addition of a $ZnCl_2$ pulse (30 mM), upon addition of anionophore **1a** (0.1 µM or 0.2 µM, 0.3 mol% or 0.6 mol% w.r.t. lipid, respectively) and cationophore **3** (0.1 µM or 0.2 µM, 0.3 mol% or 0.6 mol% w.r.t. lipid, respectively) to POPC LUVs (33 µM) containing 1 mM lucigenin, 100 mM internal and external $Na_2SO_4$, buffered with 20 mM MES at pH 6.5.

## 6.4 Vesicle Preparation for SPQ Assays[13, 14]

A thin film of lipid (1-palmitoyl-2-oleoyl-*sn*-3-phosphatidylcholine (POPC) was formed by evaporating a chloroform solution of the lipid under reduced pressure on a rotary evaporator (40 °C) and then under high vacuum for 6 hours. Then the lipid film was hydrated by vortexing with 1 mL of the prepared internal buffer solution. The lipid suspension was then subjected to 5 freeze-thaw cycles using liquid nitrogen and a water bath (40°C) followed by extrusion 19 times through a polycarbonate membrane (pore size 200 nm). Extra-vesicular components were removed by size exclusion chromatography through two consecutive Sephadex G-25 columns with external buffer solution. Final conditions for the coupled-electroneutral transport SPQ assay: LUVs (2.5 mM lipid); inside 100 mM $Na_2SO_4$, 20 mM MES, 10 mM 6-methoxy-*N*-(3-sulfopropyl)quinolinium (SPQ), pH 6.5; outside: 70 mM $Na_2SO_4$, 20 mM MES, pH 6.5.

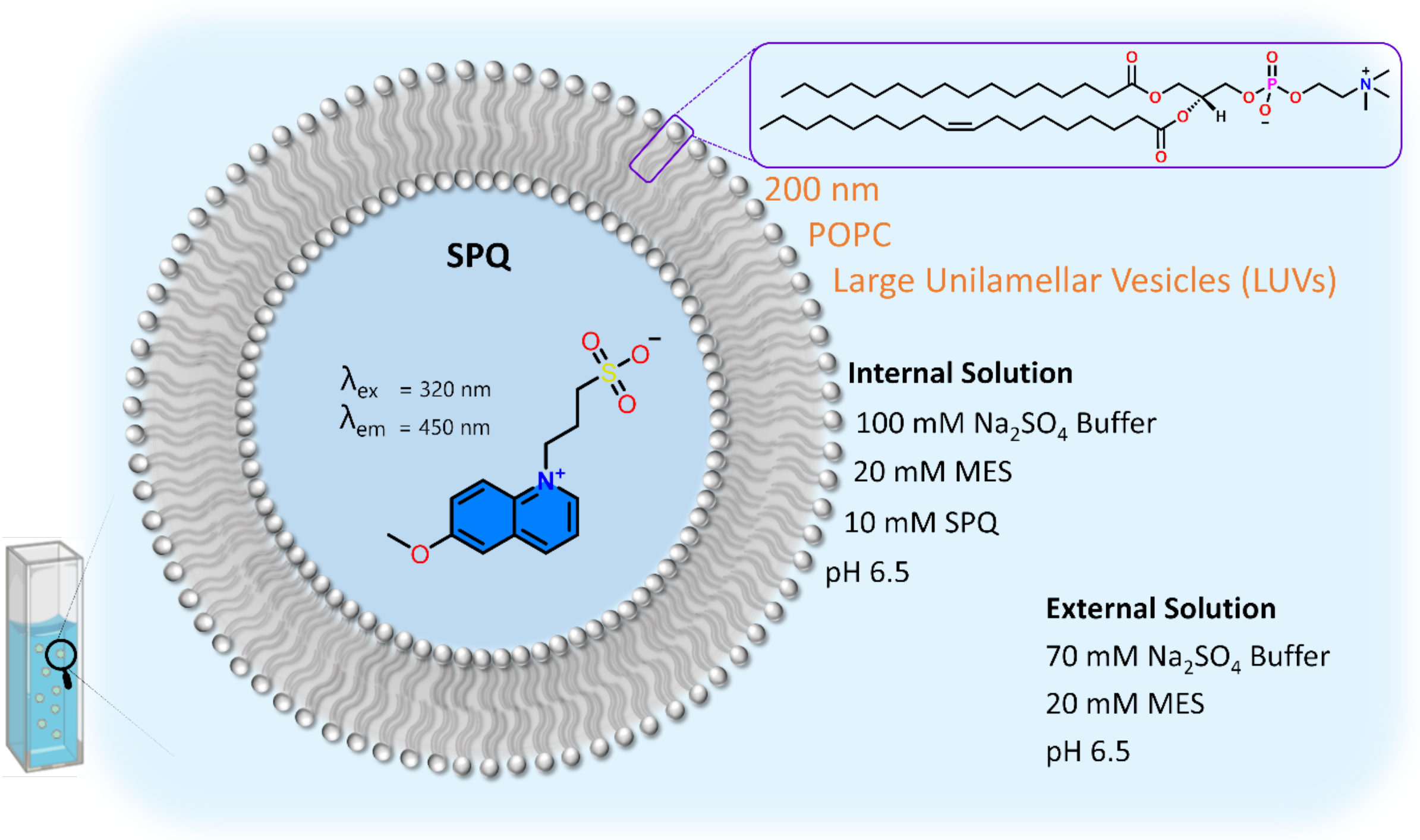


**Figure SI-45:** POPC vesicles encapsulating SPQ probe (POPC-LUVs⊃SPQ) to monitor chloride transport facilitated by coupled-electroneutral transport.

### 6.4.1 The Coupled-Electroneutral Transport SPQ Assay

LUVs containing SPQ (40 µL, final lipid concentration 33 µM) were added to buffer (2840 µL of 100 mM $Na_2SO_4$, 20 mM MES, pH 6.5 – final volume in assay 3000 µL) at 25°C under gentle stirring. Prior to irradiation experiments, coupled-ion transport facilitated by anionophore **1a** and cationophore **3** were tested. **1a** (10 µL in DMF; final concentration 200 nM, 0.6 mol% w.r.t lipid) and **3** (10 µL in DMF; final concentration 200 nM, 0.6 mol% w.r.t lipid) were added to the lipid suspension prior to running the assay, for *in situ* activation, and stirred for 1 minute to allow for thorough distribution. A pulse of $ZnCl_2$ (100 µL, 0.9 M – final concentration 30 mM) was added at 40 seconds to initiate the experiment. At 60 seconds the first test transporter (10 µL in DMF) was added, followed by the second test transporter (10 µL in DMF) at 80 seconds, and finally the addition of detergent (40 µL of Triton X100 in 7:1 (v/v) $H_2O$-DMSO) at 290 seconds to lyse the vesicles for calibration. The fluorescence intensity, $I_F$, was normalised using Equation S5.

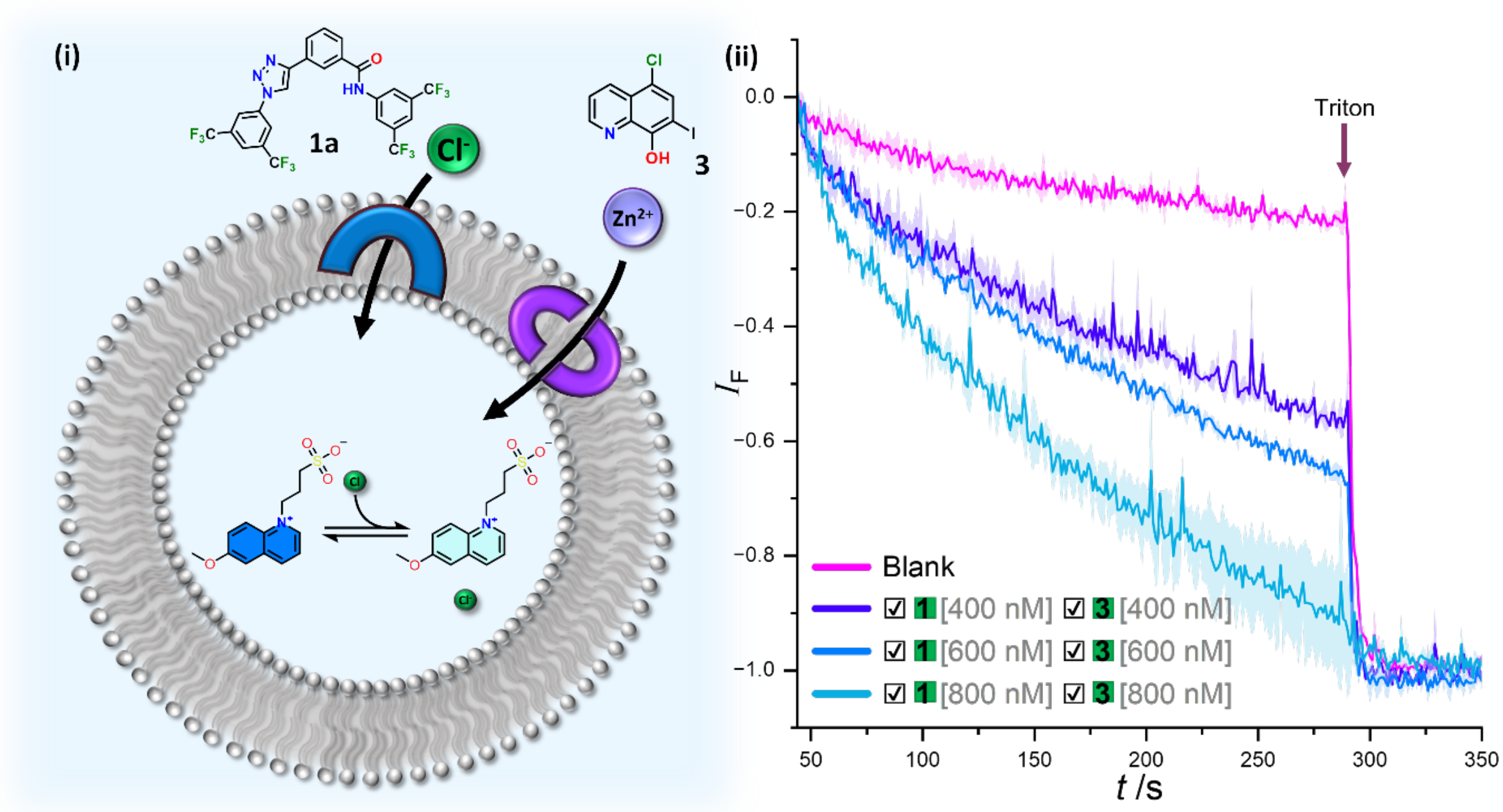


**Figure SI-46: (i)** Schematic diagram of the coupled-electroneutral transport assay with POPC-LUVs⊃SPQ with anionophore **1a** and cationophore **3**. **(ii)** Change in fluorescence intensity, $I_F$ ($\lambda_{em}$ = 450 nm; $\lambda_{ex}$ = 320 nm) with *in situ* activation of anionophore **1a** and cationophore **3** at increasing concentrations (400 nM, 1.2 mol% w.r.t lipid; 600 nM, 1.8 mol%; and 800 nM, 2.4 mol% w.r.t lipid) upon addition of a $ZnCl_2$ pulse (30 mM) to POPC LUVs (33 µM) containing 1 mM SPQ, 100 mM internal and 70 mM external $Na_2SO_4$, buffered with 20 mM MES at pH 6.5.

## 6.5 Photo-triggered Transport Assays

The HPTS, MgG, or SPQ assays were modified to allow for *in situ* irradiation of the photocaged transporters, and was carried out as follows: the desired concentration of the photocaged transporters in DMSO or THF was added to the lipid suspension and stirred for 1 minute to equilibrate. The sample was then irradiated with a Thorlabs mounted LED in a Thorlabs cuvette holder (CVH100/MM). The LEDs were supplied with 1 A current using Thorlabs T-cub LED driver (LEDD1B). The transport assay was initiated with a base pulse of NaOH (HPTS assay) or a $ZnCl_2$ pulse (MgG and SPQ assays) and the fluorescence emission was monitored over time, before calibration with Triton-X-100 (HPTS and SPQ assays) or 0.1 mol% w.r.t lipid (MgG assay). The photo-triggered transport activity was monitored with increasing irradiation times.

Photo-triggered transport assays were also conducted by *ex situ* irradiation of a vial containing a stock solution of the photocaged transporters in a DMSO or THF solution. The irradiated sample was added to the lipid suspension after the 40 second pulse, as per the typical HPTS (Section 6.1), MgG (Section 6.2), or SPQ (Section 6.4) assays.

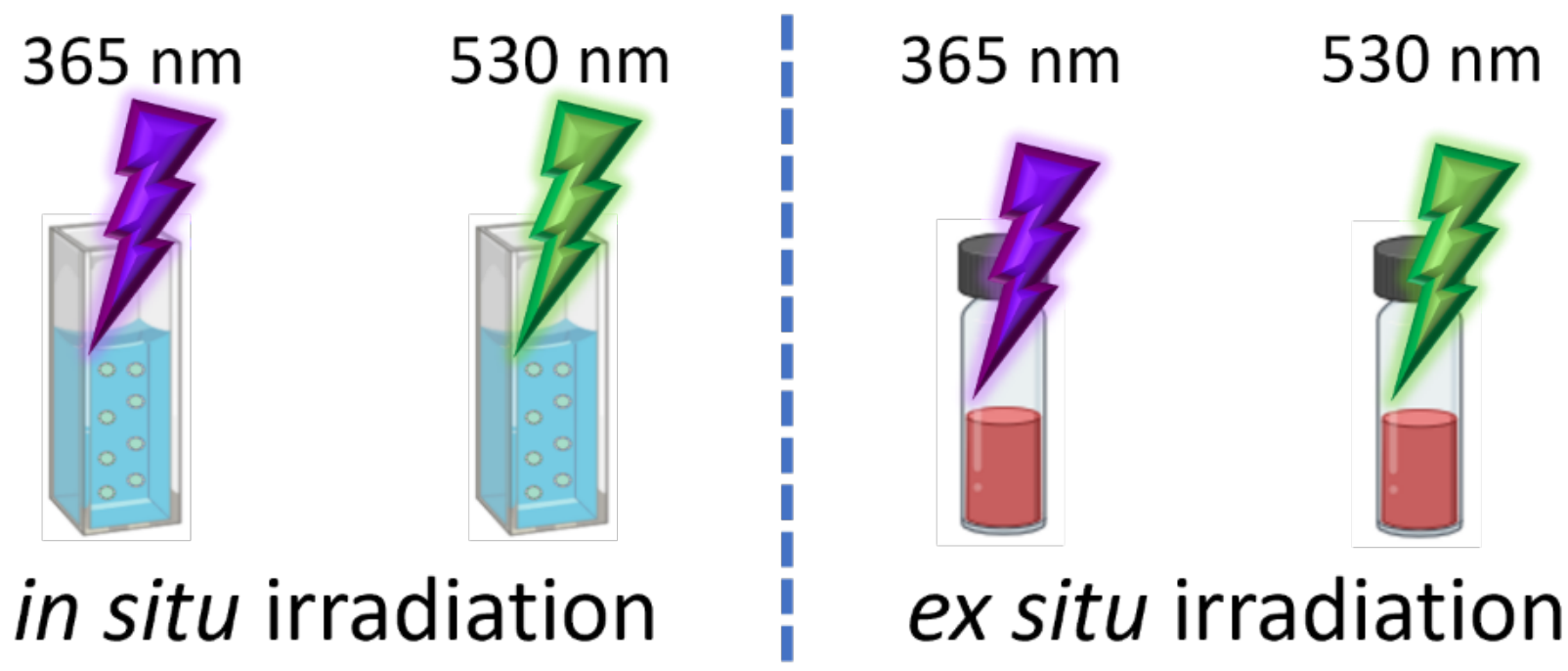


**Figure SI-47:** Schematic depiction of *in situ* irradiation in a cuvette containing the photocaged transporter and POPC LUVs in buffer (**LEFT**) and *ex situ* irradiation of a vial containing stock solution of photocaged transporters in DMSO or THF (**RIGHT**), at different wavelengths.

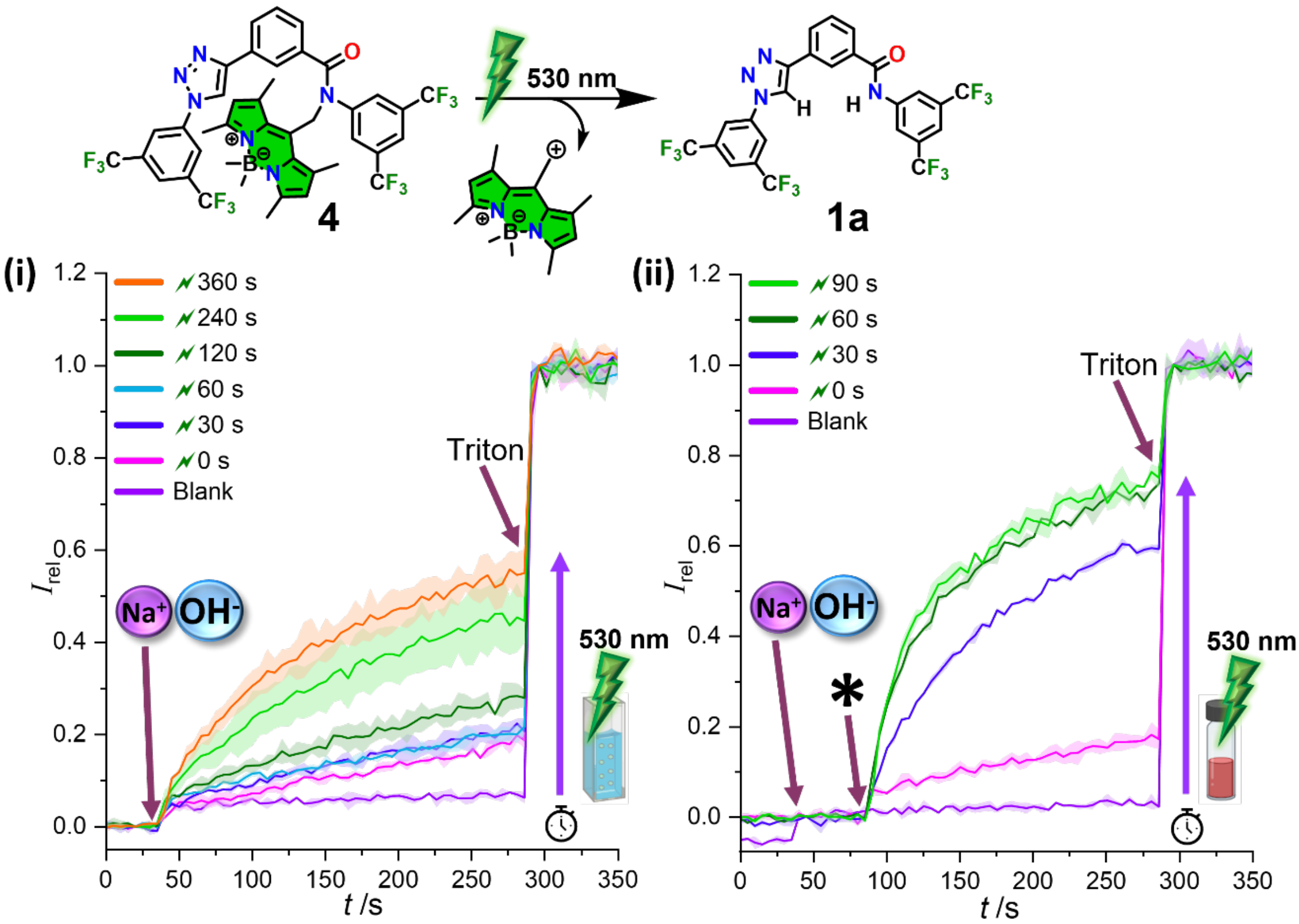


**Figure SI-48:** Change in ratiometric emission, $I_{rel}$ ($\lambda_{em}$ = 510 nm; $\lambda_{ex1}$ = 405 nm, $\lambda_{ex2}$ = 460 nm) with increasing irradiation time of BODIPY-photocaged anionophore **4** (78 nM, 0.25 mol% w.r.t lipid) *in situ* **(i)** and *ex situ* **(ii)** with a 530 nm LED (370 mW), upon addition of a NaOH base pulse (5 mM) to POPC LUVs (31 μM) containing 1 mM HPTS, 100 mM internal and external NaCl, buffered with 10 mM HEPES at pH 7.0. * Indicates addition of photo-irradiated sample **4**.

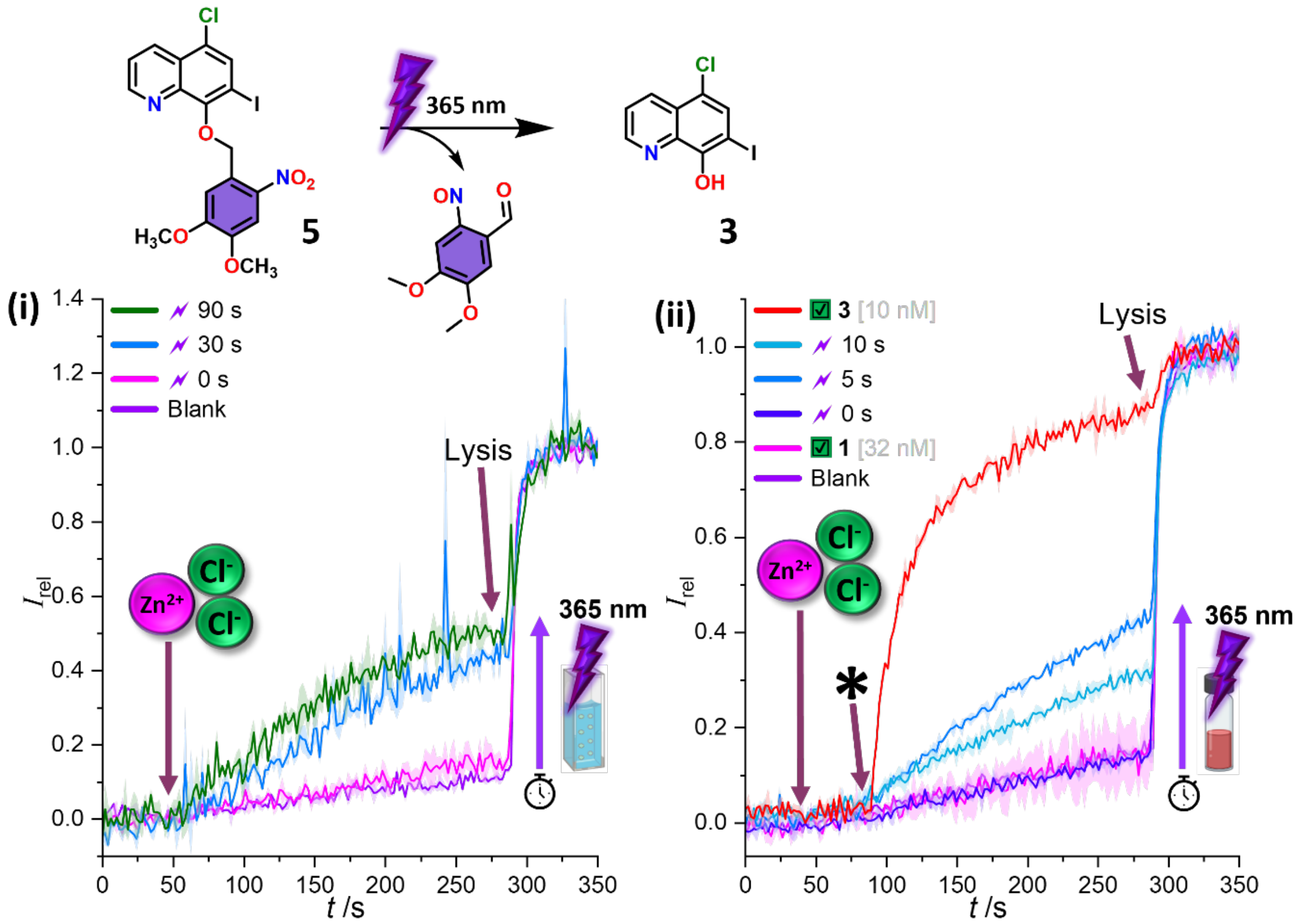


**Figure SI-49:** Change in MgG fluorescence intensity, $I_{rel}$ ($\lambda_{em}$ = 531 nm; $\lambda_{ex}$ = 506 nm) with increasing irradiation time of DMNB-photocaged cationophore **5** (10 nM, 0.01 mol% w.r.t lipid) *in situ* **(i)** and *ex situ* **(ii)** with a 365 nm LED (880 mW), upon addition of a $ZnCl_2$ pulse (100 µM) to POPC LUVs (100 µM) containing 50 µM MgG, 10 µM EDTA, 100 mM internal and external NaCl, buffered with 10 mM HEPES at pH 7.0. No zinc transport was observed with the addition of anionophore **1a** (32 nM, 0.03 mol% w.r.t lipid).

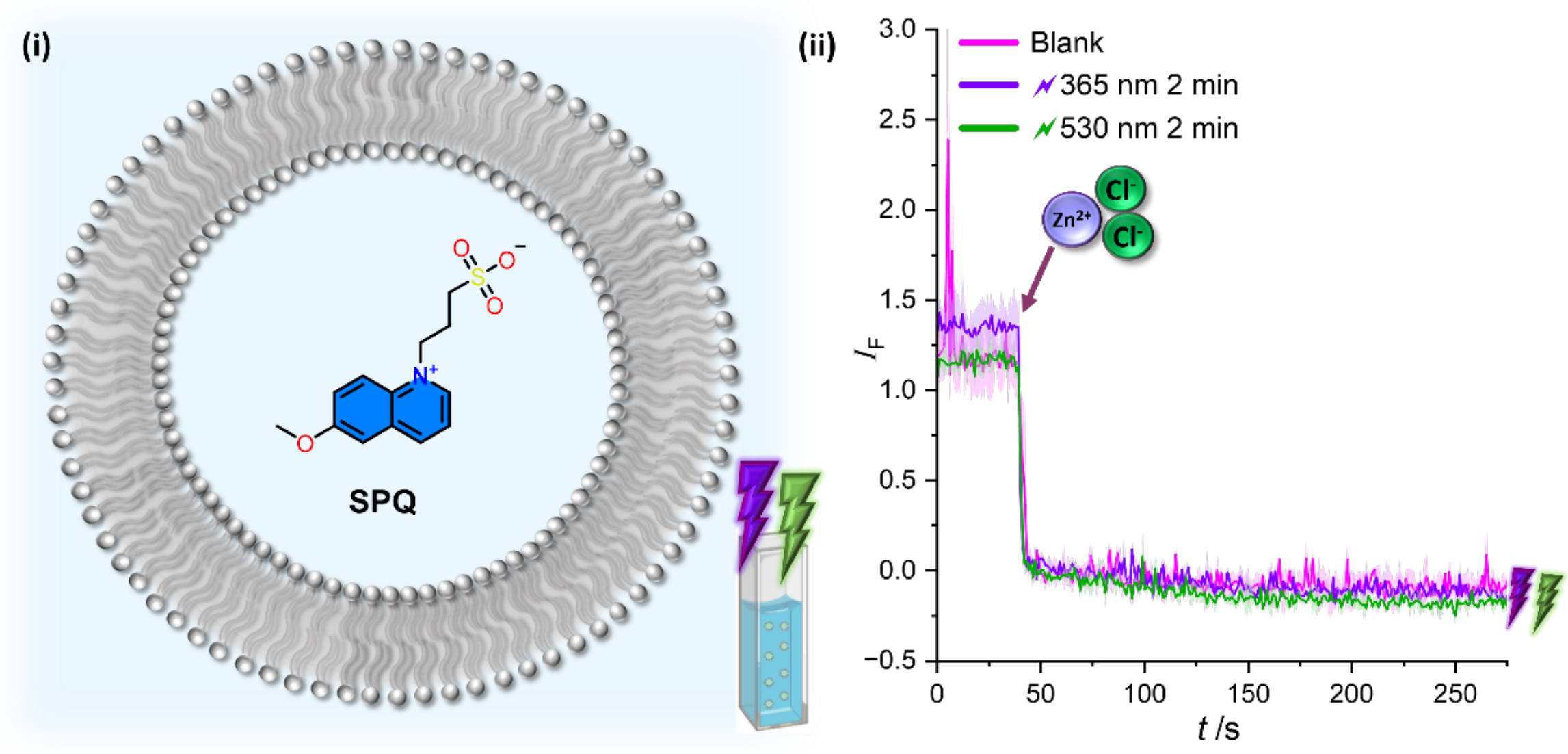


**Figure SI-50: (i)** Schematic diagram of POPC-LUVs⊃SPQ irradiated *in situ* to test viability of the SPQ probe. **(ii)** Change in fluorescence intensity, $I_F$ ($\lambda_{em}$ = 450 nm; $\lambda_{ex}$ = 320 nm) following *in situ* irradiation for 2 minutes with a 365 nm LED (880 mW) or a 530 nm LED (370 mW), and plotted against a blank sample that was not irradiated. At 40 seconds, a $ZnCl_2$ pulse (30 mM) was added to the POPC LUVs (33 µM) containing 1 mM SPQ, 100 mM internal and 70 mM external $Na_2SO_4$, buffered with 20 mM MES at pH 6.5.

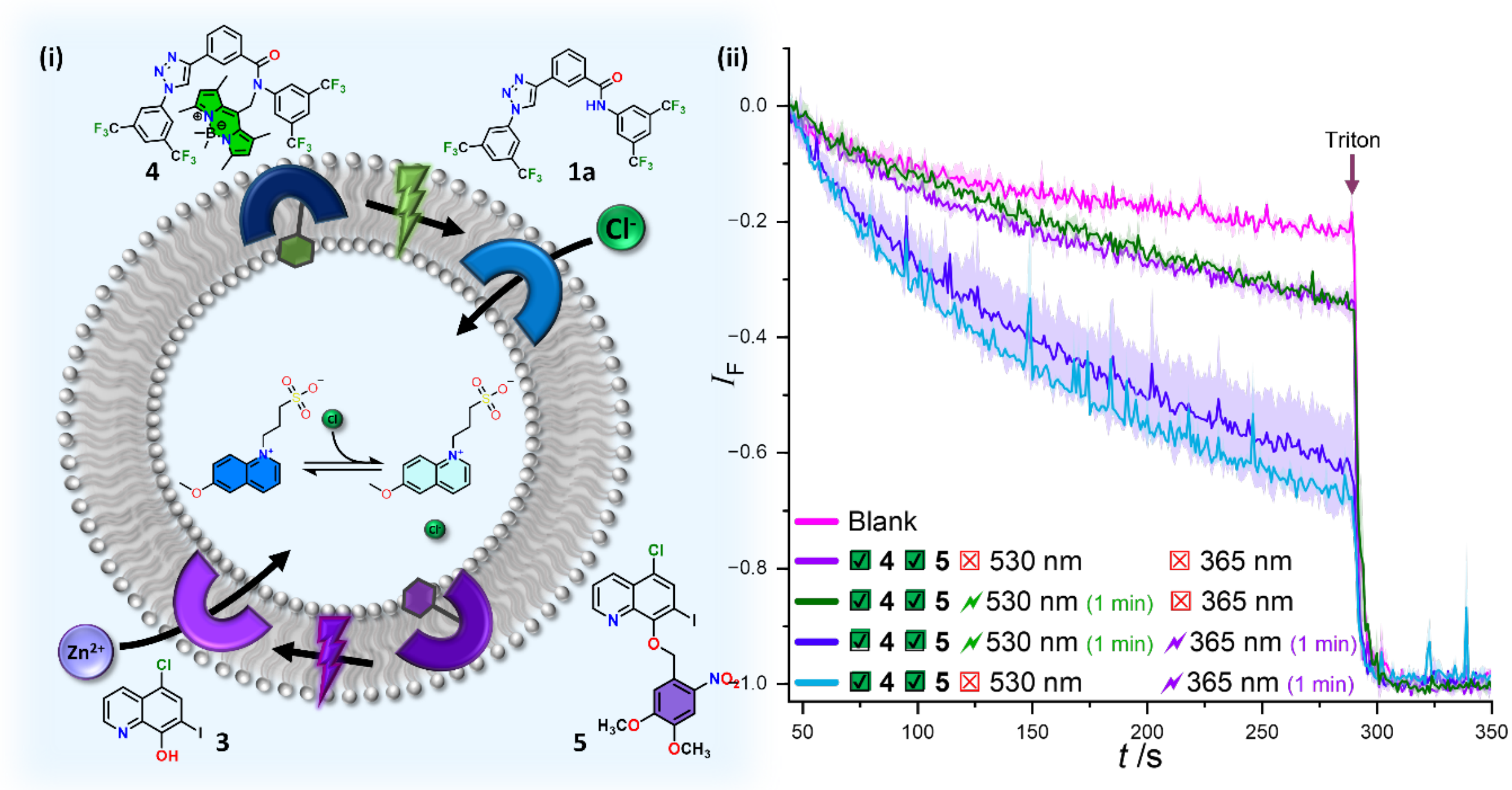


**Figure SI-51: (i)** Schematic diagram of multiwavelength activation of the coupled-electroneutral transport assay with POPC-LUVs⊃SPQ. **(ii)** Change in fluorescence intensity, $I_F$ ($\lambda_{em}$ = 450 nm; $\lambda_{ex}$ = 320 nm) following *in situ* irradiation with a 365 nm LED (880 mW) and/or a 530 nm LED (370 mW) of BODIPY-photocaged anionophore **4** (400 nM, 1.2 mol% w.r.t. lipid) and DMNB-photocaged cationophore **5** (400 nM, 1.2 mol% w.r.t. lipid), and plotted with a blank sample that was not irradiated, and **4** and **5** prior to any irradiation. At 40 seconds, a $ZnCl_2$ pulse (30 mM) was added to the POPC LUVs (33 µM) containing 1 mM SPQ, 100 mM internal and 70 mM external $Na_2SO_4$, buffered with 20 mM MES at pH 6.5.

# 7 References


1. C. Damiano, S. Gadolini, D. Intrieri, L. Lay, C. Colombo and E. Gallo, *Eur. J. Inorg. Chem.*, 2019, **41**, 4412–4420.
2. X. Liu, A. Kung, B. Malinoski, G. K. S. Prakash and C. Zhang, *J. Med. Chem.*, 2015, **58**, 9228–9237.
3. K. Sitkowska, B. L. Feringa and W. Szymański, *J. Org. Chem.*, 2018, **83**, 1819–1827.
4. P. Shrestha, A. Mukhopadhyay, K. C. Dissanayake and A. H. Winter, *J. Org. Chem.*, 2022, **87**, 14334–14341.
5. J. A. Peterson, L. J. Fischer, E. J. Gehrmann, P. Shrestha, D. Yuan, C. S. Wijesooriya, E. A. Smith and A. H. Winter, *J. Org. Chem.*, 2020, **85**, 5712–5717.
6. K. Krumova and G. Cosa, *J. Am. Chem. Soc.*, 2010, **132**, 17560–17569.
7. S. A. Gartland, T. G. Johnson, E. Walkley and M. J. Langton, *Angew. Chem. Int. Ed. Engl.*, 2023, **62**, e202309080.
8. BindFit v0.5, http://app.supramolecular.org/bindfit/).
9. D. Brynn Hibbert and P. Thordarson, *Chem. Commun.*, 2016, **52**, 12792–12805.
10. A. Kerckhoffs and M. J. Langton, *Chem Sci*, 2020, **11**, 6325–6331.
11. A. M. Gilchrist, P. Wang, I. Carreira-Barral, D. Alonso-Carrillo, X. Wu, R. Quesada and P. A. Gale, *Supramol. Chem.*, 2021, **33**, 325–344.
12. M. Chvojka, A. Singh, A. Cataldo, A. Torres-Huerta, M. Konopka, V. Šindelář and H. Valkenier, *Analysis & Sensing*, 2024, **4**, e202300044.
13. C. Ren, X. Ding, A. Roy, J. Shen, S. Zhou, F. Chen, S. F. Yau Li, H. Ren, Y. Y. Yang and H. Zeng, *Chem. Sci.*, 2018, **9**, 4044–4051.
14. B. Zawada and M. J. Chmielewski, *Org. Biomol. Chem.*, 2024, **22**, 7143–7147.
